# Cardamonin suppresses mTORC1/SREBP1 through reducing Raptor and inhibits *de novo lipogenesis* in ovarian cancer

**DOI:** 10.1371/journal.pone.0322733

**Published:** 2025-05-02

**Authors:** Peiguang Niu, Danyun Li, Huajiao Chen, Yanting Zhu, Jintuo Zhou, Jinhua Zhang, Ying Liu

**Affiliations:** 1 Department of Pharmacy, Fujian Maternity and Child Health Hospital, College of Clinical Medicine for Obstetrics and Gynecology and Pediatrics, Fujian Medical University, Fuzhou, Fujian, China; 2 Fujian Key Laboratory of Women and Children’s Critical Diseases Research [Fujian Maternity and Child Health Hospital (Fujian Women and Children’s Hospital)], Fujian Maternity and Child Health Hospital, Fuzhou, Fujian, China; Southern Illinois University School of Medicine, UNITED STATES OF AMERICA

## Abstract

Metabolic reprogramming is a hallmark of cancer and *de novo lipogenesis* (DNL) accelerates the progression of ovarian cancer. In this study, we investigated the effects of cardamonin, a natural compound potential to suppress various malignancies, on the lipid anabolism in ovarian cancer. Cell proliferation was assessed using CCK-8 and clone formation assay. Cell apoptosis was detected by flow cytometry with Annexin V-FITC/PI staining and mitochondrial membrane potential (MMP) was measured with JC-10 probe. Free fatty acids (FFA) was measured by fluorescence using acyl-CoA oxidation and carnitine palmitoyl transferase-1 (CPT-1) activity was analyzed by spectrophotometric assay using palmitoyl-CoA and DTNB (5,5’-dithio-bis-(2-nitrobenzoic acid)) reaction. mRNA expression was measured by Quantitative Real-Time PCR. Protein expression was analyzed through western blotting and immunofluorescence. Raptor was knocked down by shRNA and Raptor was overexpressed by lentiviral transfection. The antitumor effect of cardamonin was evaluated using a xenotransplantation tumor bearing mouse model. Cardamonin suppressed the cell proliferation, induced cell apoptosis and triggered mitochondrial damage in ovarian cancer cells. Cardamonin inhibited the protein expression of sterol regulatory element binding protein 1 (SREBP1) and its downstream lipogenic enzymes and decreased FFA content and CPT-1 activity. Additionally, cardamonin inhibited the activation of mechanistic target of rapamycin complex 1 (mTORC1) and expression of regulatory-associated protein of mTOR (Raptor). Raptor knockdown abolished the inhibitory effect of cardamonin on mTORC1 and SREBP1. Furthermore, cardamonin inhibited mTORC1 activation and lipogenic proteins expression induced by Raptor overexpression. Cardamonin reduced the tumor growth and fatty acid synthase of the tumors, as evidenced by decreased expression of Ki-67 and FASN. It suggests that cardamonin suppresses mTORC1/SREBP1 through reducing the protein level of Raptor and inhibits DNL of ovarian cancer.

## Introduction

Epithelial ovarian cancer is a common gynecological malignancy with the highest mortality rate among women [1]. The current standard treatment for ovarian cancer involves cytoreductive surgery combined with platinum-based chemotherapy. However, owing to the challenges of late-stage diagnosis and cytotoxicity of chemotherapy drugs, the 5-year survival rate of patients with ovarian cancer remains low [2]. Therefore, it is crucial to explore the molecular pathogenesis and identify targeted drugs to improve the prognosis of patients with ovarian cancer.

Metabolic reprogramming is considered as a hallmark of cancer, where malignant cells adapt to the demands of rapid cell proliferation, by relying on aerobic glycolysis, fatty acid synthesis and glutamine metabolism for survival [3]. Notably, *de novo lipogenesis* (DNL) plays a crucial role in the progression and metastasis of ovarian cancer [4]. In this process, ATP-citrate lyase (ACLY) catalyzes the conversion of citrate into acetyl-CoA in the cytosol, which is then carboxylated by acetyl CoA carboxylase (ACC) to form malonyl-CoA. Subsequently, fatty acid synthase (FASN) catalyzes fatty acid synthesis from malonyl-CoA [5]. Fatty acids are essential components of biological membranes and serve as key carbon sources for the tricarboxylic acid cycle and oxidative phosphorylation. Consequently, DNL supplies cancer cells with precursors for cell membrane synthesis and acts as an energy source, thereby supporting their rapid proliferation [6].

The mechanistic target of rapamycin (mTOR) is frequently upregulated during metabolic reprogramming [7]. mTOR functions as two distinct complexes, mTOR complex 1 (mTORC1) and mTOR complex 2 (mTORC2), with different catalytic components. The core subunits of mTORC1 consist of mTOR, regulatory-associated protein of mTOR (Raptor) and mammalian lethal with SEC13 protein 8 [8,9]. Raptor plays a crucial role as the uniquely defined subunit that is essential for the activation of mTORC1. Cryo-electron microscopy assays have demonstrated that Raptor is also responsible for the recruitment and activation of mTORC1 substrates, including p70 S6 kinase 1 (S6K1) and eukaryotic initiation factor 4E (eIF4E) binding protein 1 (4E-BP1) [10,11].

Recently, the metabolic function of mTORC1 gains more prominence. mTORC1 activation is associated with increased lipogenesis, whereas its inhibition increases lipolysis and fatty acid oxidation. mTORC1 promotes lipid biosynthesis by regulating the expression of lipogenic genes. The key transcription factor for lipid synthesis-related genes is sterol regulatory element binding protein 1 (SREBP1). At the transcription level, mTORC1 modulates the expression of lipogenic enzymes by regulating SREBP1 [12,13]. Rapamycin, the classical mTOR inhibitor, suppresses the expression of SREBP1 and its downstream target genes *ACC, FASN*, and *ACLY* [14]. Rapamycin and its derivates (rapalogs) are used to treat patients with advanced renal cell carcinoma, breast cancer and pancreatic cancer. However, the clinical application of rapalogs is limited due to serious adverse reactions and immunosuppressive effects [15]. Additionally, amino acid mutations in the mTOR protein have led to the emergence of mTOR inhibitor resistance [16], highlighting the need for novel mTOR inhibitors. The mTOR-Raptor interaction promotes lipogenesis by increasing the protein expression of SREBP1, whereas in Raptor knockout cells, SREBP1-dependent lipogenesis is significantly decreased [13]. This indicates that the promotion of DNL by mTORC1 is associated with Raptor.

**Fig 1:**
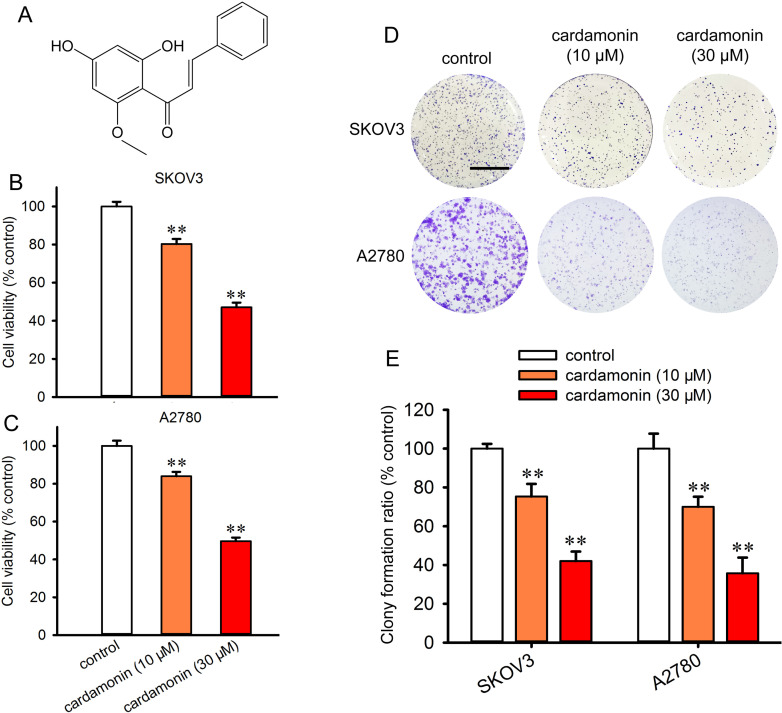
Cardamonin inhibited the proliferation of ovarian cancer cells. (A) The chemical structure of cardamonin. (B, C) SKOV3 and A2780 were treated with 10 and 30 μM of cardamonin for 48 h; cell viability was assessed the CCK-8 assay (*n = 5*). (D) Then the effect of cardamonin on the colony formation ratio of SKOV3 and A2780 cells were detected (*n = 3*). (D) The representative images of cell clony. Scale bar: 1 cm. (E) The colony numbers were counted and clony formation ratio was shown by the diagram (*n = 3*). The formation ratio was normalized to control. Control group presented ovarian cancer cells without any treatment. All the data were expressed as means ± SD. ^**^P < 0.01 compared with control.

Cardamonin is a flavonoid extracted from the dry seeds of *Amomum subulatum* (Fig 1A). It exhibits various pharmacological activities, including anti-tumor and anti-inflammatory effects [17]. Our previous studies demonstrate that cardamonin inhibits the proliferation of ovarian cancer cells by disrupting the cell cycle and inducing cell apoptosis [18]. Additionally, we find that the anti-tumor effects of cardamonin are associated with the inhibition of mTORC1. Unlike rapalogs and the ATP-competitive mTOR inhibitor (AZD8055), cardamonin suppresses mTORC1 by reducing the protein expression of Raptor [19,20]. Seo et al. demonstrate that cardamonin decreases lipogenesis and promotes lipolysis in 3T3-L1 cells [21]. However, the effect of cardamonin on lipid anabolism in cancer cells remains unknown. In this study, we investigate the effects of cardamonin on DNL in ovarian cancer cells by targeting Raptor.

## Materials and methods

### Reagents

Antibodies against to phospho-S2448 mTOR (#2971), mTOR (#2972), phospho-T389 S6K1 (#9205), S6K1 (#9202), phospho-T37/46 4E-BP1 (#2855), 4E-BP1 (#9452), Raptor (#2280), FASN (#3189), ACC (#3662), ACLY (#4332), β-actin (#8457) and anti-rabbit (#7074) or anti-mouse (#7076) secondary antibody were purchased from Cell Signaling Technology (Danvers, MA, USA). Antibody against to SREBP1 (#sc-13551) was purchased from Santa Cruz Biotechnology (Dallas, TX, USA). Antibodies against to Ki-67 (ab279653), FASN (ab128870), Raptor (ab40768) for immunohistochemistry and the Free Fatty Acid Assay Kit (ab65341) was from Abcam (Cambridge, MA, USA). Cardamonin (#C8249) and rapamycin (#553211) were purchased from Sigma-Aldrich and Merck KGaA (Darmstadt, Germany). AZD8055 (#HY-10422) was purchased from MedChemExpress (Shanghai, China).

### Cell culture

Human ovarian SKOV3 and A2780 cells were obtained from Boster Biological Technology (Wuhan, China) and Fenghui Biotechnology (Changsha, China), respectively. SKOV3 and A2780 cells were cultured with McCoy’s 5A and RPMI1640 media which supplemented with 2 mM glutamine, 10% fetal bovine serum (FBS), 100 U/mL penicillin and 100 μg/mL streptomycin. The cells were maintained at 37°C in a humidified 5% CO_2_ atmosphere. All the media and supplements for cell culture were purchased from Gibco (Grand Island, NY, USA).

### Cell viability assay

Cell Counting Kit-8 (CCK-8) assay was employed to determine the cell viability. 5 × 10^3^ cells/well were seeded on 96-well plate and allowing the cells to attach overnight. Then the culture media was replaced with treatment media containing 2.5, 5, 10, 20, 40, 80 μM of cardamonin and cultured for 48 h. 10 μL of CCK-8 (#96992, Sigma Aldrich) solution was added into each well and the plates were incubated for 2 h in the incubator at 37°C, and then the optical density was measured at 450 nm using a microplate reader. The inhibition rate and IC50 of cardamonin on SKOV3 and A2780 was calculated. For the following experiments, ovarian cancer cells were seeded in the culture plate or culture dish and allowing the cells to attach overnight. Then the culture media was replaced with treatment media containing 10 μM, 30 μM of cardamonin and cultured for 48 h. In parallel, SKOV3 and A2780 cells grown in normal media were set as control group.

### Colony formation assay

1 × 10^3^ cells/well were seeded into 6-well plate and allowing the cells to attach overnight. Following treatment with cardamonin (10, 30 μM) for 48 h, then the medium was replaced with the fresh media. After 10 days of culture, the cell colonies were fixed with 4% paraformaldehyde and stained with 0.1% crystal violet solution. The number of the colonies was counted.

### Annexin V-FITC/PI apoptosis assay

Cell apoptosis was detected by the Annexin V-FITC/PI apoptosis detection kit (BD, San Diego, CA, USA). Treat with cardamonin for 48 h, cells were collected and washed with ice cold PBS twice. And then the cells were resuspended with binding buffer at a concentration of 1 × 10^6^ cells/mL. Annexin V/FITC and PI staining solution (5 μL) was added into 500 μL cell suspension, and incubated at room temperature for 15 min in dark and then analyzed by the BD LSRFortessa flow cytometry (BD Biosciences, San Jose, CA, USA).

### Mitochondrial membrane potential (MMP) measurement

MMP was evaluated using the JC-10 Mitochondrial Membrane Potential Assay Kit (ab112133; Abcam). Treat with cardamonin for 48 h, cells were collected and washed with cold PBS twice. 5 × 10^5^ cells were suspended in 500 µl of 1 × JC-10 dye-loading solution and incubated at room temperature for 30 min in dark. Then, the cells were analyzed using the BD LSRFortessa flow cytometry (BD Biosciences, San Jose, CA, USA).

### Determination of free fatty acids

Free fatty acids content was determined by Free Fatty Acid Assay Kit from Abcam (#ab65341). 1 × 10^6^ cells were harvested and washed with ice cold PBS for three times. And then cells were homogenized with 1% Triton X-100 in chloroform and incubate on ice for 20 min. The organic phase was separated by centrifugation, followed by air-drying at 50 °C for 40 min. The dried lipids were dissolved in Fatty Acid Assay Buffer and converted into acyl-CoA by incubating with 2 μL of Acyl-CoA Synthetase Reagent for 30 min at 37 °C in a black 96-well plate. Then, 50 μL of reaction buffer was added into each well to oxidize acyl-CoA. The fluorescence of the resultant product was measured using Spark™ 10M multimode microplate reader at Ex/Em = 535/587 nm [22].

### Determination of carnitine palmitoyl transferase-1 (CPT-1) activity

CPT-1 activity was determined as previously described [5]. Treated with indicated drugs for 48 h, mitochondrial proteins were isolated by lysis buffer (Tris-HCl pH 7.4) containing 0.25 mM sucrose and 1 mM EDTA. The mitochondrial protein content was determined and added the reaction mix containing Tris buffer (100 mM, pH 8.0, 0.1% Triton-X-100, 1 mM EDTA), 0.01 mM palmitoyl-CoA, and 0.5 mM DTNB. After adding 1.25 mM L-carnitine, the optical density was measured at 412 nm.

### Quantitative real-time PCR (RT-PCR)

Total RNA of was extracted using Trizol reagent (#15596026, Invitrogen, Carlsbad, CA, USA). RNA was quantified by the Quawell Q5000 spectrophotometer (NanoDrop Technologies, Wilmington, DE, USA). Reverse transcription was performed with the PrimeScript RT-PCR kit (#RR014, TaKaRa, Tokyo, Japan). RT-PCR was conducted using SYBR Green PCR Master Mix (#HY-K0501, MCE, Shanghai, China) and Quant Studio3 Real-Time PCR System (ABI, Austin, TX, USA). The quantitative data were calculated with the 2^−ΔΔ^Ct method and normalized to *ACTIN*. RT-PCR analysis was performed for *FASN, ACC, ACLY* and *ACTIN*. All the primers (Table 1) were produced by SunYa Biotechnology Co., LTD (Zhejiang, China).

**Table 1:** Primers sequences for qRT-PCR.

Gene Name	Primer sequences (5’-3’)
SREBP1-F	CTTAGAGCGAGCACTGAACTG
SREBP1-R	GGAACTGATGGAGAAGCTGTAG
FASN-F	CTCAGCCGCCATCTACAACA
FASN-R	GCCAGCGTCTTCCACACTAT
ACLY-F	AACAACCCAGACATGCGAGT
ACLY-R	AATGCGACTCCGATGAGACC
ACC-F	AGACTGTGGTGGTTGGTAGA
ACC-R	CTGCTGGATTATCTTGGCTTCA
ACTIN-F	GAGAAAATCTGGCACCACACC
ACTIN-R	GGATAGCACAGCCTGGATAGCAA

### Western blot

Cells were rinsed with ice-cold PBS for three times and lysed with RIPA lysis buffer (#9806, Cell Signaling Technology) containing 20 mM Tris-HCl (pH 7.5), 150 mM NaCl, 1 mM Na_2_EDTA, 1 mM EGTA, 1% NP-40, 1% sodium deoxycholate, 2.5 mM sodium pyrophosphate, 1 mM β-glycerophosphate, 1 mM Na_3_VO_4_, 1 µg/mL leupeptin and supplemented with 1 × protease/phosphatase inhibitor cocktail (#5872, Cell Signaling Technology) on ice for 30 min. The cell lysates were centrifuged at 15, 000 rpm in a microcentrifuge at 4°C for 15 minutes. The protein concentrations of the supernatants were determined using BCA protein assay reagent. 35 μg of protein lysates were loaded and separated in a 6%-12% SDS-PAGE gel, and then was transferred onto the polyvinylidene difluoride membrane. Membranes were blocked with 5% non-fat milk and incubated with primary antibodies (1:1,000) overnight at 4°C following incubated with HRP-conjugated secondary antibodies at room temperature for 1 h. Immunoreactive proteins were visualized by HRP-ECL chemiluminescence reagents and exposure to X-ray film to produce bands.

### Immunofluorescence

Cells were treated with indicated drugs for 48 h. Then the cells fixed with 4% paraformaldehyde for 30 min, permeabilized with 0.5% Triton X-100 for 15 min, and blocked in 5% goat serum albumin in PBS for 60 min. Then the cells were incubated with anti-SREBP1 antibody at 4 °C overnight. After PBS rinses, signals were detected with Anti-mouse IgG (Alexa Fluor^®^ 488 Conjugate) (#4408, CST) at room temperature for 60 min in dark, followed by DAPI (#4083, CST) staining at room temperature for 5 min. The slides were photographed under a fluorescence microscope.

### Raptor knockdown and Raptor over-expression by virus transfection

Raptor was knockdown by shRNA as previously described [23]. The target sequence is: *RAPTOR* 4145 sense, CCGGAGGGCCCTGCTACTCGCTTTTCTCGAGAAAAGCGAGTAGCAGGGCCCTTTTTTG; *RAPTOR* 4145 antisense, AATTCAAAAAAGGGCCCTGCTACTCGCTTTTCTCGAGAAAAGCGAGTAGCAGGGCCCT. The number indicate the nucleotide position in the transcripts (with position 1 set at the start codon) at which the 21 bp stem of the shRNA begins. The knockdown efficiency of the sequence has been verified in our another study [19]. shRNA directed against *RAPTOR* was constructed in GV248 vectors (Raptor-shRNA) and eukaryotic *RAPTOR* cDNA was constructed in GV358 vectors (GV358-Raptor) by Genechem Co., Ltd., (Shanghai, China). The production of lentiviruses for SKOV3 transfection was achieved by transfection of viral HEK-293T cells with Raptor-shRNA or GV358-Raptor constructs, with the VSV-G envelope and gag/pol packaging plasmids (Genechem Co., Ltd., Shanghai, China). Twelve hours after transfection, the media was changed to DMEM with 10% FBS. After another 48 hours, the virus-containing supernatant was collected from the cells and passed through a 0.45 μm filter following centrifugation at 4°C.

For Raptor knockdown, SKOV3 cells were infected with lentivirus containing media for 24 h in the presence of 8 μg/mL Polybrene. For Raptor overexpression, after lentivirus transfection for 16 h in the presence of 8 μg/mL Polybrene, the media was replaced with normal media and the cells were further cultured for 72 h. Then the culture media was replaced with selection media containing puromycin. The selection media was changed every 3 or 4 days until the puromycin resistant colonies appeared. The colonies were then individually picked out and cultured in the puromycin-containing medium. Raptor knockdown and overexpression was confirmed with Western blot analysis.

### Xenograft experiments

6-week old female BALB/c nude mice were purchased from Beijing HFK bioscience Co., Ltd and housed in the specific pathogen-free conditions. 1 × 10^6^ SKOV3 cells were suspended in the mixture of Matrigel (#356234, BD Biosciences) and PBS at a 1:1 ratio; and then subcutaneously injected into the right flank. Tumor growth was continuously monitored by calculating the tumor volume using the following formula: tumor volume (mm^3^) = 1/2 × length × width^2^. When the volume of the tumor reached 100 mm^3^, the tumor-bearing mice were randomly divided into three groups: control group (0.5% sodium carboxymethyl cellulose) and cardamonin (15 and 30 mg/kg) treatment groups. Intragastric administration of cardamonin was preformed once per days for 20 days. At the end of the treatment, the mice were euthanized by carbon dioxide inhalation. Specific criteria for humane endpoints for euthanasia were: if tumor burden of more than 10% of body mass or lost more than 20% of body weight; if we found an abnormal posture or lethargy/reluctance to move if stimulated or/and lack of movement; if we observed signs of suffering or anxiety such as isolation or withdrawn from other mice and expressed as semi-closed eyes and nose bulge; if we mentioned abnormal behavior like stereotypic movements and scratching the tumor until bleeding. Tumors were isolated and stained with immunohistochemistry. The animal experiments were approved by the Ethics Committee of Fujian Maternity and Child Health Hospital (Ethical approval No.: 2020KY088). We confirmed that the study was carried out in compliance with the ARRIVE guidelines.

### Immunohistochemistry

The isolated tumors were fixed with 4% paraformaldehyde and then encapsulated in paraffin. For immunohistochemistry, the encapsulated tissue was sliced into 4 μm slices. The slides were immersed in 10mM EDTA Tris-HCl antigen retrieval buffer and boiled for 15 minutes. The tumor sections were stained with antibodies against Ki-67 (1:1,000), Raptor (1:100), and FASN (1:800) in accordance with the instructions of the immunohistochemistry Kit.

### Statistical analysis

All data were expressed as the mean ± standard deviation (mean ± SD). Statistical analysis was performed using SPSS 21.0 statistical software. Difference between two groups was performed by t-test and differences of multiple groups were determined by one-way ANOVA followed by Tukey-Kramer test for post hoc comparisons. P < 0.05 was considered significant.

## Results

### Cardamonin inhibits cell viability and colony formation

The inhibitory efficiency of cardamonin was evaluated using CCK-8 assay. The IC50 of cardamonin on SKOV3 and A2780 cells were 23.4 μM and 28.8 μM (S1 Fig). Based on the result, we selected 10 μM and 30 μM cardamonin in the following experiments. Both concentrations of cardamonin significantly suppressed the cell viability of SKOV3 and A2780 cells (Fig 1B and 1C), consistent with our previous findings [24]. To further evaluate the anti-proliferative effect of cardamonin, colony formation assay was performed. The results revealed a significant reduction in the number of colonies of cardamonin-treated SKOV3 and A2780 cells compared to untreated control cells (Fig 1D and 1E). It suggests that cardamonin effectively suppresses the cell proliferation of ovarian cancer cells.

### Cardamonin induces apoptosis and reduces MMP

**Fig 2:**
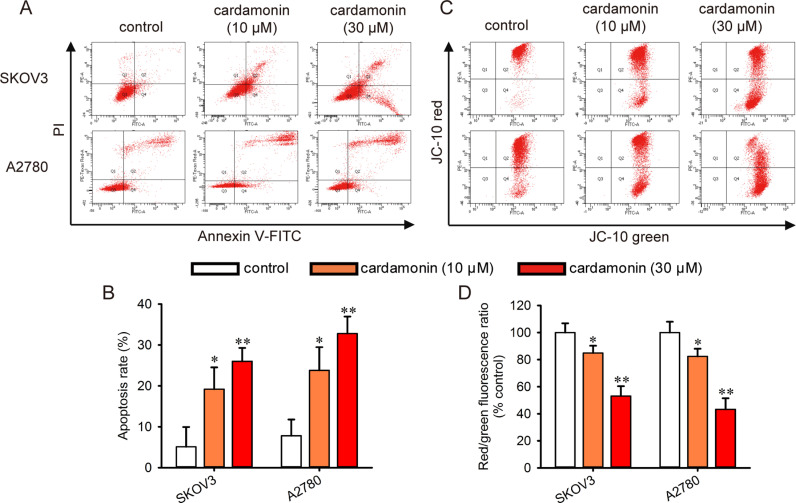
Cardamonin induced apoptosis and decreased the MMP of ovarian cancer cells. Cell apoptosis and MMP of SKOV3 and A2780 cells was determined by flow cytometry. Both cells were treated with indicated drugs for 48 h. Then the cells were stained with Annexin V-FITC and PI (*n = 3*). (A) Representative flow cytometry scatter plots depict the percentage of Annexin V and PI staining. (B) The histogram showed the percentages of apoptosis cells. Changes in MMP were determined by JC-10 staining assay (*n = 3*). (C) Representative flow cytometry scatter plots depict percentage of JC-10 staining. (D) The diagram showed the quantitative ratio of JC-10 aggregates (red fluorescence) to JC-10 monomers (green fluorescence). The ratio was normalized to control. Control group presented ovarian cancer cells without any treatment. All the data were expressed as means ± SD. ^*^P < 0.05, ^**^P < 0.01 compared with control.

Flow cytometry assay revealed that cardamonin significantly increased the apoptotic cell population compared to that in control cells (Fig 2A and 2B). Then the MMP was assessed using JC-10 staining. Cardamonin treatment (30 μM) reduced MMP to 51.7% and 44.8% in SKOV3 and A2780 cells, respectively (Fig 2C and 2D). It indicates that cardamonin induces mitochondrial damage in ovarian cancer cells.

### Cardamonin decreases DNL and CPT-1 activity

**Fig 3:**
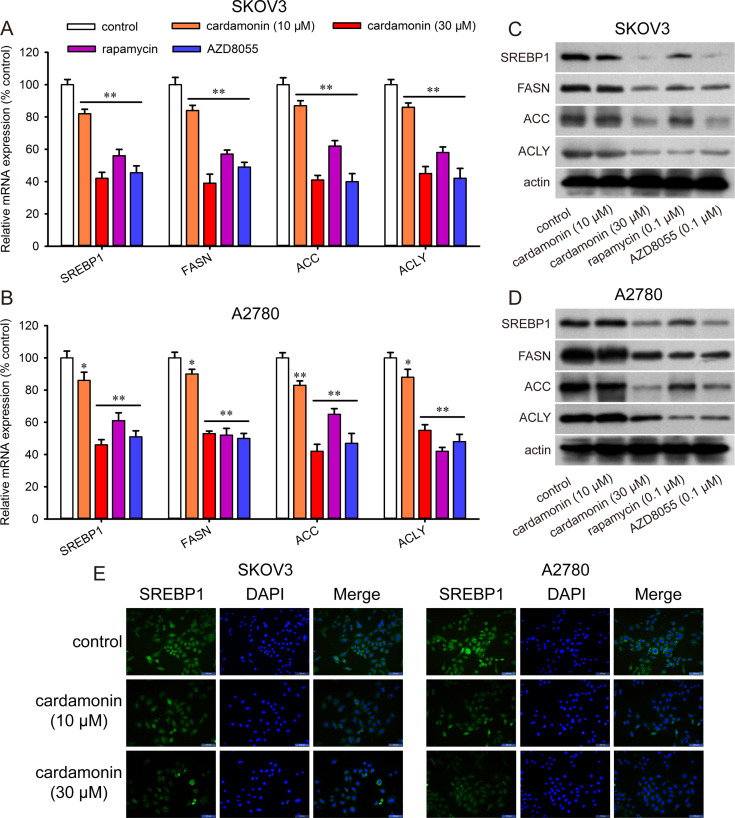
Cardamonin decreased the expression of lipogenic proteins. SKOV3 and A2780 ovarian cancer cells were treated with cardamonin, rapamycin and AZD8055 for 48 h, respectively. The mRNA expression of *SREBP1, FASN, ACC, ACLY* were measured by qRT-PCR (*n = 3*). (A, B) The relative mRNA expression of SKOV3 and A2780 cells were quantified. All the data were expressed as means ± SD. ^*^P < 0.05, ^**^P < 0.01 compared with control. (C, D) The protein expression of SREBP1, FASN, ACC, ACLY were measured by Western blot in SKOV3 and A2780 cells. (E) The localization of SREBP1 in SKOV3 and A2780 cells was determined by immunofluorescence. Control group presented ovarian cancer cells without any treatment.

DNL plays a critical role in regulating the survival and proliferation of cancer cell. Here, we investigated whether cardamonin inhibited lipogenesis in ovarian cancer cells. Cardamonin significantly decreased the mRNA (Fig 3A and 3B) and protein (Fig 3C and 3D) expression of SREBP1, FASN, ACC, and ACLY in both SKOV3 and A2780 cells (S2 Fig). SREBP1 is a nuclear transcription factor involved in lipid synthesis. we determined the effect of cardamonin on the localization of SREBP1. In control cells, SREBP1 was localized to the cytoplasm and nucleus; and its expression decreased upon cardamonin treatment. And the immunoreactivity of SREBP1 was weaker in the nucleus (Fig 3E).

**Fig 4:**
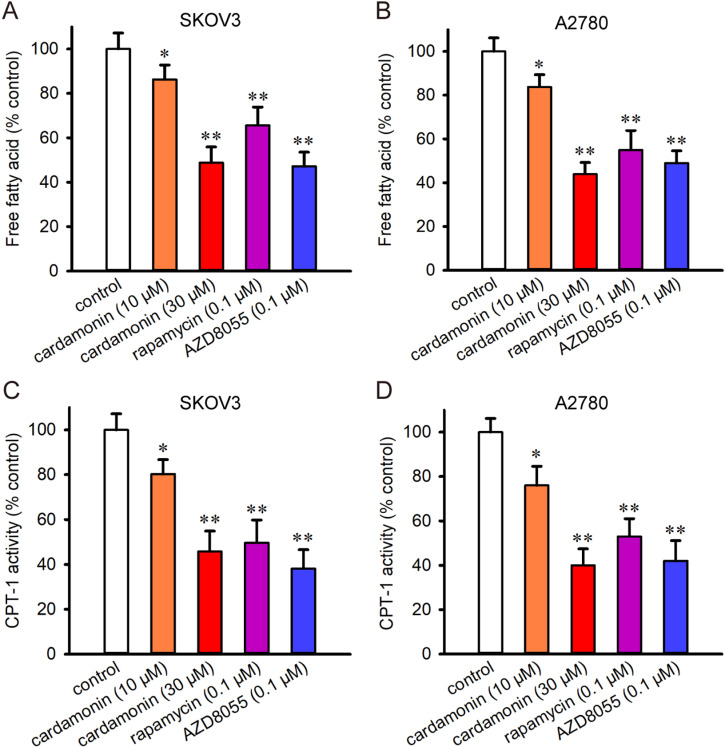
Cardamonin decreased the content of free fatty acids and the activity of CPT-1. SKOV3 and A2780 cells were treated with cardamonin, rapamycin and AZD8055 for 48 h, respectively. The content of free fatty acid and the activity of CPT-1 were determined by spectrophotometry (*n = 3*). (A, B) The percentage of free fatty acid levels of SKOV3 and A2780 cells compared with the control. (C, D) The percentages of the CPT-1 activity of SKOV3 and A2780 cells compared with the control. Control group presented ovarian cancer cells without any treatment. All the data were expressed as means ± SD. ^*^P < 0.05, ^**^P < 0.01 compared with control.

This reduction in lipogenic protein expression was further confirmed by the free fatty acid assay, which revealed that cardamonin decreased in the levels of cellular lipids (Fig 4A and 4B). FASN inhibition facilitates apoptosis by decreasing the activity of CPT-1 [25]. Therefore, we investigated the effect of cardamonin on CPT-1 activity. As shown in Fig 4C and 4D, cardamonin decreased CPT-1 activity in SKOV3 and A2780 cells, respectively. These findings indicate that cardamonin inhibits lipogenesis, reduces CPT-1 activity and induces apoptosis in ovarian cancer cells.

### Cardamonin inhibits the mTORC1 signaling pathway

**Fig 5:**
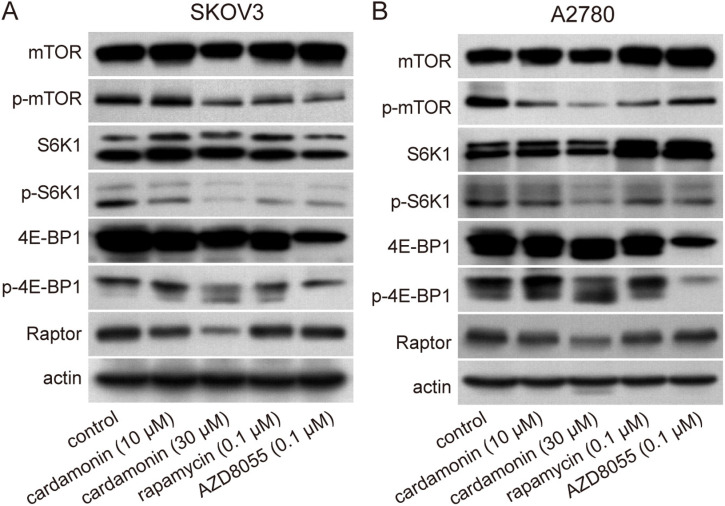
Cardamonin inhibited the activation of mTORC1 and protein expression of Raptor. SKOV3 and A2780 ovarian cancer cells were treated with cardamonin, rapamycin and AZD8055 for 48 h, respectively. (A, B) The phosphorylation and protein expression of mTOR, S6K1, 4E-BP1 and Raptor were detected by Western blot. Control group presented ovarian cancer cells without any treatment. Actin was used as an equal loading control.

As mTORC1 activation results in increased DNL, we investigated the inhibitory effect of cardamonin on the mTORC1 signaling pathway. Both cardamonin and the mTOR inhibitors, rapamycin (an mTORC1 inhibitor) and AZD8055 (an mTORC1 and mTORC2 dual inhibitor), effectively inhibited the phosphorylation of mTOR, S6K1, and 4E-BP1. Notably, AZD8055 exhibited higher sensitivity in inhibiting 4E-BP1 phosphorylation than cardamonin and rapamycin. Consistent with our previous studies, cardamonin specifically decreased the protein expression of Raptor, whereas rapamycin and AZD8055 had no effect (Fig 5 and S3 Fig). Our findings suggest that cardamonin acts as a potential mTORC1 inhibitor with a mechanism distinct from those of rapamycin and AZD8055.

### Raptor knockdown inhibits cell viability and SREBP1

**Fig 6:**
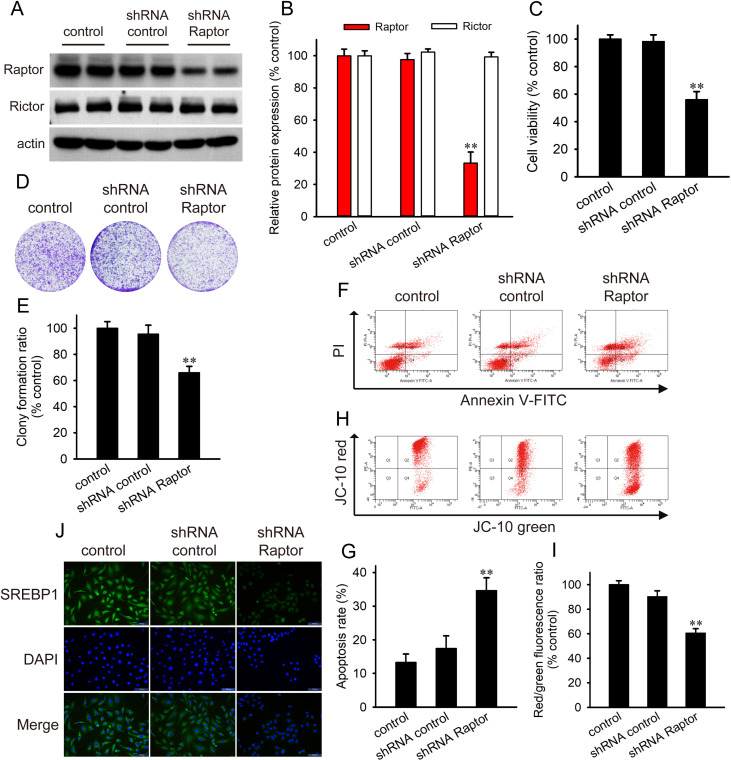
Raptor knockdown inhibited SKOV3 cells. (A, B) Raptor was knockdown using shRNA in SKOV3. (C, D, E) The effect of shRNA Raptor on the cell viability of SKOV3 cells were detected by CCK-8 assay and colony formation assay. (F, G, H, I) Cell apoptosis and MMP of SKOV3 cells was determined by flow cytometry. (J) The localization of SREBP1 was determined by immunofluorescence. Control group presented SKOV3 cells without any treatment. shRNA control presented SKOV3 cell that were transfected empty vector. All the data were expressed as means ± SD. ^**^P < 0.01 compared with control.

The results above demonstrated that cardamonin decreased the protein expression of Raptor and lipogenesis in ovarian cancer cells. We hypothesized that Raptor mediated the inhibitory effect of cardamonin on lipogenesis. Then Raptor was knocked down in SKOV3 cells using shRNA (Fig 6A and 6B). As expected, Raptor knockdown decreased the cell viability and proliferation (Fig 6C–6E). Cell apoptosis increased and MMP decreased upon shRNA Raptor transfection (Fig 6F–6I). Similarly, immunofluorescence analysis demonstrated that the nuclear translocation of SREBP1 was repressed in Raptor-knockdown cells (Fig 6J).

### Cardamonin decreases Raptor and suppresses lipogenesis

**Fig 7:**
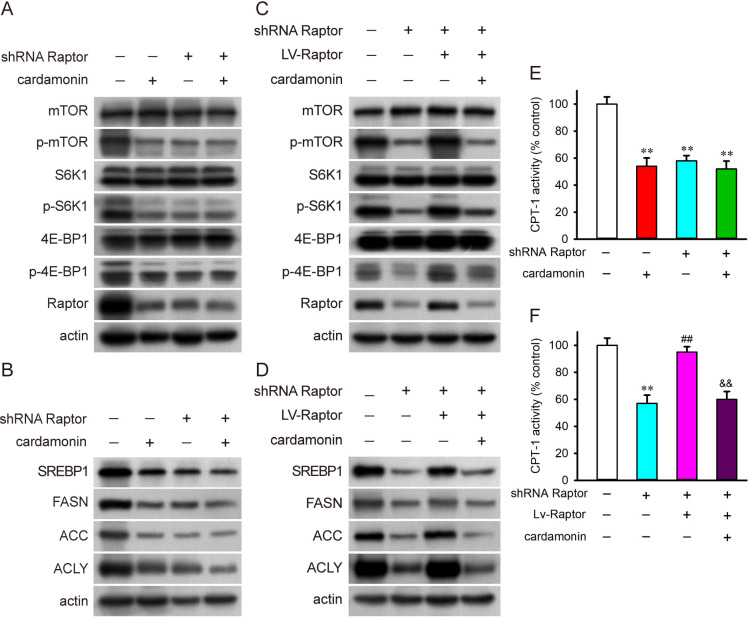
Cardamonin decreased the protein level of Raptor and suppressed lipogenesis and CPT-1. Normal and Raptor-knockdown SKOV3 cells were treated with cardamonin (30 μM). (A) The phosphorylation and protein expression of mTOR, S6K1, 4E-BP1 and (B) the protein expression of SREBP1, FASN, ACC, ACLY were detected by Western blot. Raptor was overexpressed by lentivirus transfection in the Raptor-knockdown cells. The transfected cells were treated with cardamonin (30 μM). (C) The phosphorylation and protein expression of mTOR, S6K1, 4E-BP1 and (D) the protein expression of SREBP1, FASN, ACC, ACLY were detected by Western blot. (E, F) The activity of CPT-1 was determined by spectrophotometry (*n = 3*). The percentages of the CPT-1 activity was compared with the control. All the data were expressed as means ± SD. ^**^P < 0.01 compared with control; ^##^*P* < 0.01 compared with shRNA Raptor; ^&&^*P* < 0.01 compared with shRNA Raptor + Lv-Raptor.

In Raptor-knockdown SKOV3 cells, we confirmed that Raptor depletion phenocopied the inhibitory effects of cardamonin on mTORC1 signaling. Moreover, cardamonin had no additional inhibitory effect on mTORC1 in Raptor-knockdown cells (Fig 7A and S4A Fig). Consistent with mTORC1 inhibition, Raptor knockdown significantly decreased the protein expression of SREBP1, FASN, ACC, and ACLY, which were not further decrease by cardamonin (Fig 7B and S4B Fig).

Next, we conducted Lv-Raptor transfection to overexpress Raptor in the Raptor-knockdown SKOV3 cells. Raptor overexpression rescued the depleted mTORC1 activation and restored the expression of SREBP1, FASN, ACC, and ACLY. As expected, cardamonin abrogated the mTOR activation and SREBP1 expression induced by Raptor overexpression (Fig 7C and 7D; S4C and S4D Figs). Furthermore, Raptor knockdown abolished the inhibitory effect of cardamonin on the activity of CPT-1 (Fig 7E). In addition, cardamonin inhibited CPT-1 which restored by Raptor overexpression (Fig 7F).

### Cardamonin inhibits tumor growth and decreases lipogenesis

**Fig 8:**
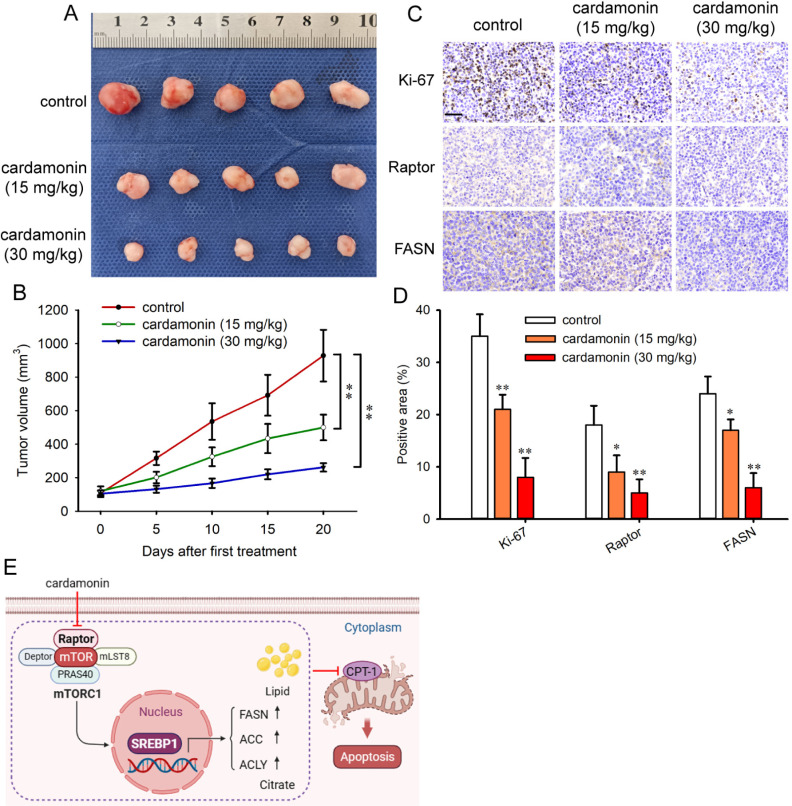
Cardamonin inhibited tumor growth and decreased FASN and Raptor. (A) Tumor images of subcutaneous xenografts in nude mice implanted with SKOV3 cells and treated with different dosage of cardamonin. (B) Xenograft tumor volume was monitored and quantified. (C) Immunohistochemistry staining of Ki-67, Raptor and FASN in the xenograft tumor tissues. Scale bar: 40 μm. (D) The semiquantification of Ki-67, Raptor and FASN in the tumor tissues (*n = 5*). ^*^P < 0.05, ^**^P < 0.01 compared with control. (E) The mechanism of cardamonin in inhibiting DNL and inducing apoptosis in ovarian cancer. Cardamonin reduces the protein expression of Raptor, thereby inhibiting the mTORC1-regulated DNL pathway. This leads to a decrease in free fatty acid levels. Subsequently, the reduced levels of free fatty acids inhibit the activity of mitochondrial CPT-1 and induces mitochondrial dependent apoptosis. The mechanism of cardamonin on the inhibition of DNL and induction of apoptosis in ovarian cancer. Cardamonin decreases the protein expression of Raptor and inhibits mTORC1 regulated DNL pathway, which results in reduction of free fatty acid levels. Subsequently, decreased free fatty acid inhibits the activity of mitochondrial CPT-1 and disrupts lipid β-oxidation. This inhibition induces mitochondrial dependent cell apoptosis. Schematic made with BioRender.

The anti-tumor effect of cardamonin was investigated using the SKOV3 cell xenograft tumor model. Cardamonin significantly reduced tumor growth in nude mice bearing tumors (Fig 8A and 8B). The antiproliferative effect of cardamonin on tumors was also evaluated. Consistent with the in vitro results, cardamonin inhibited cell proliferation ability, as evidenced by reduced Ki-67 expression. In addition, immunohistochemistry revealed that cardamonin decreased the expression of Raptor and FASN (Fig 8C and 8D). Collectively, these findings suggest the association of decreased lipogenesis and mTORC1 with inhibited tumor growth (Fig 8E).

## Discussion

Cardamonin exhibits promising potential in the treatment of cancer. It inhibits cell proliferation and increases apoptosis, cycle arrest via various signaling pathways like the NF-κB, Wnt/β-catenin and mTOR, which are crucial for cancer progression and development [26].

As a core regulator of cellular metabolism, mTOR modulates the reprogram of glucose and lipid metabolism, which provides nutrition for the rapid proliferation and survival of cancer cells [27]. It has been demonstrated that cardamonin reduces glucose uptake as well as lactate secretion and efflux, suggesting its function in repressing the glycolysis process [28]. However, the effect of cardamonin on the lipid metabolism of cancer cells is not investigated.

Growing evidence has highlighted the significant role of lipogenesis and fatty acid oxidation in the progression of ovarian cancer, including metastasis, stemness and chemotherapy resistance. In cells with elevated mTORC1 levels, rapamycin-sensitive lipogenic genes are enriched in the SREBPs binding region [29]. Whereas, in mice with adipose-specific mTORC1 inhibition, the function of SREBP1 is abrogated, leading to decreased hepatic steatosis and hypercholesterolemia induced by a high-fat diet [30]. As expected, cardamonin decreased the level of free fatty acids and the protein expression of SREBP1, FASN, ACC, and ACLY, indicating that cardamonin inhibited DNL in ovarian cancer cells. In addition, we found that the expression of lipogenic proteins was decreased in Raptor-knockdown SKOV3 cells. Whereas, Raptor overexpressing restored the decreased lipogenic proteins, which were further decreased by cardamonin. These results suggest that the inhibition of lipogenesis by cardamonin is associated with Raptor. However, the role of Raptor in adipose tissue remains unclear. Kim *et al.* found that, although mTORC1 promotes lipogenesis through SREBP1, the mTORC1-independent Raptor reduces adipogenesis [31]. Furthermore, Lee *et al.* demonstrated that Ad-RapKO mice displayed hepatomegaly and hepatic steatosis [32]. It indicates that Raptor may have a dual-directional regulation of hepatic lipid accumulation. Although cardamonin stimulates adipocyte browning, suppresses lipogenesis, and promotes lipolysis in 3T3-L1 adipocytes, implying it may have potential as an anti-obesity agent [21]. Further studies are needed to confirm whether cardamonin can be used as a potential treatment for obesity.

mTORC1 also regulates the transcriptional activity of SREBP1 at the trafficking level. SREBP1 is normal embedded in the endoplasmic reticulum membrane as an inactive precursor. In response to lipid starvation, SREBP1 translocates to the nucleus to induce the transcription of lipogenic genes [33]. Activation of mTORC1 induces SREBP1 nuclear accumulation and promotes DNL [29]; whereas rapamycin blocks the nuclear accumulation of SREBP1 and decreases the expression of lipogenic genes [34]. In this study, we demonstrated that cardamonin decreased the protein expression and nuclear localization of SREBP1. However, the effect of cardamonin on the nuclear translocation of SREBP1 has not been demonstrated. Peterson *et al.* demonstrates that mTORC1 regulated SREBP1 through controlling the nuclear entry of Lipin1 [33]. In 3T3-L1 adipocytes, cardamonin reduces the protein expression of Lipin1 [21]. Whether cardamonin regulates the translocation of SREBP1 through Lipin1 should be further investigated.

Cardamonin induces apoptotic action through the activation of both extrinsic and intrinsic pathways [35]. Previous studies have demonstrated that cardamonin increases cleaved Caspases levels, intracellular reactive oxygen species (ROS) and specifically alters the mitochondrial membrane potential (MMP). FASN inhibition has been linked to intrinsic cell apoptosis through increased ROS and the activation of Caspase-9 and Caspase-3 [36]. Therefore, we propose that the reduction in fatty acid synthesis may contribute to the apoptosis-inducing effects of cardamonin. Mitochondrial β-oxidation is an important step in lipid metabolism. CPT-1, which is localized to the outer mitochondrial membrane, translocates long-chain fatty acids across the mitochondrial membranes and catalyzes the rate-limiting step of β-oxidation [37]. FASN inhibition increases malonyl CoA levels, which suppresses fatty acids β-oxidation of by inhibiting the activity of CPT-1 [38]. CPT-1 inhibition causes mitochondrial damage by activating apoptotic proteins [25]. In this study, the expression of FASN was reduced by cardamonin as well as the CPT-1 activity and MMP; meanwhile the cell apoptosis was increased. We speculate that cardamonin inhibits lipogenesis and β-oxidation, leading to mitochondrial damage and apoptosis.

Raptor plays a critical role in the activation of mTORC1 during tumorigenesis and cancer progression. It contributes to PI3K-mTOR inhibitor resistance in renal cancer [39]. Raptor knockdown effectively blocks the cell cycle and cell proliferation of ovarian cancer cells [40]. We demonstrate that cardamonin inhibits mTOR activation by splitting Raptor through caspases [41] and inhibits cell proliferation and activation of mTOR in mTOR inhibitor-resistant cells [42]. Therefore, Raptor may be considered as a drug target for cancer treatment. In this study, Raptor shRNA decreased the activity of mTOR, and cardamonin had no additional inhibitory effect on the phosphorylation of mTOR and S6K1 in the Raptor-knockdown cells. Raptor overexpression restored the activation of mTOR in the Raptor-knockdown cells. As expected, cardamonin inhibited the activation of mTOR induced by Raptor overexpression. These findings further indicate that cardamonin inhibits mTORC1 through decreasing the protein level of Raptor.

## Conclusions

In summary, these results indicate that cardamonin suppresses proliferation, induces apoptosis, and triggers mitochondrial damage in ovarian cancer cells. Raptor is associated with the inhibitory effect of cardamonin on DNL by suppressing mTOR/SREBP1.

## Supporting information

S1 Fig Cardamonin inhibited the proliferation of ovarian cancer cells. SKOV3 and A2780 were treated with 2.5, 5, 10, 20, 40, 80 μM of cardamonin for 48 h; cell viability was assessed the CCK-8 assay (*n = 3*). The inhibition rate and IC50 of cardamonin on SKOV3 and A2780 was calculated.
(TIF)

S2 Fig Cardamonin decreased the protein expression of lipogenic proteins. SKOV3 and A2780 ovarian cancer cells were treated with cardamonin, rapamycin and AZD8055 for 48 h, respectively. The protein expression of SREBP1, FASN, ACC, ACLY were measured by Western blot in SKOV3 and A2780 cells. The intensity of the protein bands of (A) SKOV3 and (B) A2780 cells were quantified (*n = 3*). The protein expression was normalized to control. Control group presented ovarian cancer cells without any treatment. All the data were expressed as means ± SD. ^*^P < 0.05, ^**^P < 0.01 compared with control.
(TIF)

S3 Fig Cardamonin inhibited the activation of mTORC1 and protein expression of Raptor. SKOV3 and A2780 ovarian cancer cells were treated with cardamonin, rapamycin and AZD8055 for 48 h, respectively. The phosphorylation and protein expression of mTOR, S6K1, 4E-BP1 and Raptor were detected by Western blot. Actin was used as an equal loading control (*n = 3*). The intensity of the protein bands of (A) SKOV3 and (B) A2780 cells were quantified. The protein expression was normalized to control. Control group presented ovarian cancer cells without any treatment. All the data were expressed as means ± SD. ^*^P < 0.05, ^**^P < 0.01 compared with control.
(TIF)

S4 Fig Raptor shRNA abolished the inhibitory effect of cardamonin on mTOR signaling and lipogenic proteins. The protein expression of Raptor was knocked down by shRNA in SKOV3 cells. Then, normal and Raptor-knockdown SKOV3 cells were treated with cardamonin (30 μM). (A) The phosphorylation and protein expression of mTOR, S6K1, 4E-BP1 were detected by Western blot. The intensity of the protein bands was quantified. (B) The protein expression of SREBP1, FASN, ACC, ACLY were detected by Western blot. The intensity of the protein bands was quantified. Actin was used as an equal loading control (*n = 3*). ^**^P < 0.01 compared with control. Cardamonin inhibited the Raptor overexpression induced mTORC1 activation and lipogenic proteins expression. The protein expression of Raptor was knocked down by shRNA in SKOV3 cells. Then, Raptor was overexpressed by lentivirus transfection in the Raptor-knockdown cells. The transfected cells were treated with cardamonin (30 μM). (C) The phosphorylation and protein expression of mTOR, S6K1, 4E-BP1 were detected by Western blot. The intensity of the protein bands was quantified. (D) The protein expression of SREBP1, FASN, ACC, ACLY were detected by Western blot. The intensity of the protein bands was quantified. Actin was used as an equal loading control (*n = 3*). Control group presented ovarian cancer cells without any treatment. ^**^P < 0.01 compared with control; ^##^*P *< 0.01 compared with shRNA Raptor; ^&&^*P* < 0.01 compared with shRNA Raptor + Lv-Raptor.
(TIF)

S1 File Original data. (ZIP)

S2 File Original western blotimages. (ZIP)
