# Supplementary figures and images for: Cardamonin suppresses mTORC1/SREBP1 through reducing Raptor and inhibits de novo lipogenesis in ovarian cancer
Source: PLoS One. 2025 May 2;20(5):e0322733. doi: 10.1371/journal.pone.0322733 (PMC12047825; doi:10.1371/journal.pone.0322733)

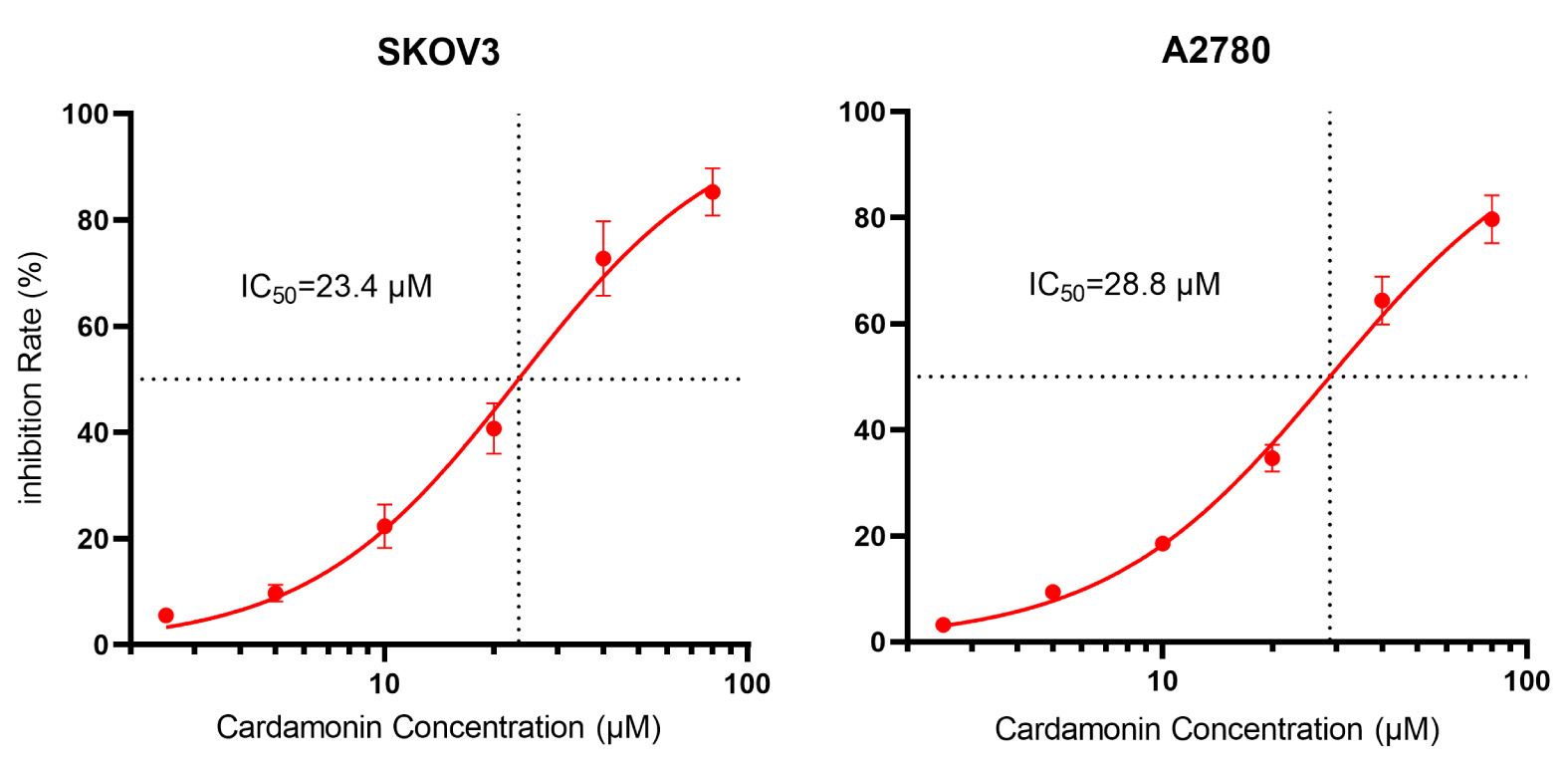

Supplement: S1 Fig — SKOV3 and A2780 were treated with 2.5, 5, 10, 20, 40, 80 μM of cardamonin for 48 h; cell viability was assessed the CCK-8 assay (n = 3). The inhibition rate and IC50 of cardamonin on SKOV3 and A2780 was calculated. (TIF) [file pone.0322733.s001.tif]

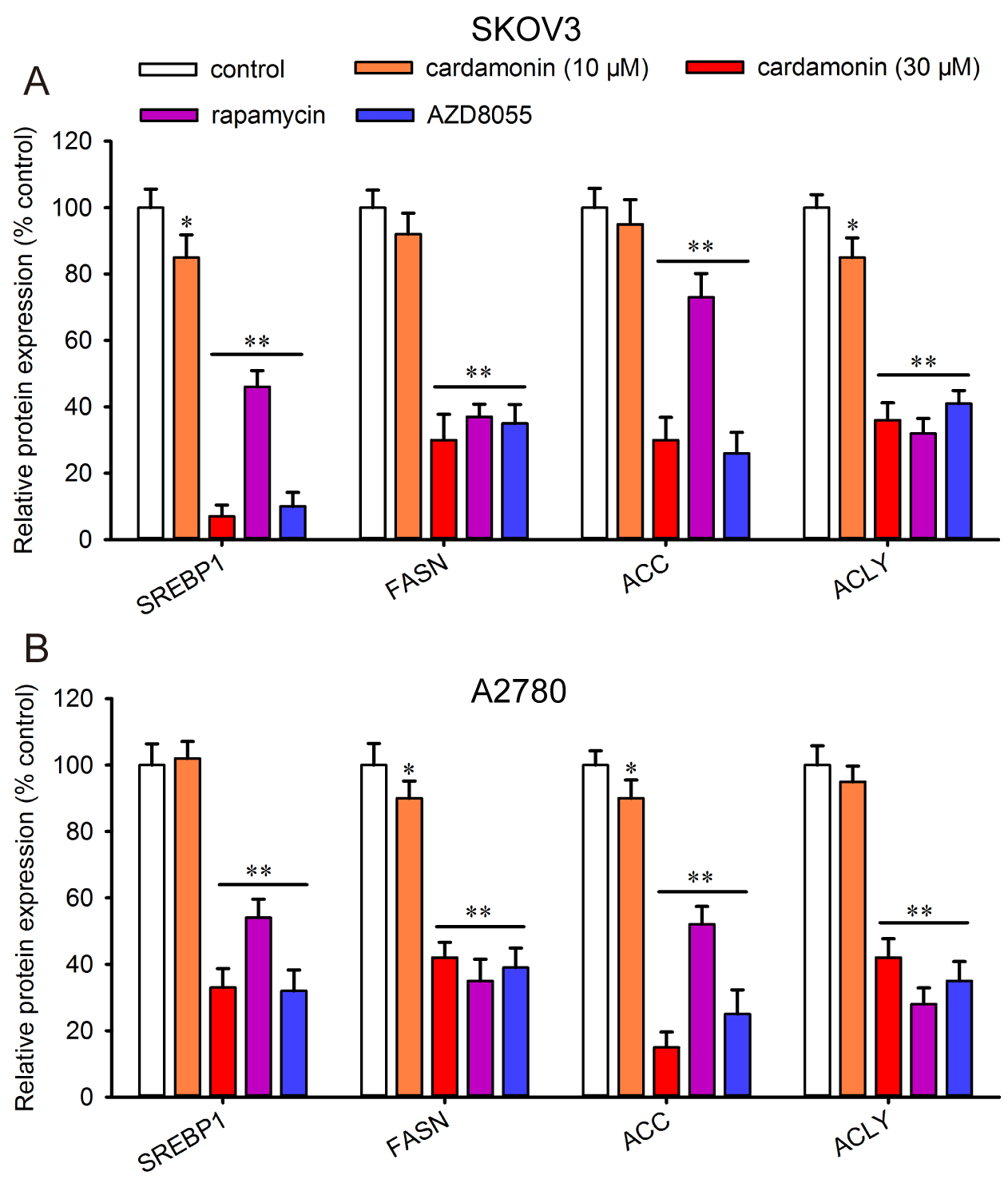

Supplement: S2 Fig — SKOV3 and A2780 ovarian cancer cells were treated with cardamonin, rapamycin and AZD8055 for 48 h, respectively. The protein expression of SREBP1, FASN, ACC, ACLY were measured by Western blot in SKOV3 and A2780 cells. The intensity of the protein bands of (A) SKOV3 and (B) A2780 cells were quantified (n = 3). The protein expression was normalized to control. Control group presented ovarian cancer cells without any treatment. All the data were expressed as means ± SD. *P < 0.05, **P < 0.01 compared with control. (TIF) [file pone.0322733.s002.tif]

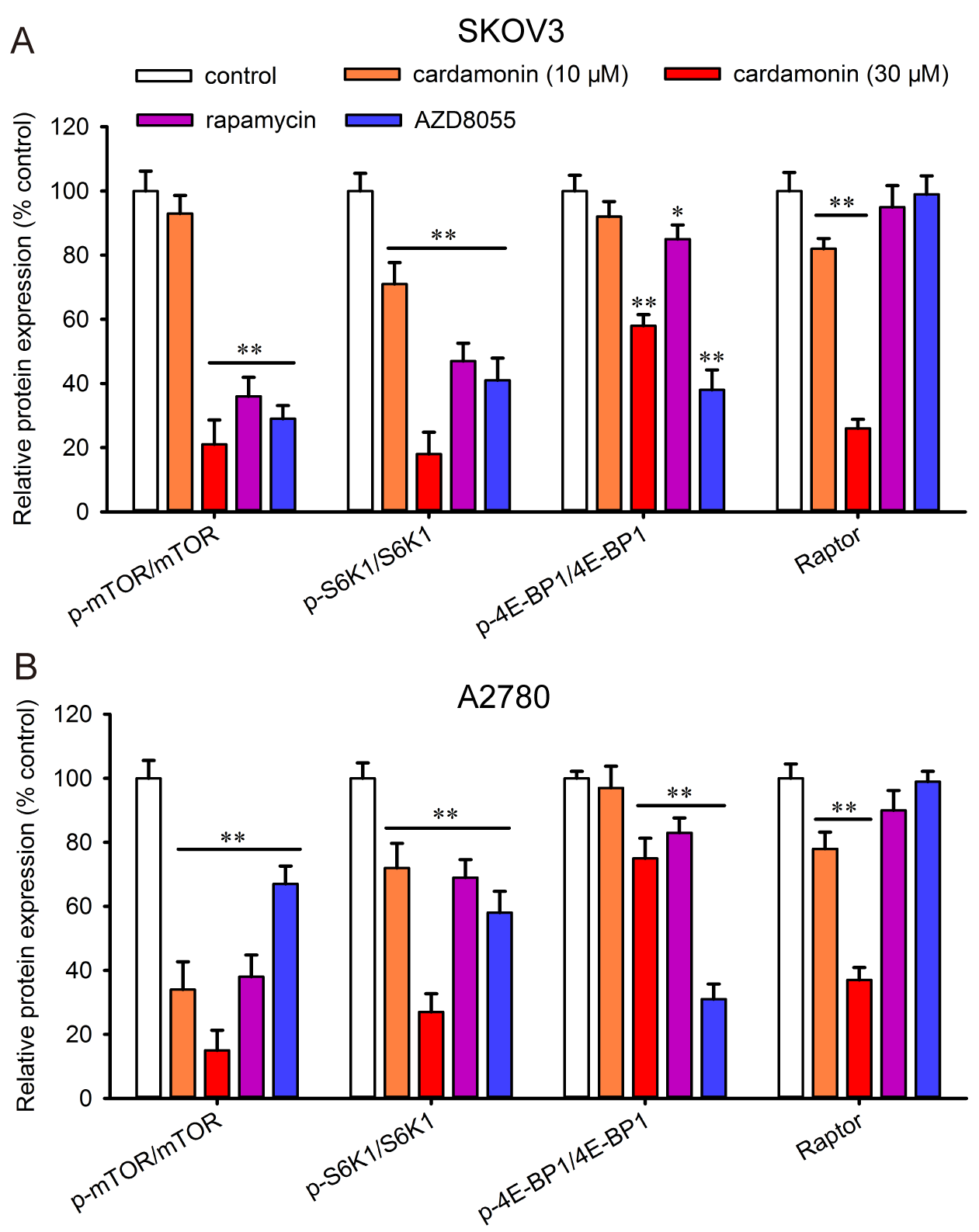

Supplement: S3 Fig — SKOV3 and A2780 ovarian cancer cells were treated with cardamonin, rapamycin and AZD8055 for 48 h, respectively. The phosphorylation and protein expression of mTOR, S6K1, 4E-BP1 and Raptor were detected by Western blot. Actin was used as an equal loading control (n = 3). The intensity of the protein bands of (A) SKOV3 and (B) A2780 cells were quantified. The protein expression was normalized to control. Control group presented ovarian cancer cells without any treatment. All the data were expressed as means ± SD. *P < 0.05, **P < 0.01 compared with control. (TIF) [file pone.0322733.s003.tif]

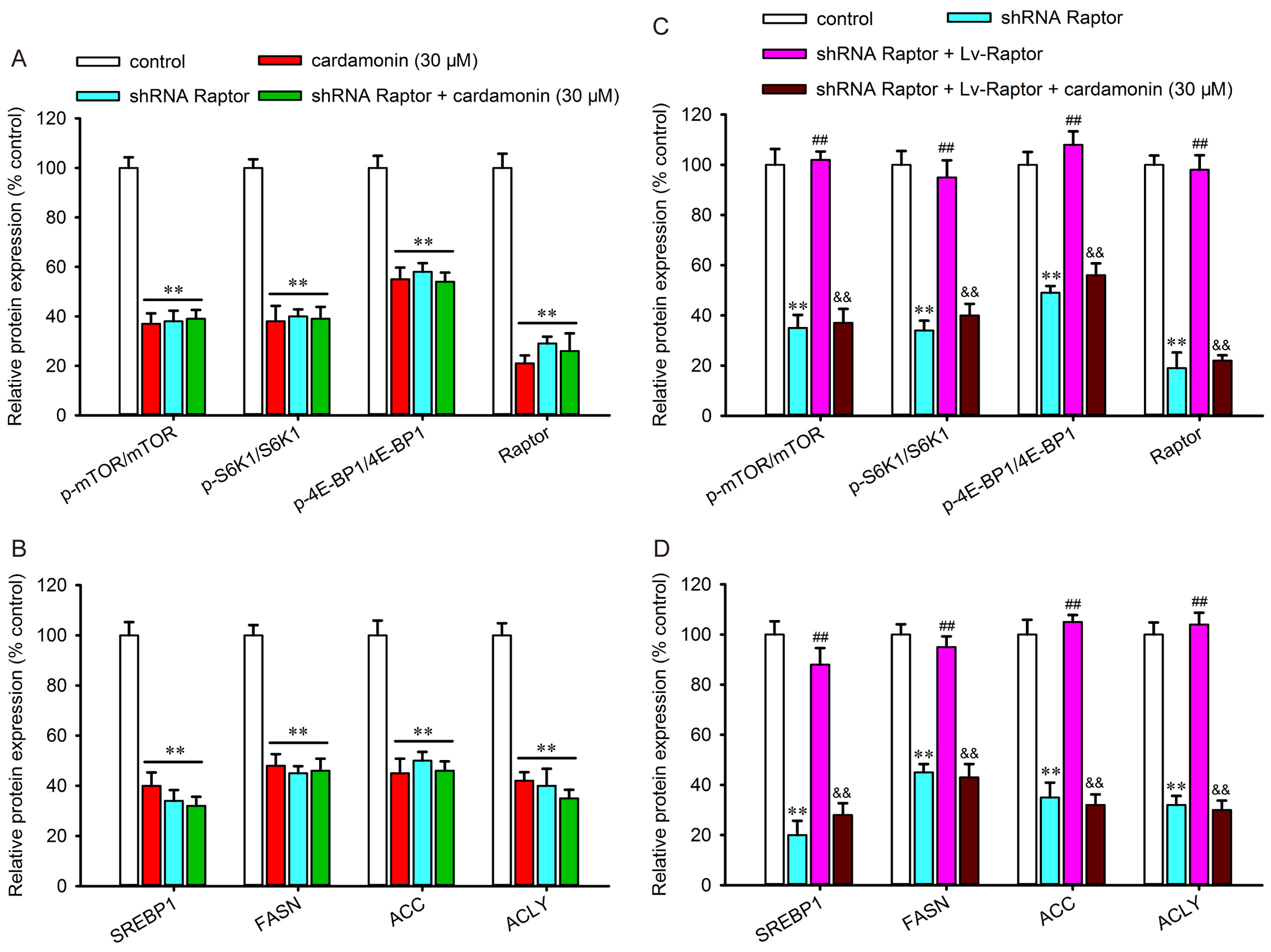

Supplement: S4 Fig — The protein expression of Raptor was knocked down by shRNA in SKOV3 cells. Then, normal and Raptor-knockdown SKOV3 cells were treated with cardamonin (30 μM). (A) The phosphorylation and protein expression of mTOR, S6K1, 4E-BP1 were detected by Western blot. The intensity of the protein bands was quantified. (B) The protein expression of SREBP1, FASN, ACC, ACLY were detected by Western blot. The intensity of the protein bands was quantified. Actin was used as an equal loading control (n = 3). **P < 0.01 compared with control. Cardamonin inhibited the Raptor overexpression induced mTORC1 activation and lipogenic proteins expression. The protein expression of Raptor was knocked down by shRNA in SKOV3 cells. Then, Raptor was overexpressed by lentivirus transfection in the Raptor-knockdown cells. The transfected cells were treated with cardamonin (30 μM). (C) The phosphorylation and protein expression of mTOR, S6K1, 4E-BP1 were detected by Western blot. The intensity of the protein bands was quantified. (D) The protein expression of SREBP1, FASN, ACC, ACLY were detected by Western blot. The intensity of the protein bands was quantified. Actin was used as an equal loading control (n = 3). Control group presented ovarian cancer cells without any treatment. **P < 0.01 compared with control; ##P < 0.01 compared with shRNA Raptor; &&P < 0.01 compared with shRNA Raptor + Lv-Raptor. (TIF) [file pone.0322733.s004.tif]

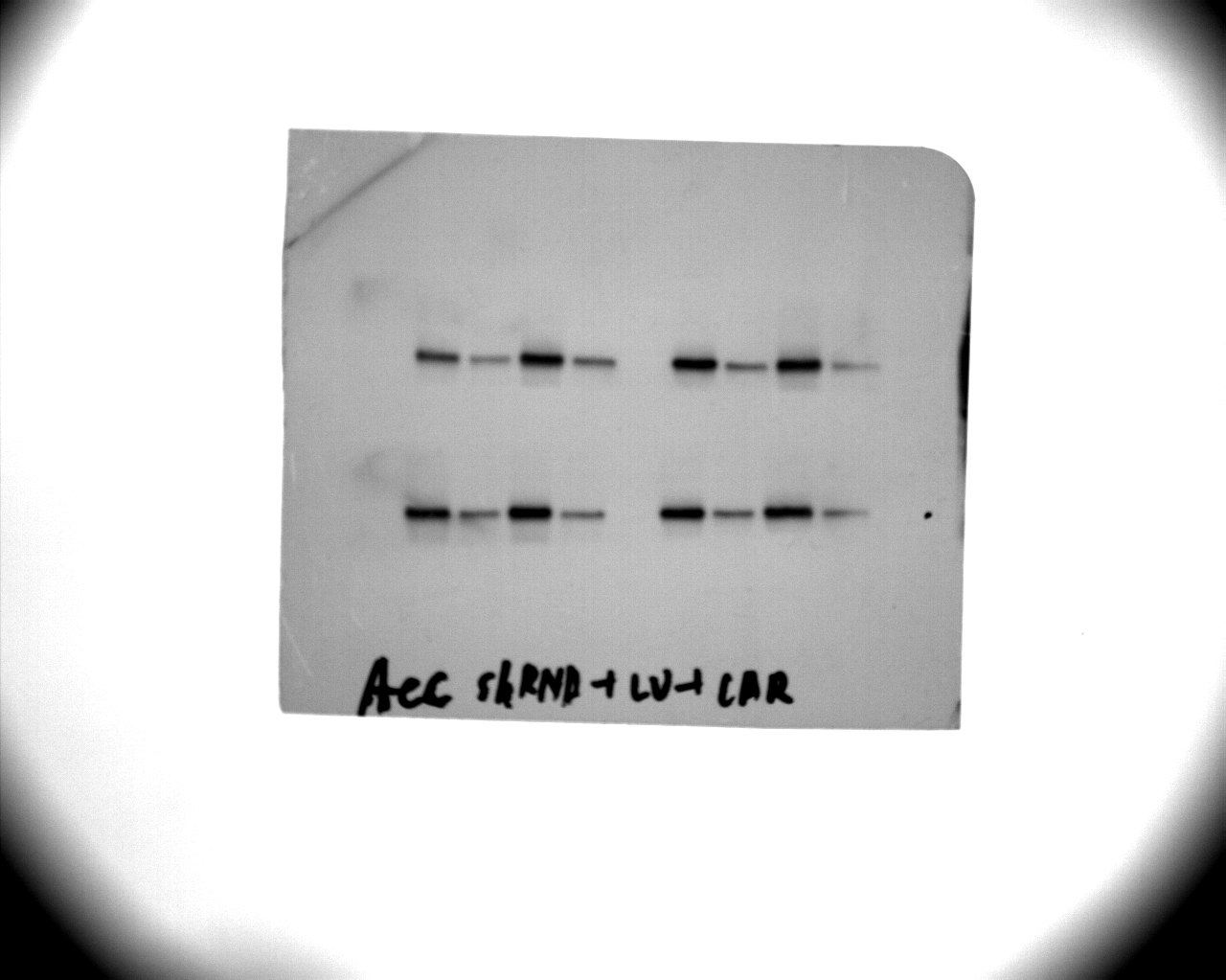

Supplement: S2 File — (ZIP) [file pone.0322733.s006.zip › Original Western Blot Images/Original Western Blot Images/Fig.7D/ACC.tif]

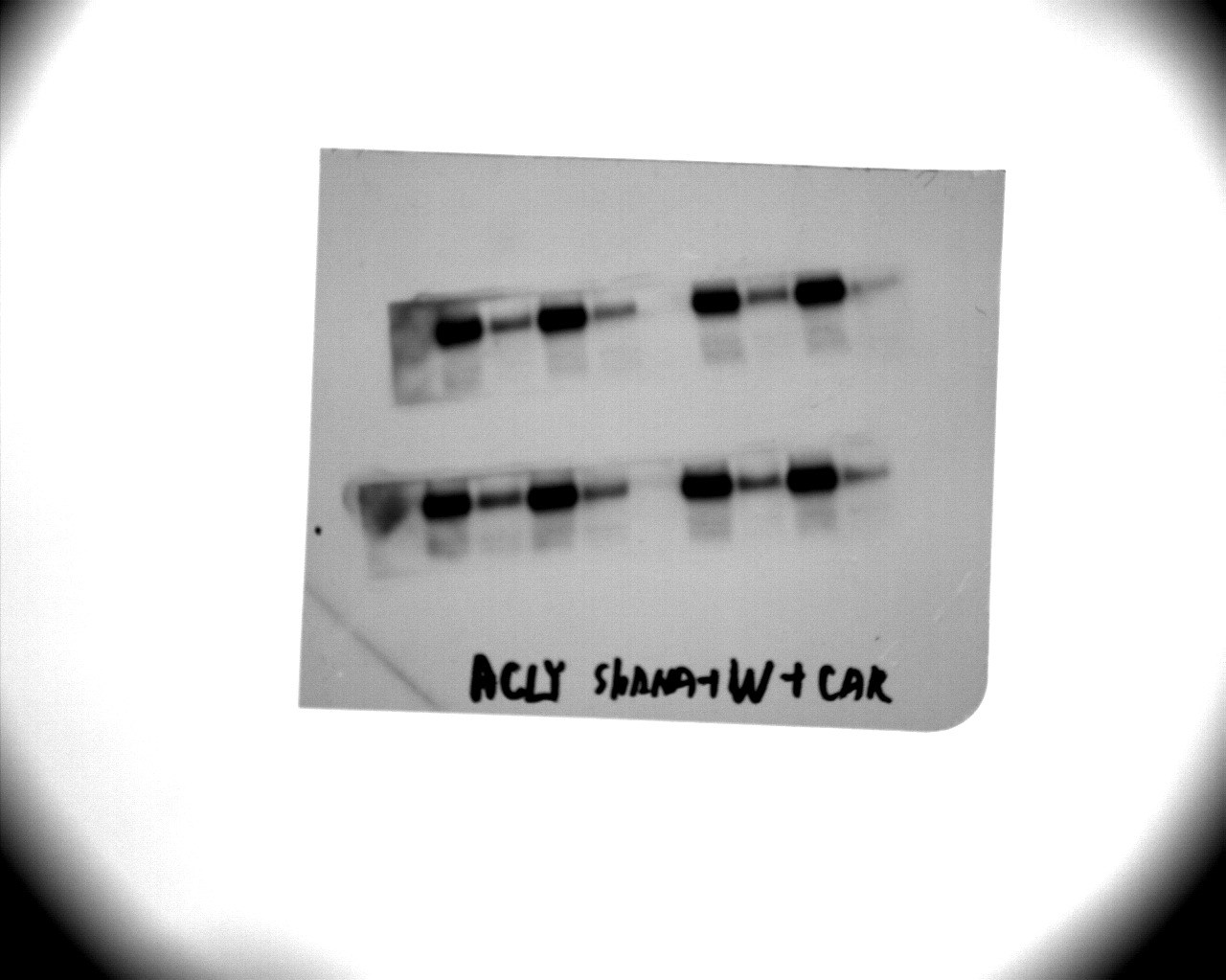

Supplement: S2 File — (ZIP) [file pone.0322733.s006.zip › Original Western Blot Images/Original Western Blot Images/Fig.7D/ACLY.tif]

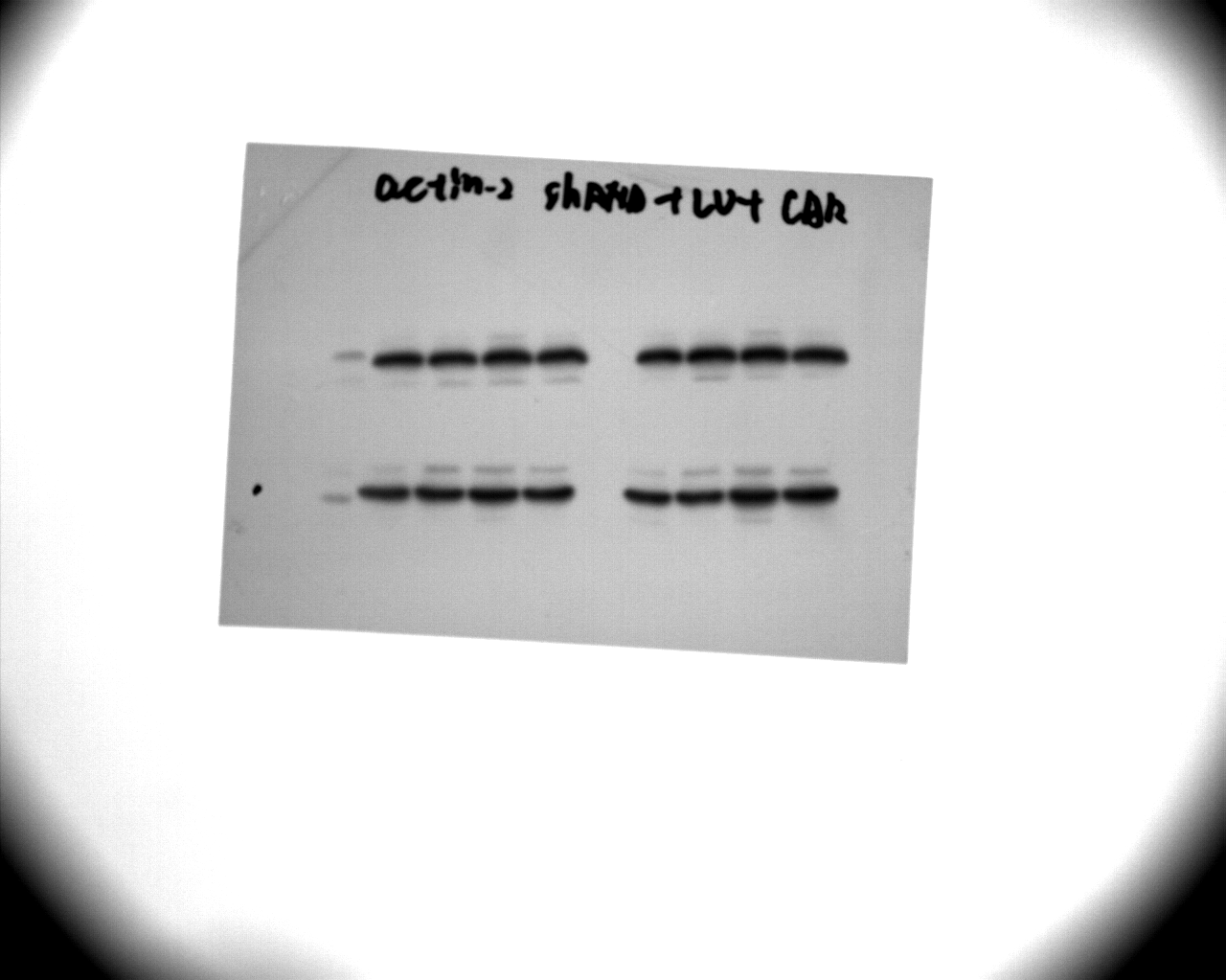

Supplement: S2 File — (ZIP) [file pone.0322733.s006.zip › Original Western Blot Images/Original Western Blot Images/Fig.7D/actin.tif]

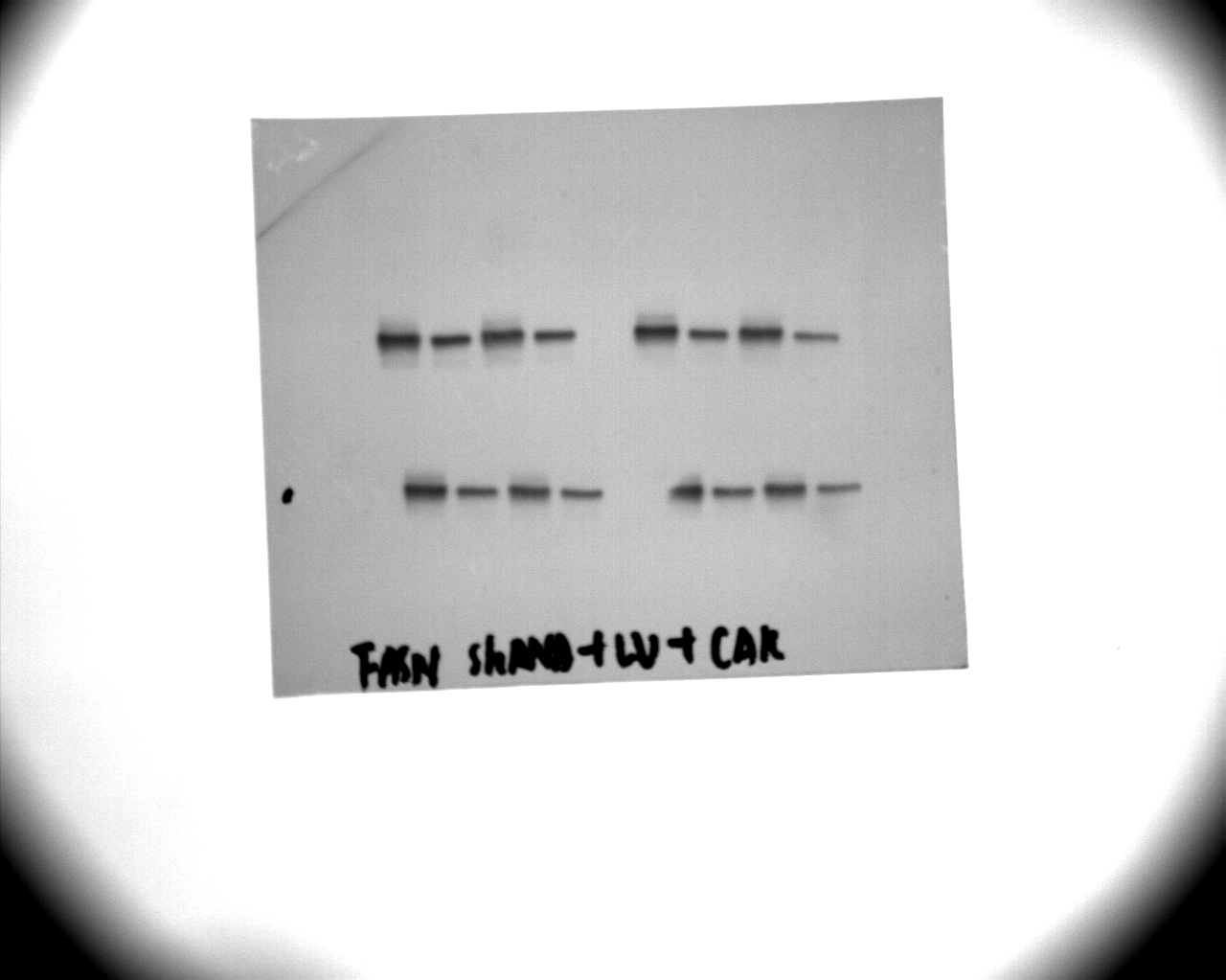

Supplement: S2 File — (ZIP) [file pone.0322733.s006.zip › Original Western Blot Images/Original Western Blot Images/Fig.7D/FASN.tif]

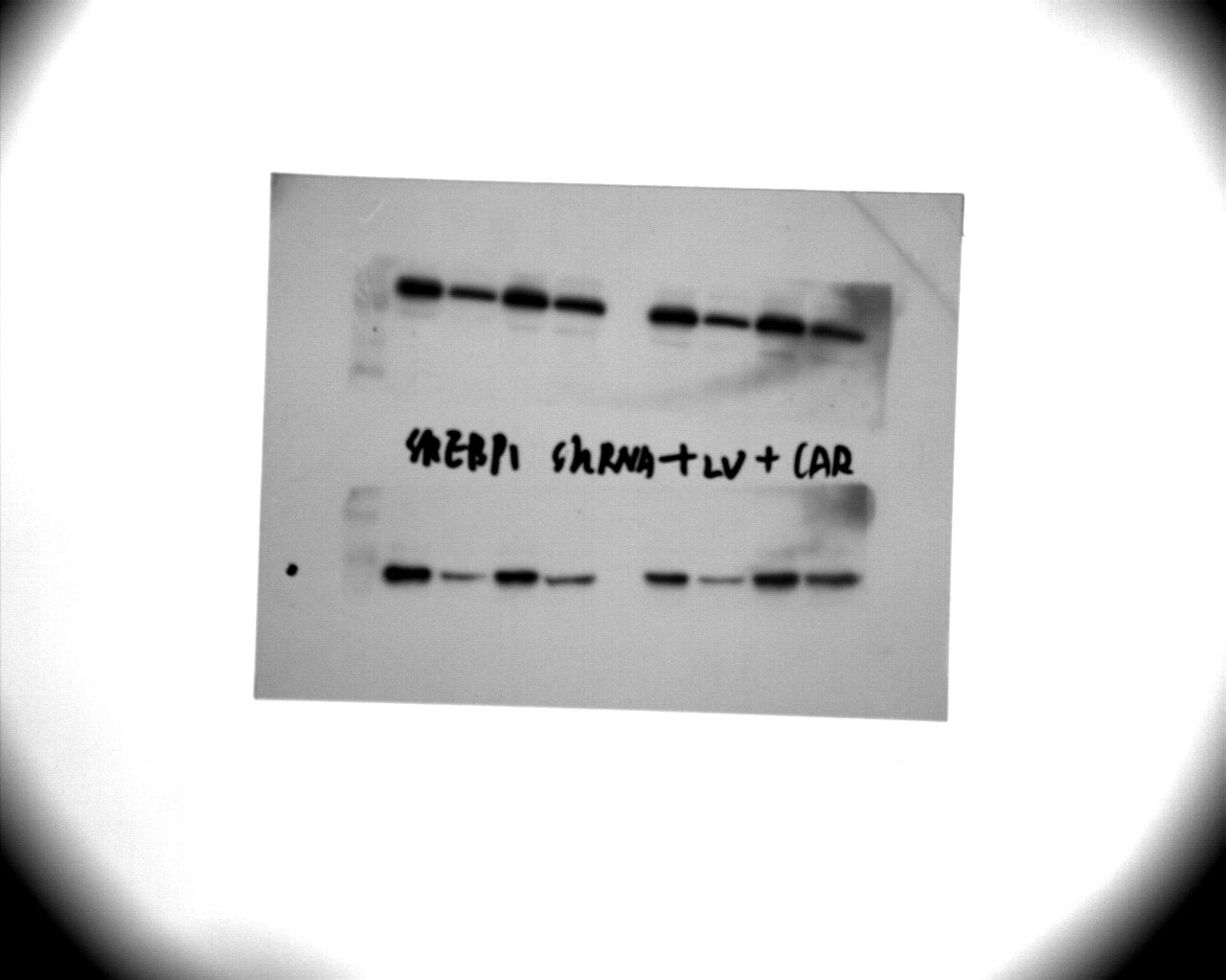

Supplement: S2 File — (ZIP) [file pone.0322733.s006.zip › Original Western Blot Images/Original Western Blot Images/Fig.7D/SREBP1.tif]

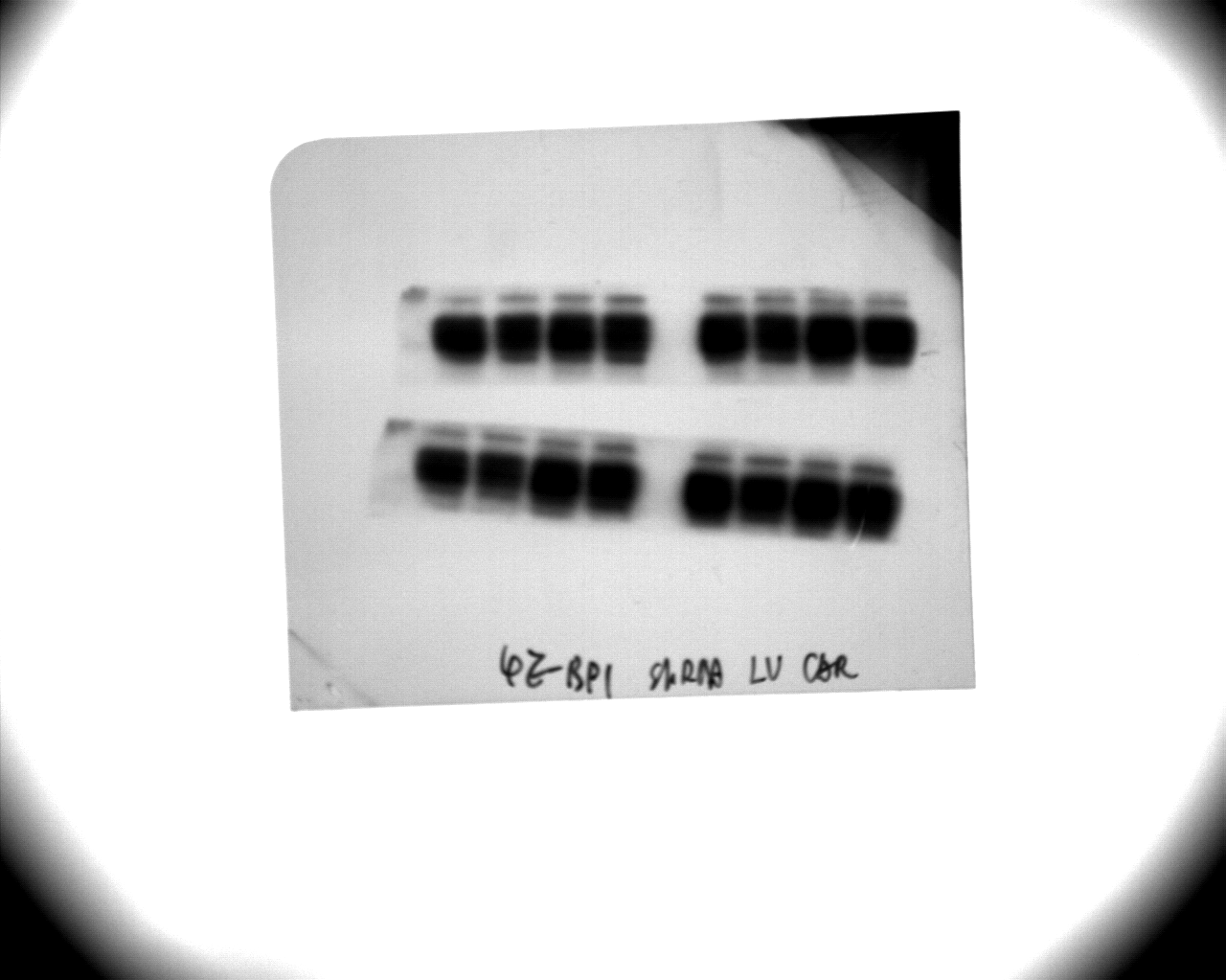

Supplement: S2 File — (ZIP) [file pone.0322733.s006.zip › Original Western Blot Images/Original Western Blot Images/Fig.7C/4E-BP1.tif]

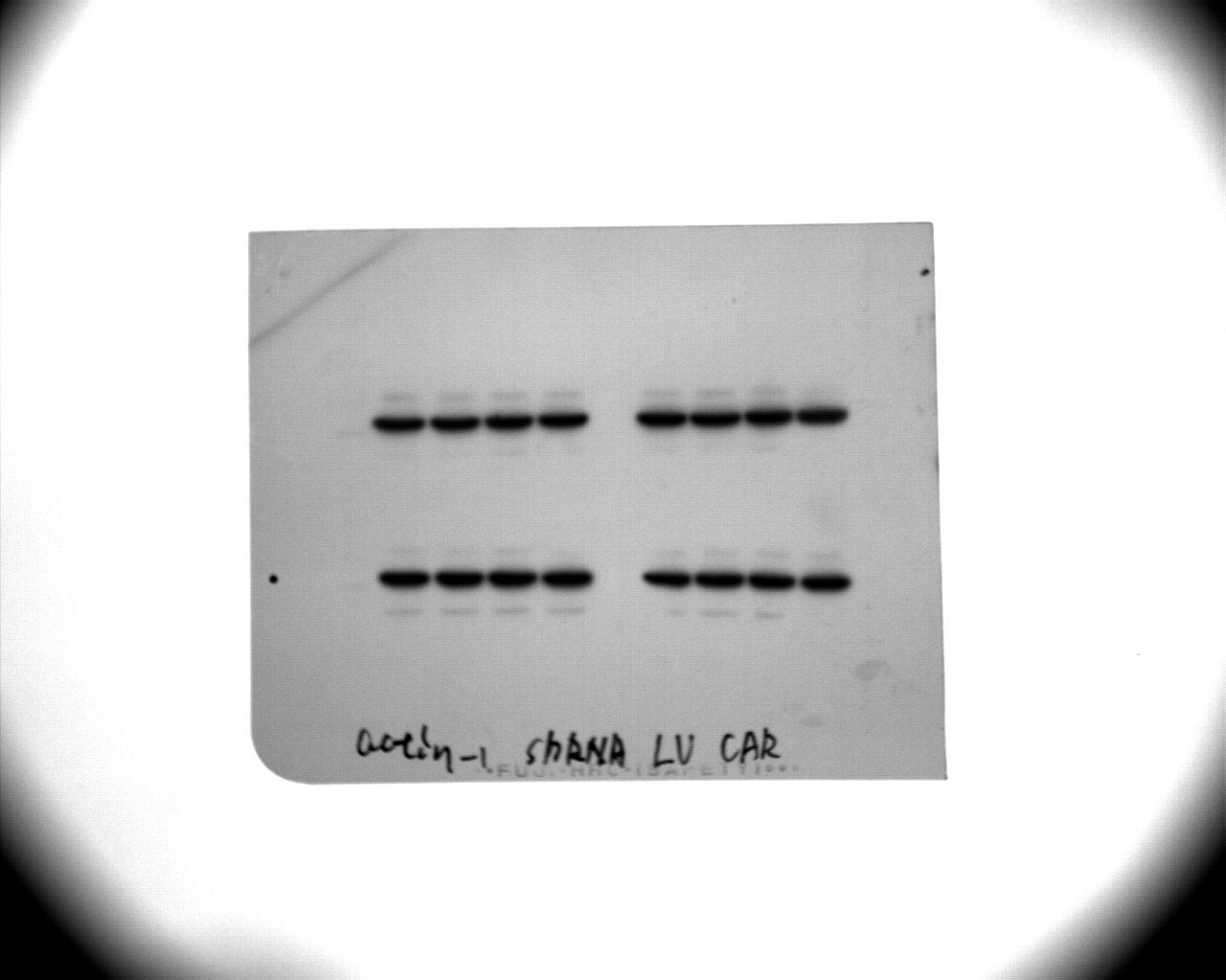

Supplement: S2 File — (ZIP) [file pone.0322733.s006.zip › Original Western Blot Images/Original Western Blot Images/Fig.7C/actin.tif]

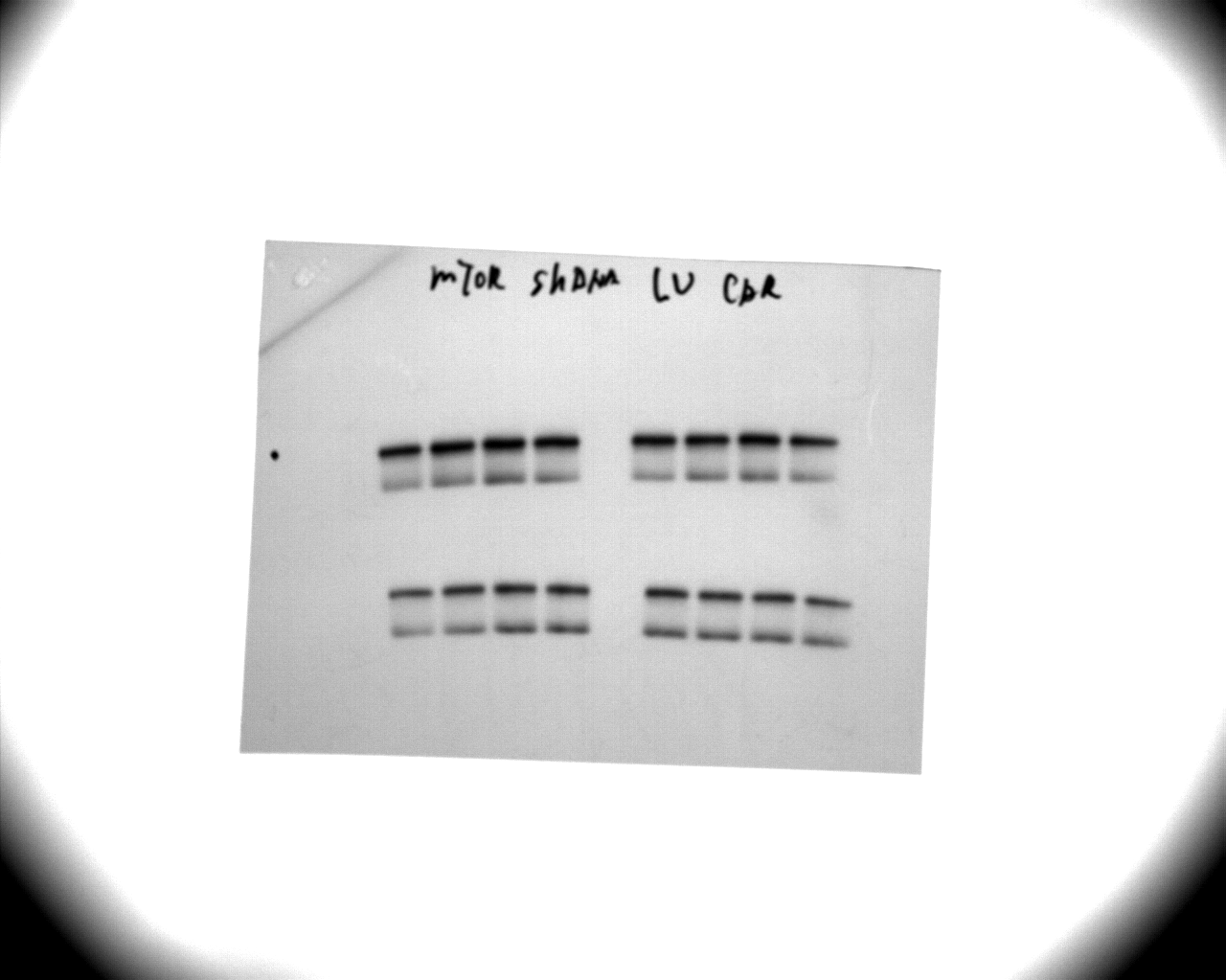

Supplement: S2 File — (ZIP) [file pone.0322733.s006.zip › Original Western Blot Images/Original Western Blot Images/Fig.7C/mTOR.tif]

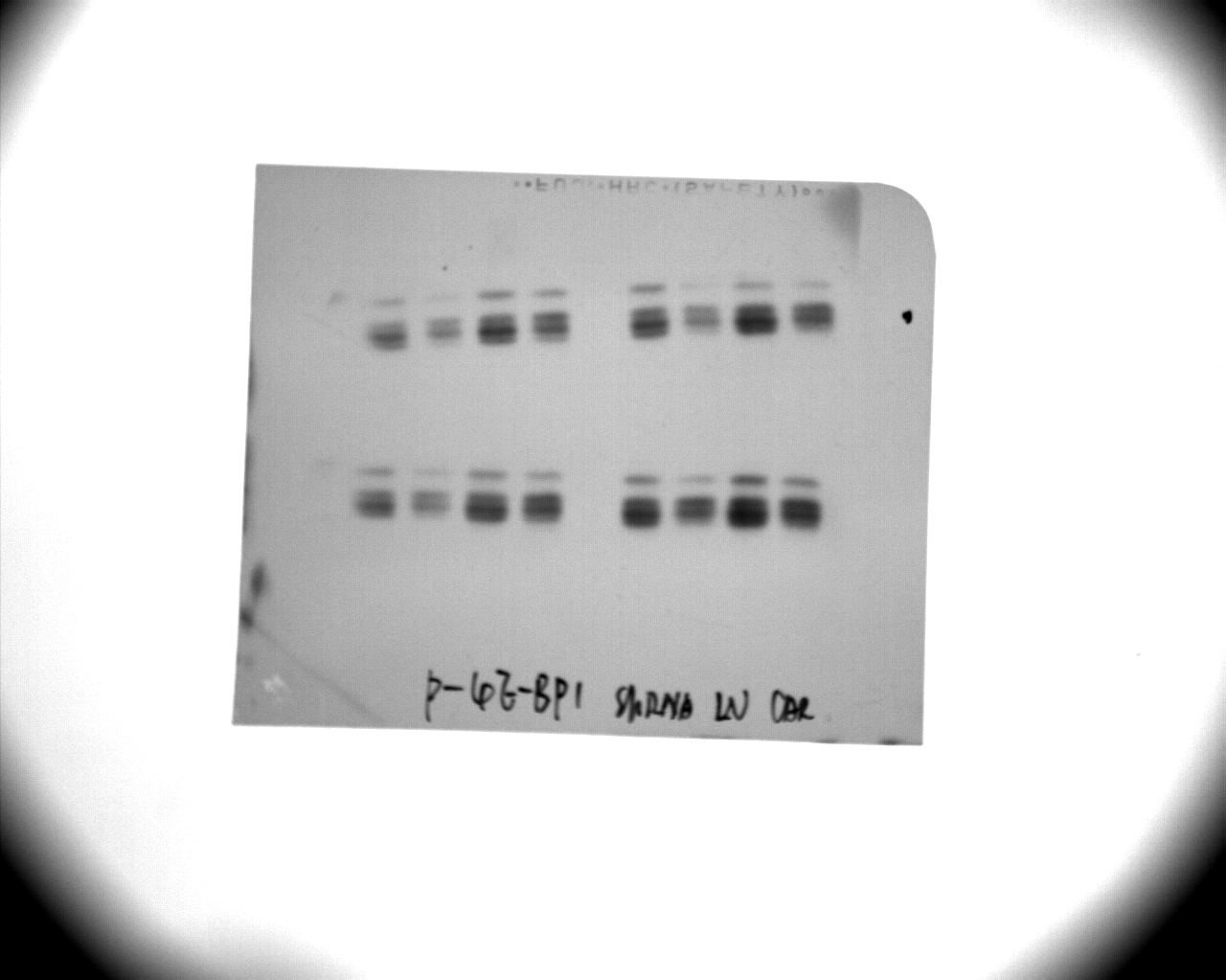

Supplement: S2 File — (ZIP) [file pone.0322733.s006.zip › Original Western Blot Images/Original Western Blot Images/Fig.7C/p-4E-BP1.tif]

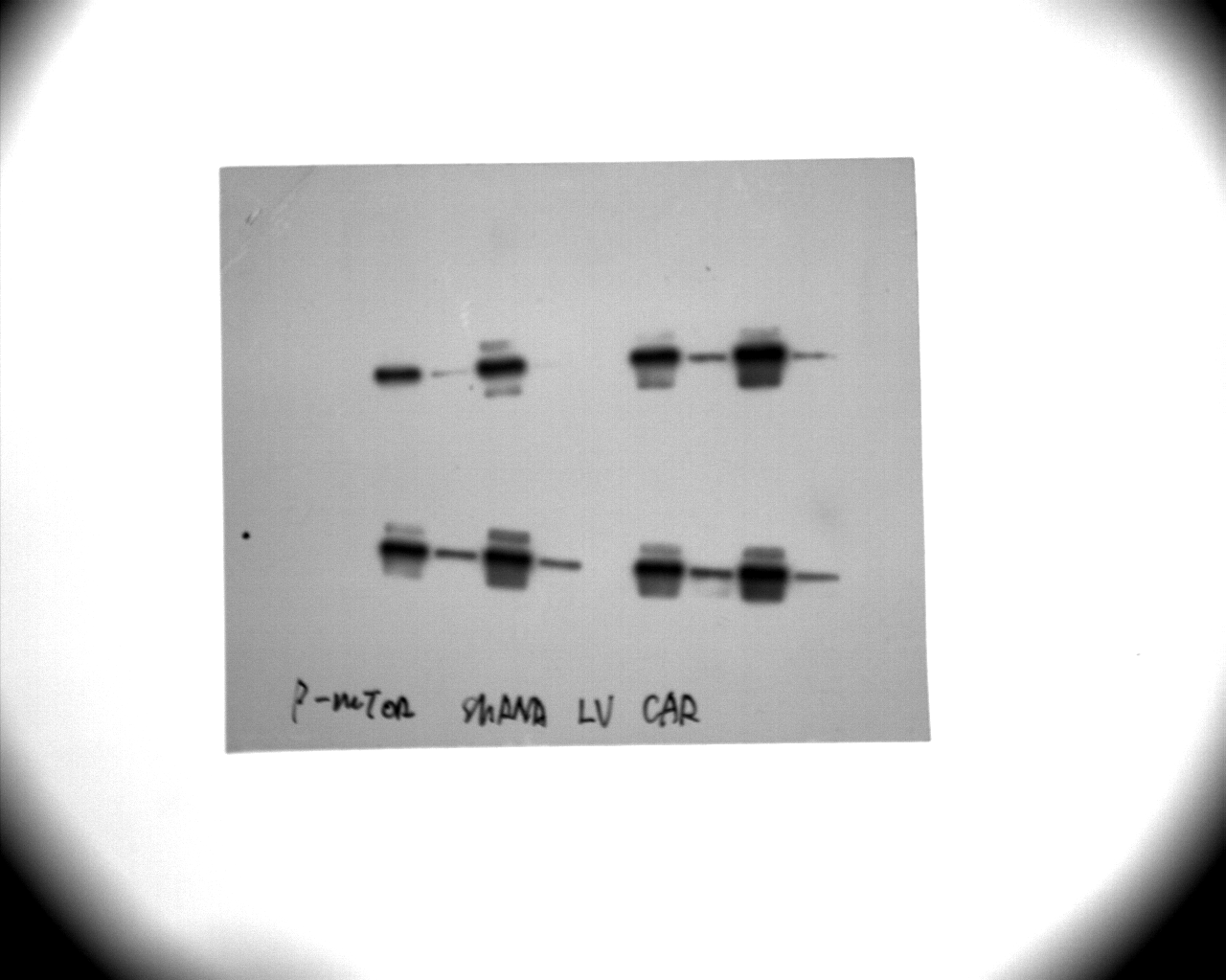

Supplement: S2 File — (ZIP) [file pone.0322733.s006.zip › Original Western Blot Images/Original Western Blot Images/Fig.7C/p-mTOR.tif]

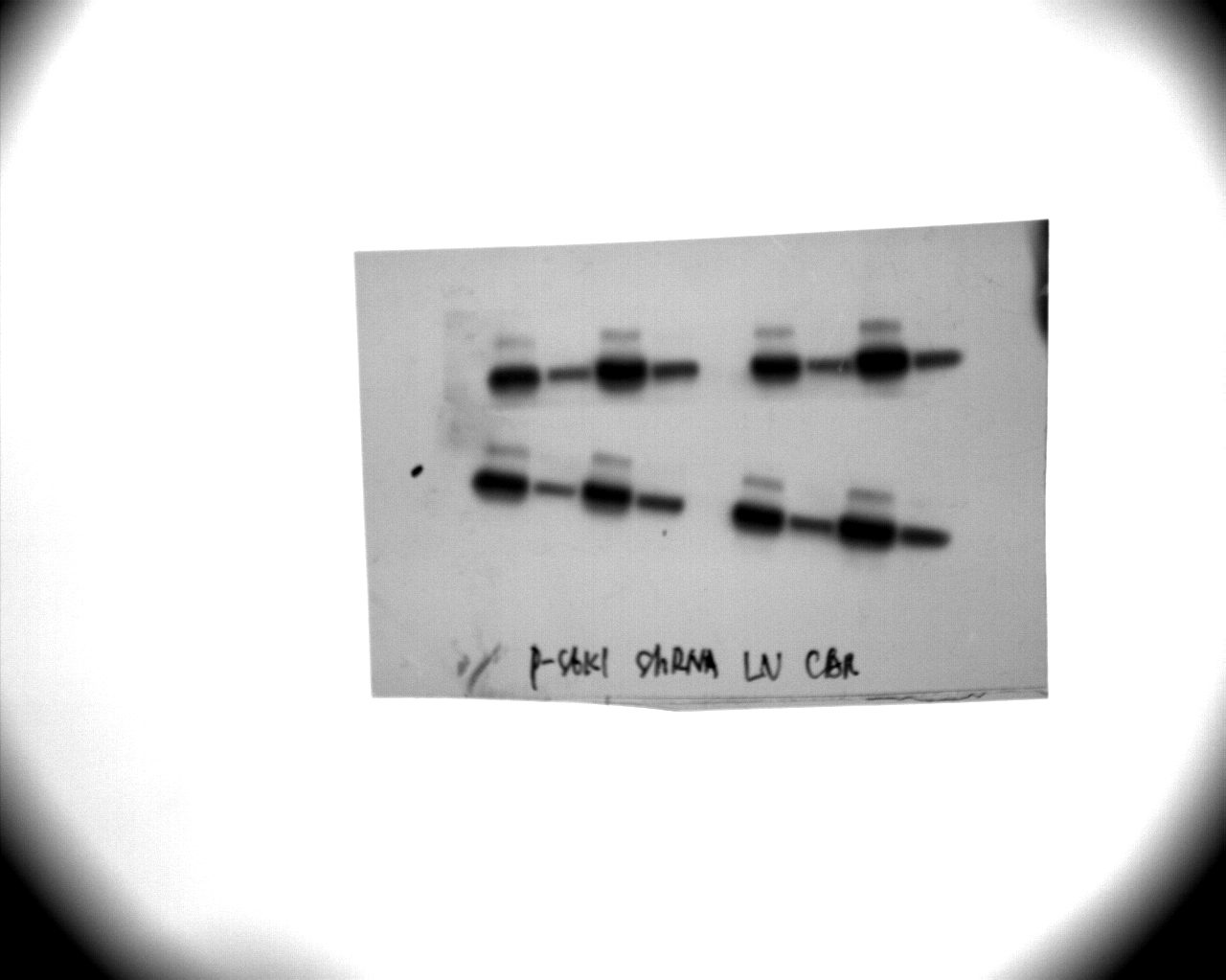

Supplement: S2 File — (ZIP) [file pone.0322733.s006.zip › Original Western Blot Images/Original Western Blot Images/Fig.7C/p-S6K1.tif]

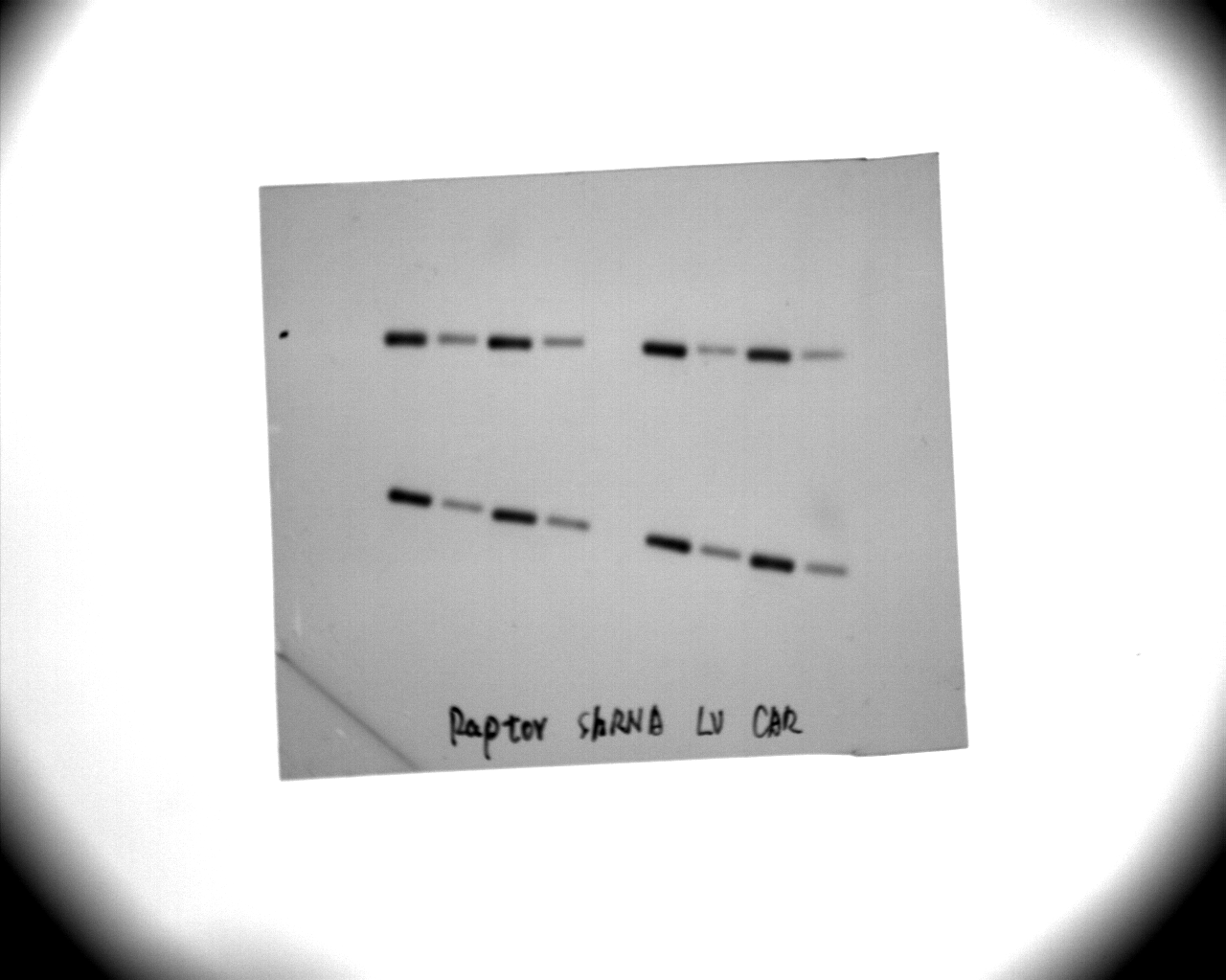

Supplement: S2 File — (ZIP) [file pone.0322733.s006.zip › Original Western Blot Images/Original Western Blot Images/Fig.7C/Raptor.tif]

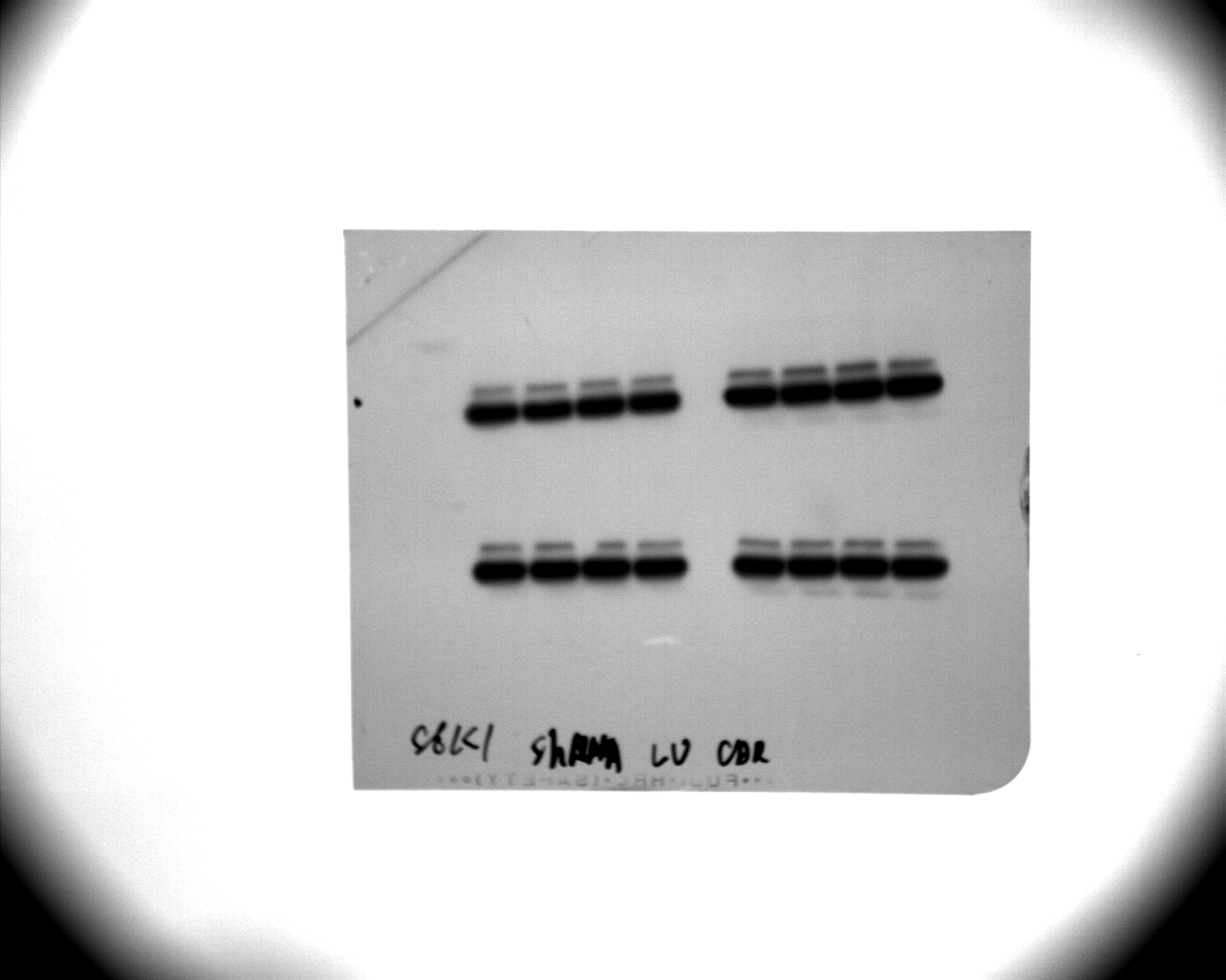

Supplement: S2 File — (ZIP) [file pone.0322733.s006.zip › Original Western Blot Images/Original Western Blot Images/Fig.7C/S6K1.tif]

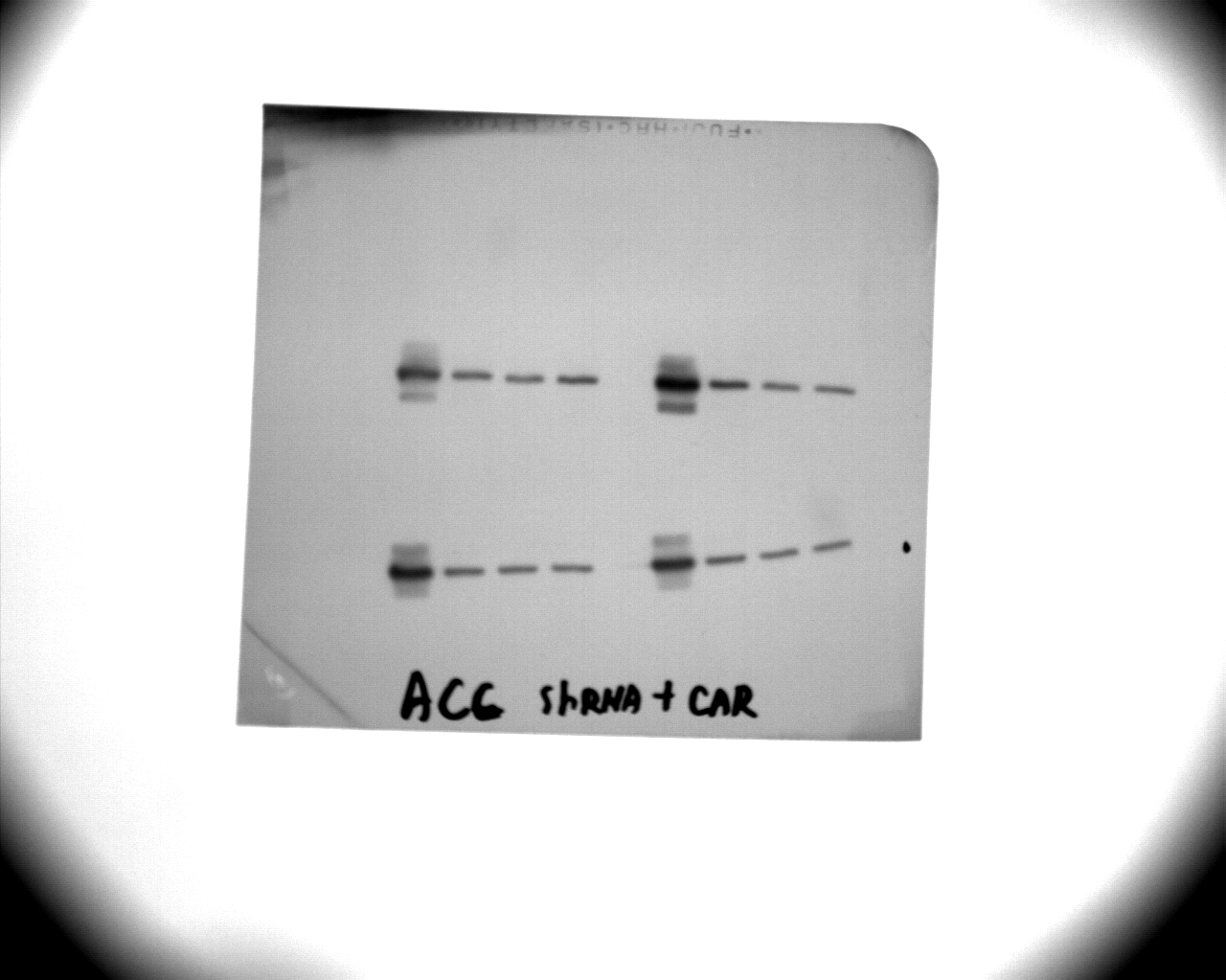

Supplement: S2 File — (ZIP) [file pone.0322733.s006.zip › Original Western Blot Images/Original Western Blot Images/Fig.7B/ACC.tif]

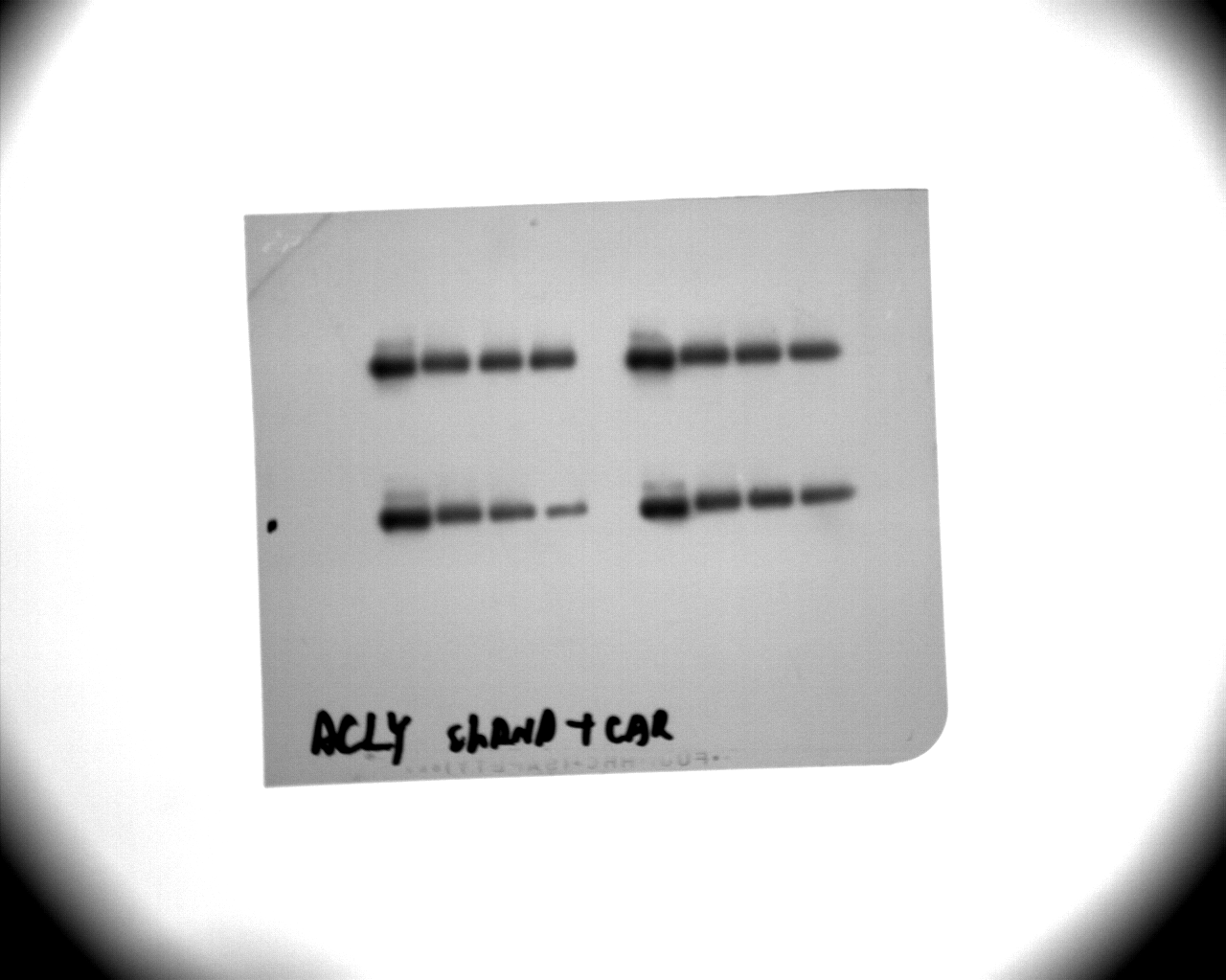

Supplement: S2 File — (ZIP) [file pone.0322733.s006.zip › Original Western Blot Images/Original Western Blot Images/Fig.7B/ACLY.tif]

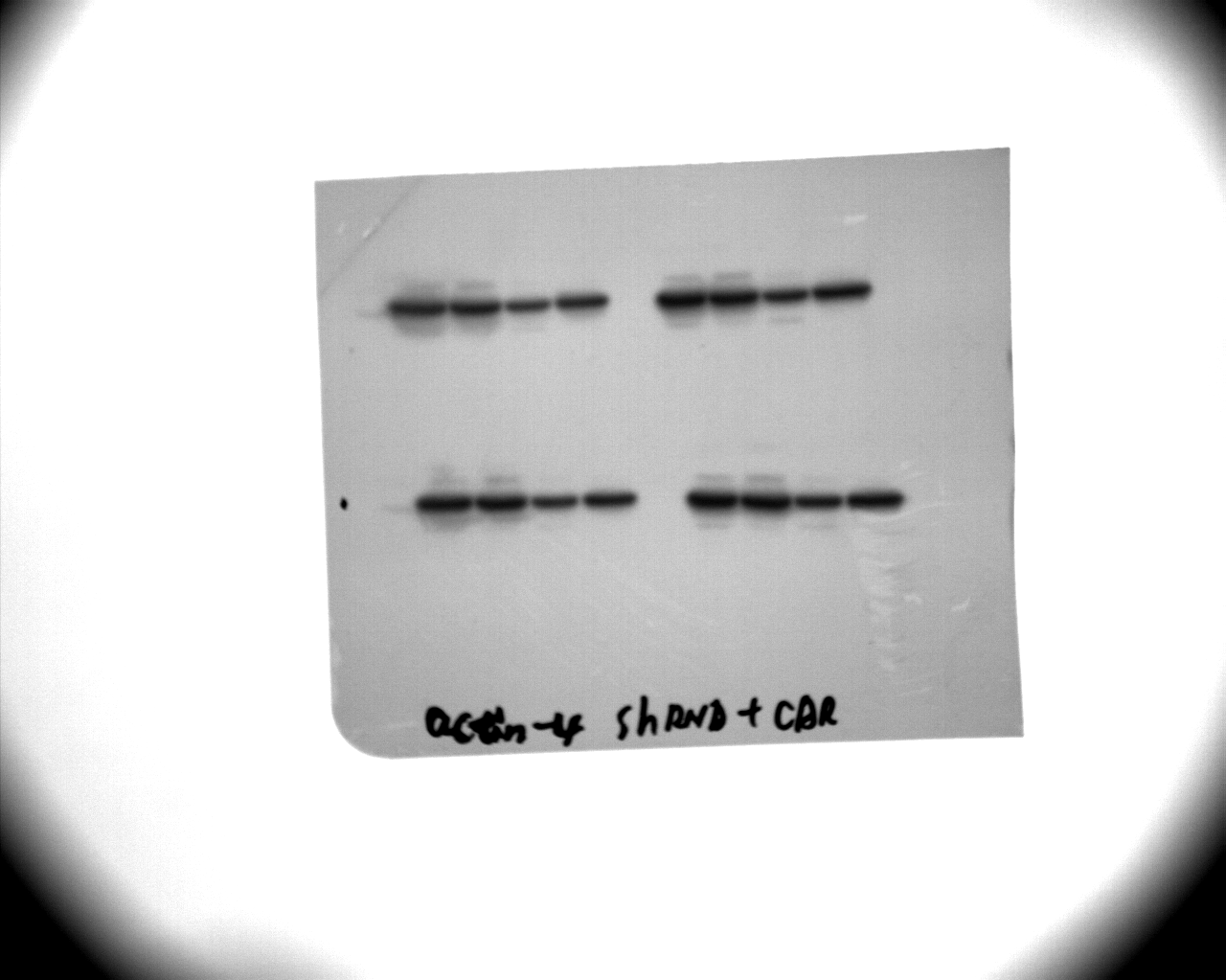

Supplement: S2 File — (ZIP) [file pone.0322733.s006.zip › Original Western Blot Images/Original Western Blot Images/Fig.7B/actin.tif]

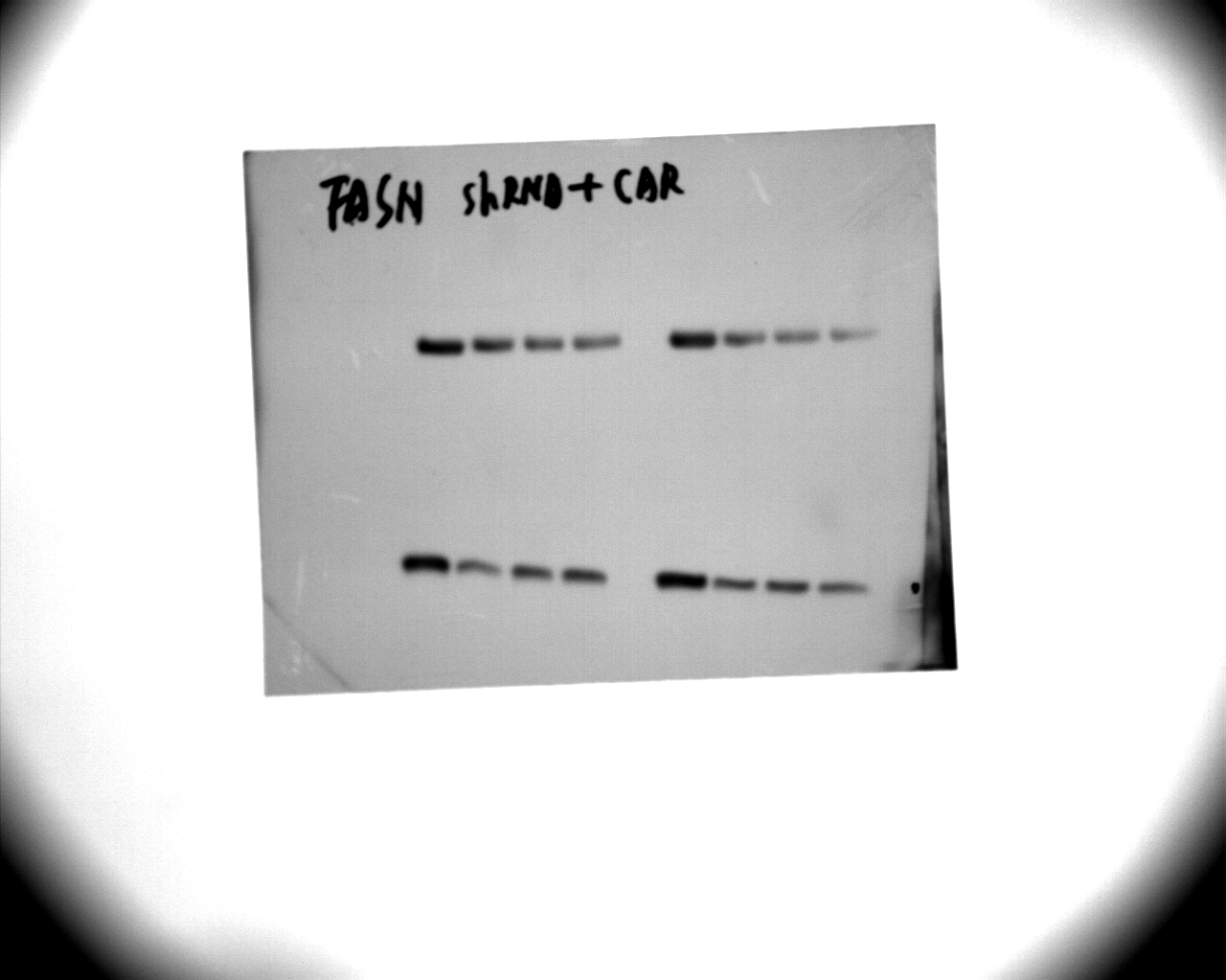

Supplement: S2 File — (ZIP) [file pone.0322733.s006.zip › Original Western Blot Images/Original Western Blot Images/Fig.7B/FASN.tif]

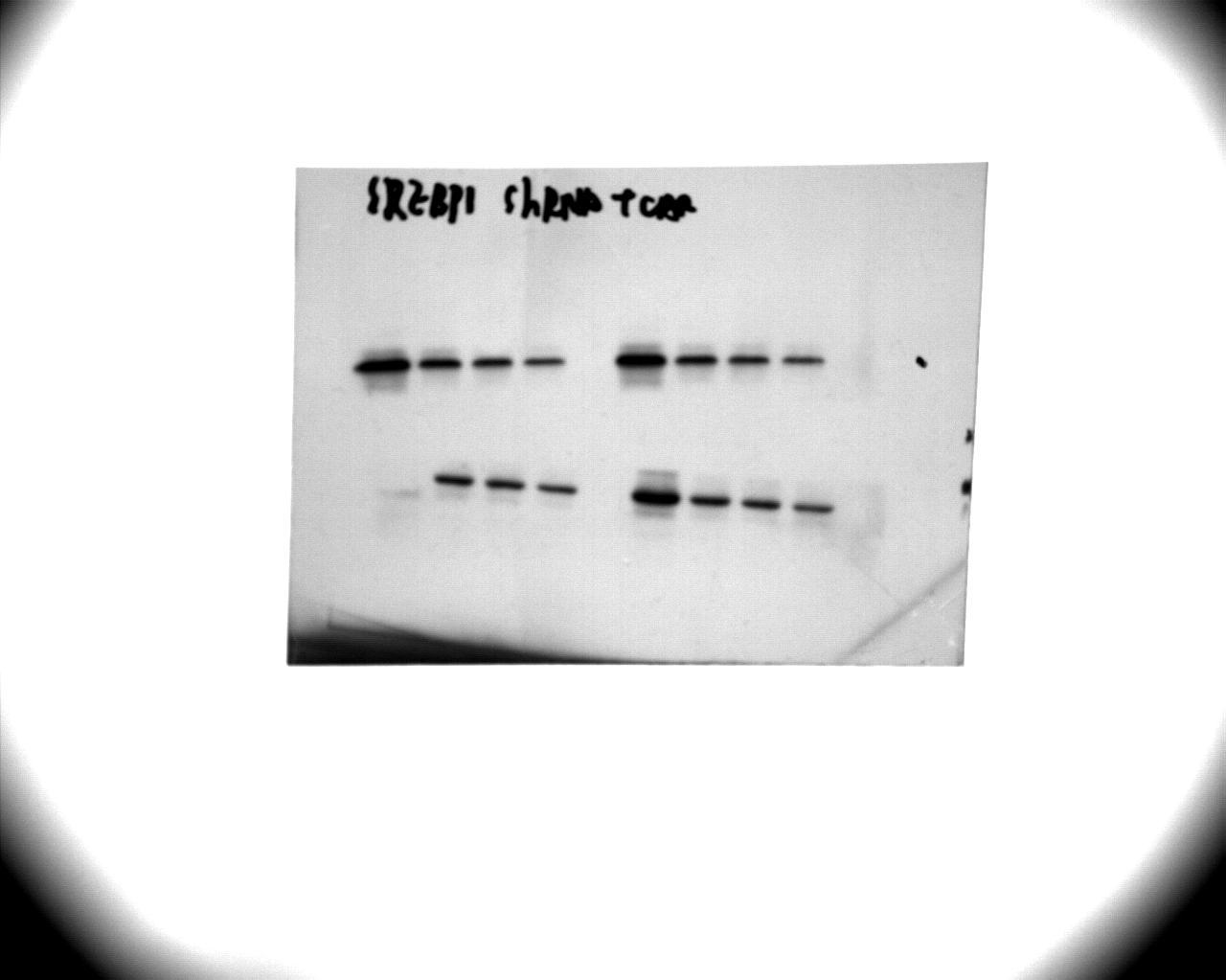

Supplement: S2 File — (ZIP) [file pone.0322733.s006.zip › Original Western Blot Images/Original Western Blot Images/Fig.7B/SREBP1.tif]

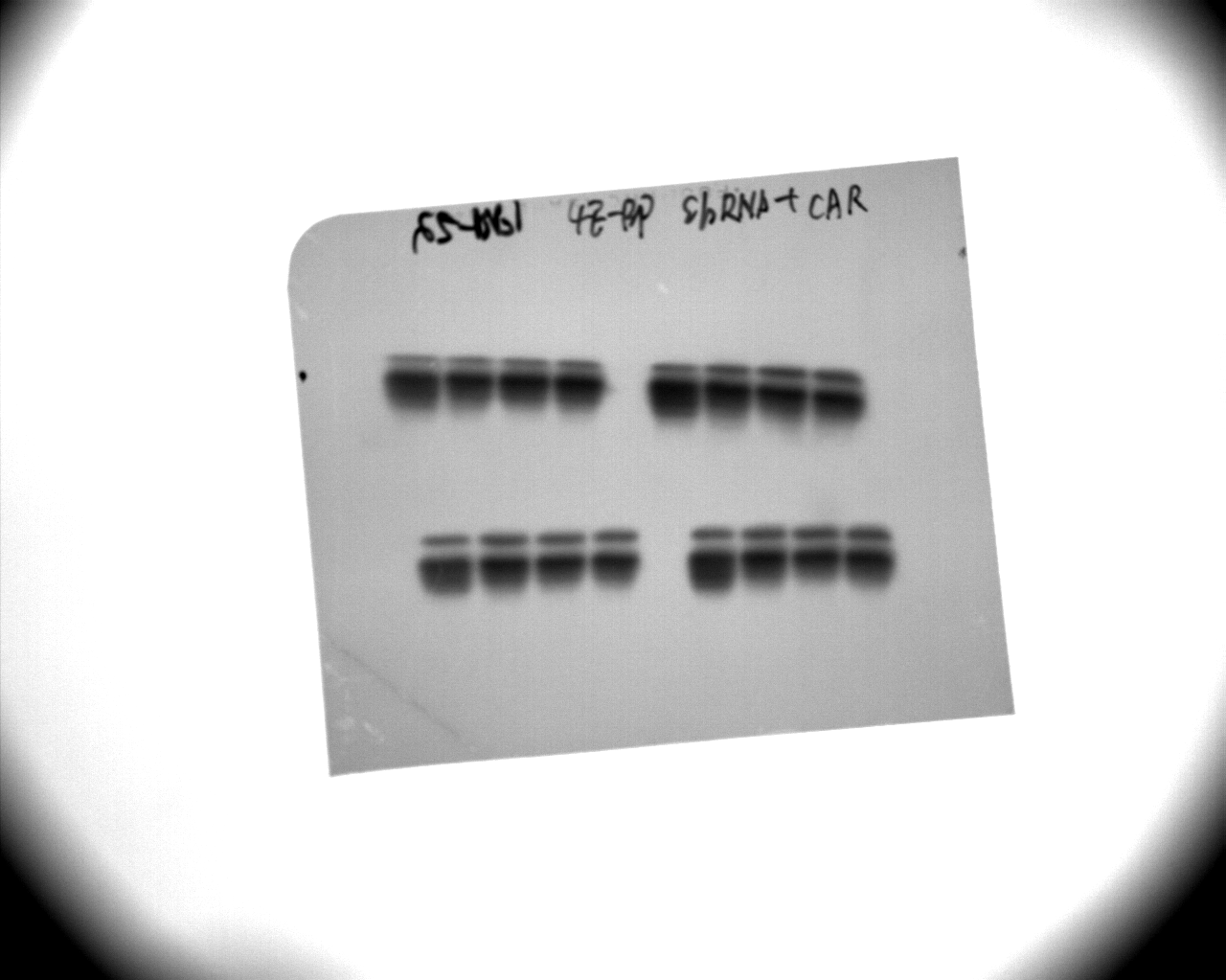

Supplement: S2 File — (ZIP) [file pone.0322733.s006.zip › Original Western Blot Images/Original Western Blot Images/Fig.7A/4E-BP1.tif]

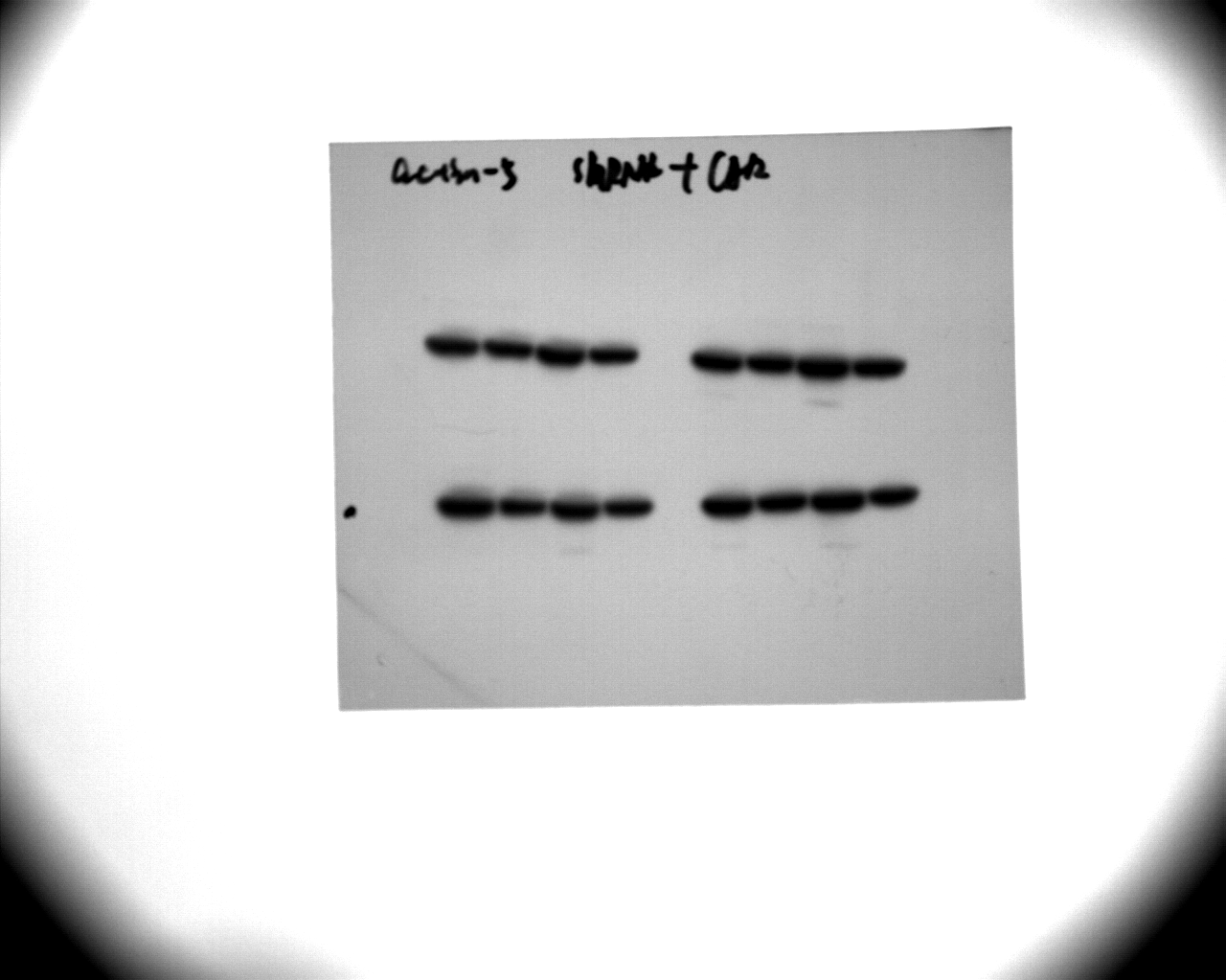

Supplement: S2 File — (ZIP) [file pone.0322733.s006.zip › Original Western Blot Images/Original Western Blot Images/Fig.7A/actin.tif]

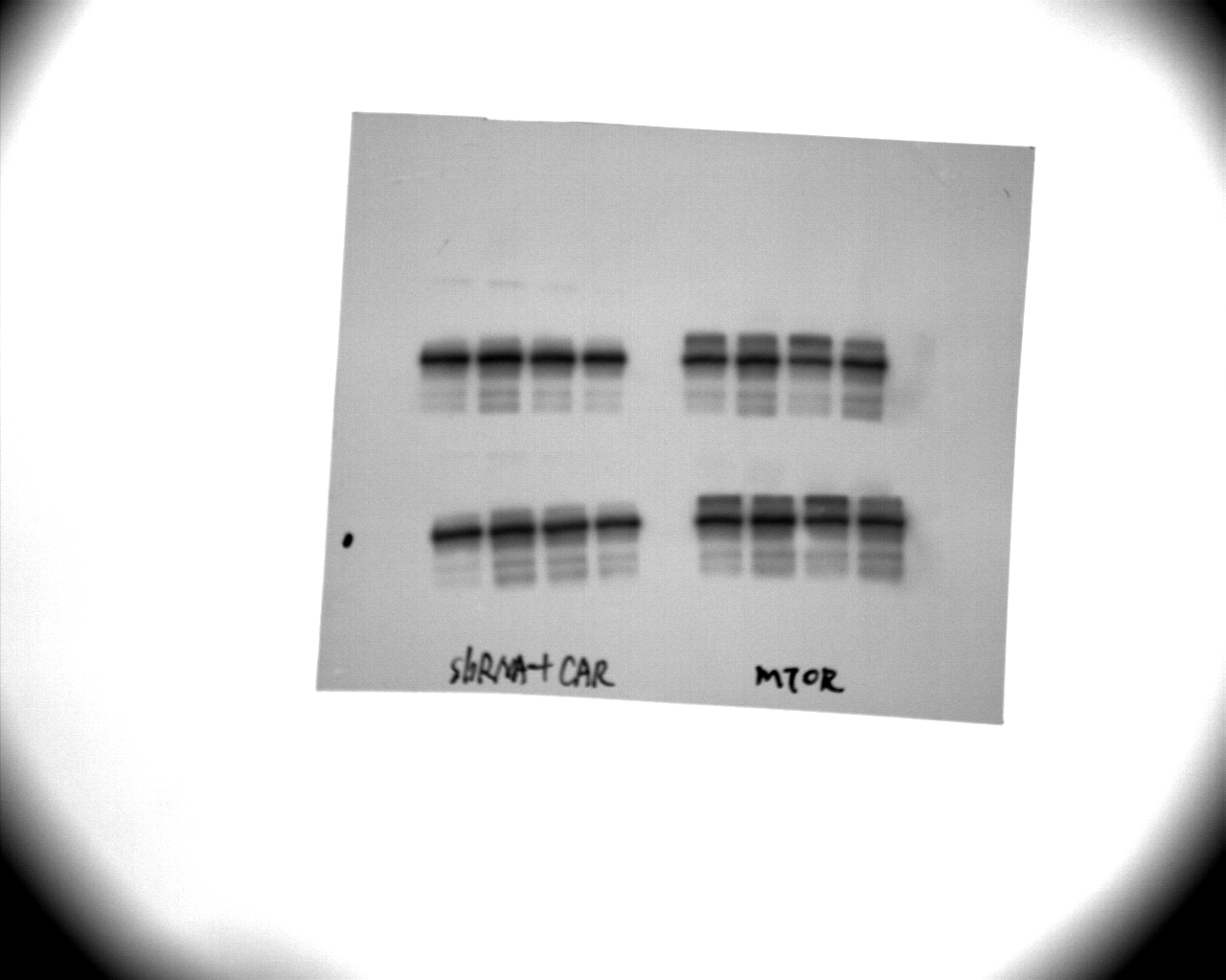

Supplement: S2 File — (ZIP) [file pone.0322733.s006.zip › Original Western Blot Images/Original Western Blot Images/Fig.7A/mTOR.tif]

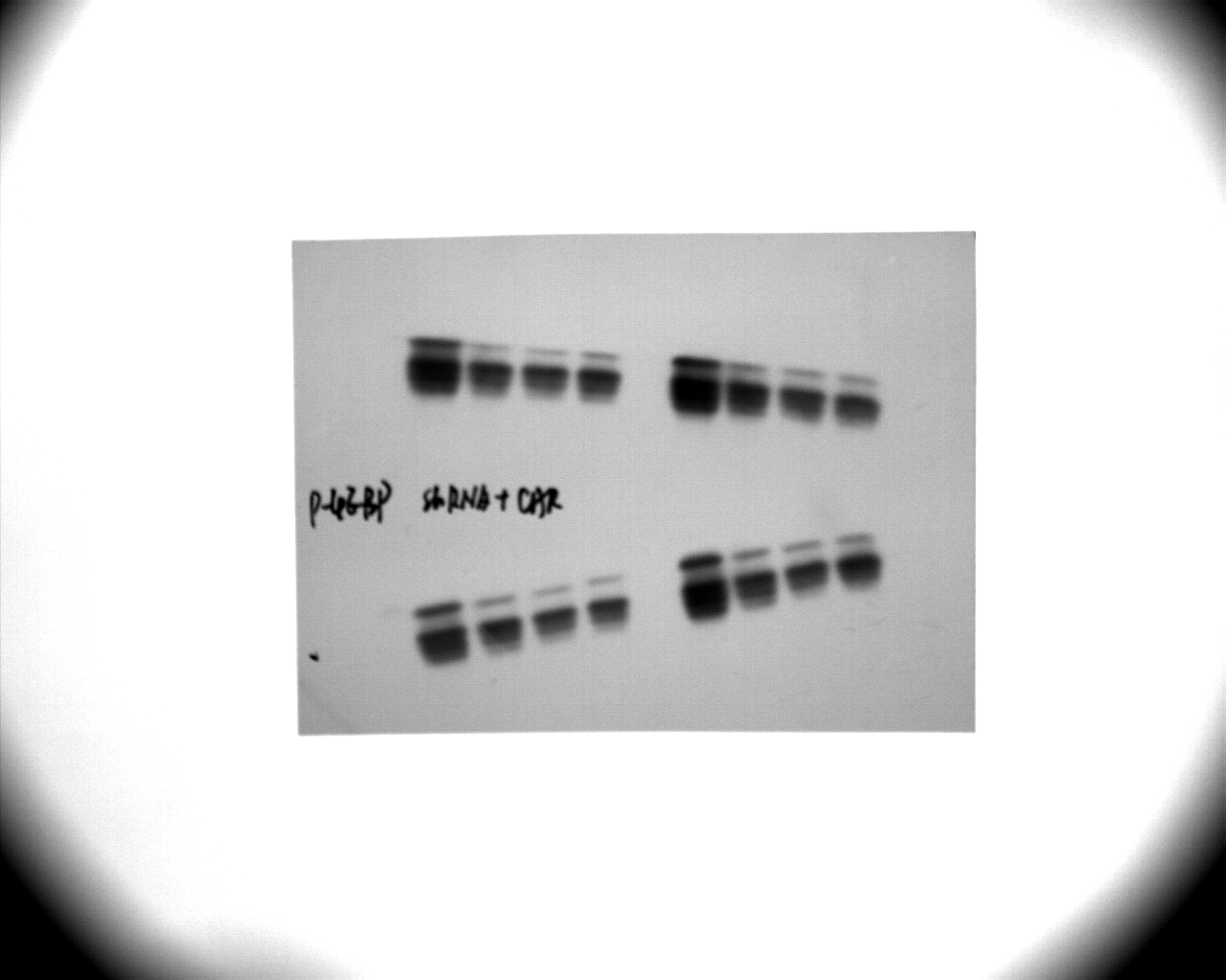

Supplement: S2 File — (ZIP) [file pone.0322733.s006.zip › Original Western Blot Images/Original Western Blot Images/Fig.7A/p-4E-BP1.tif]

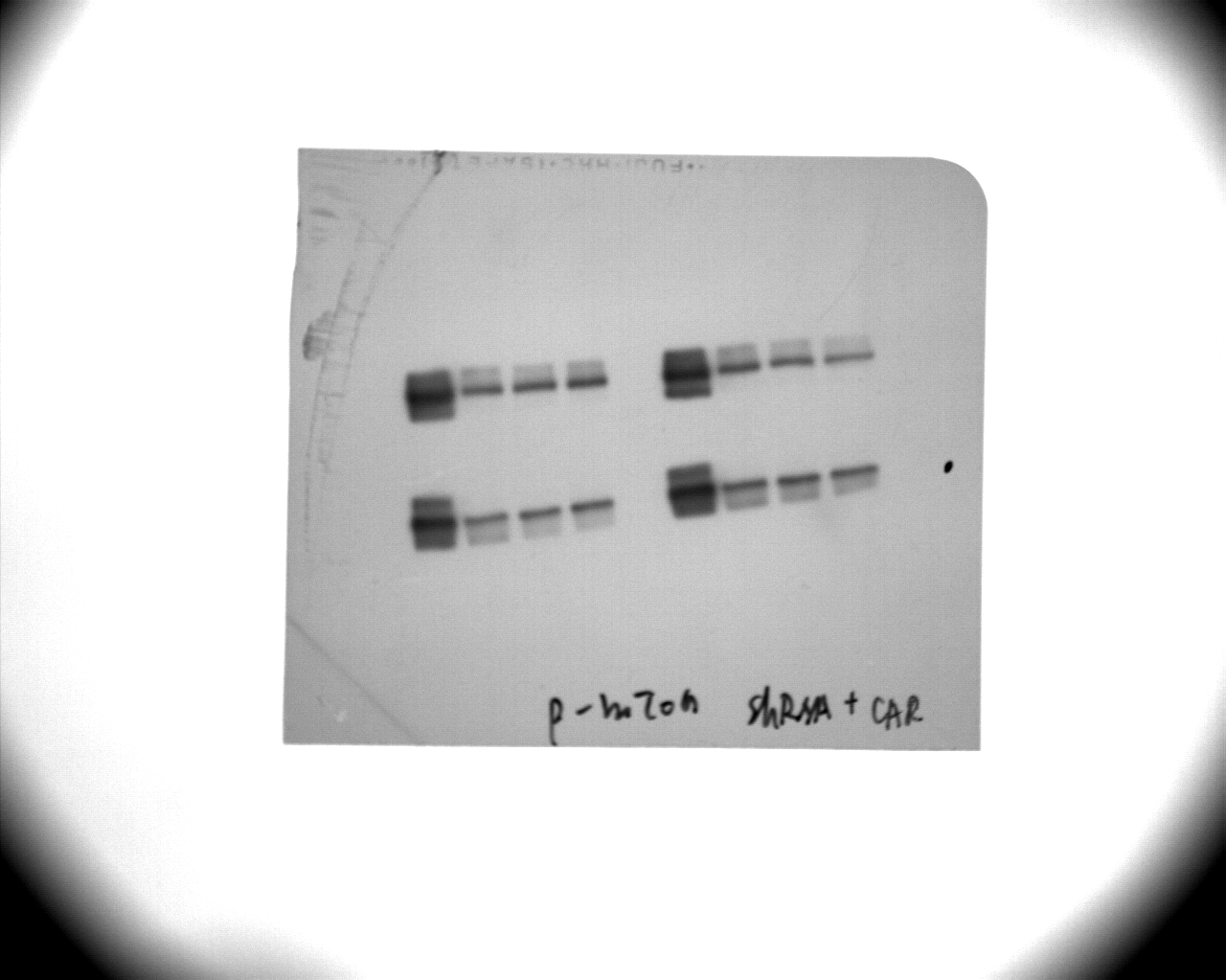

Supplement: S2 File — (ZIP) [file pone.0322733.s006.zip › Original Western Blot Images/Original Western Blot Images/Fig.7A/p-mTOR.tif]

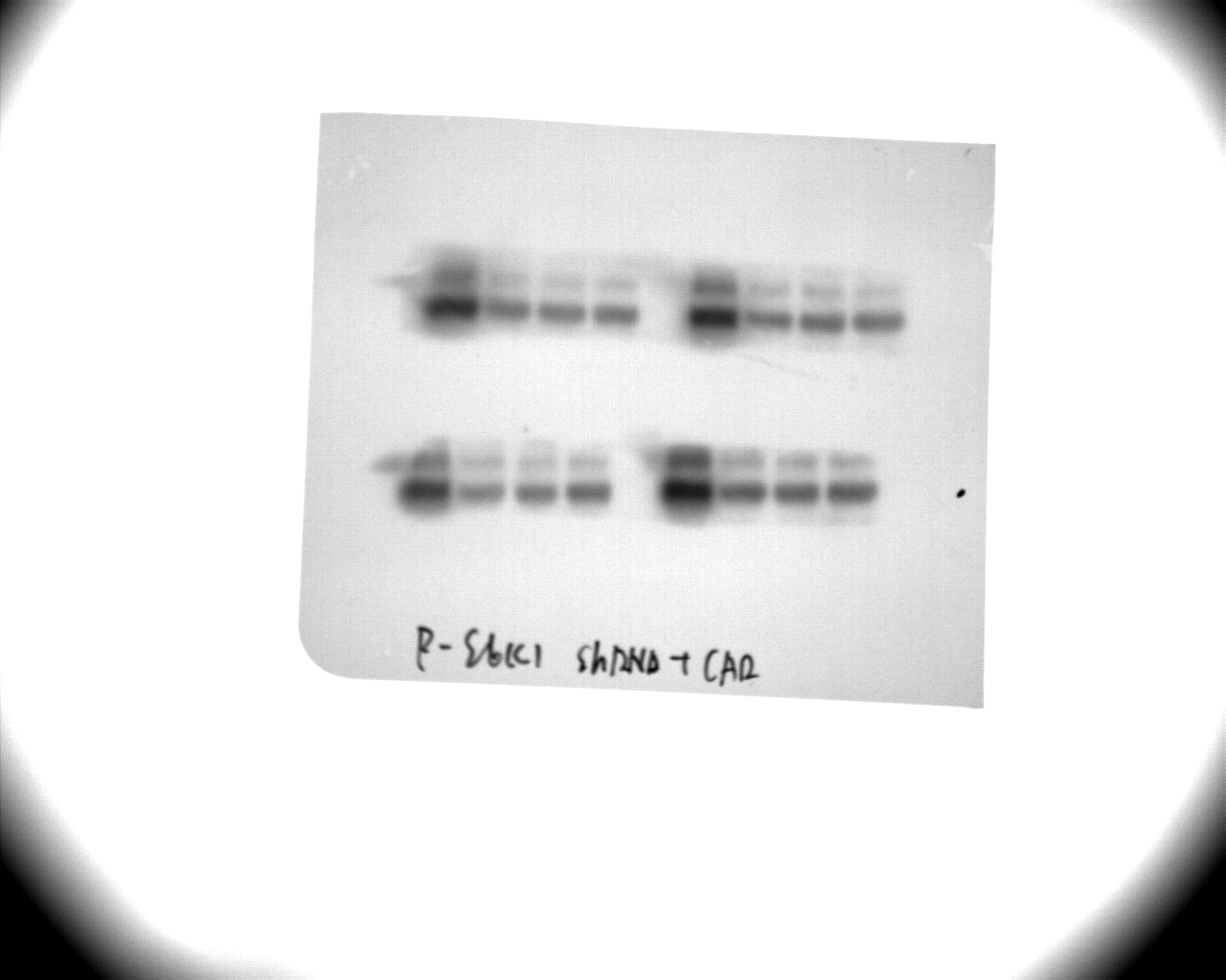

Supplement: S2 File — (ZIP) [file pone.0322733.s006.zip › Original Western Blot Images/Original Western Blot Images/Fig.7A/p-S6K1.tif]

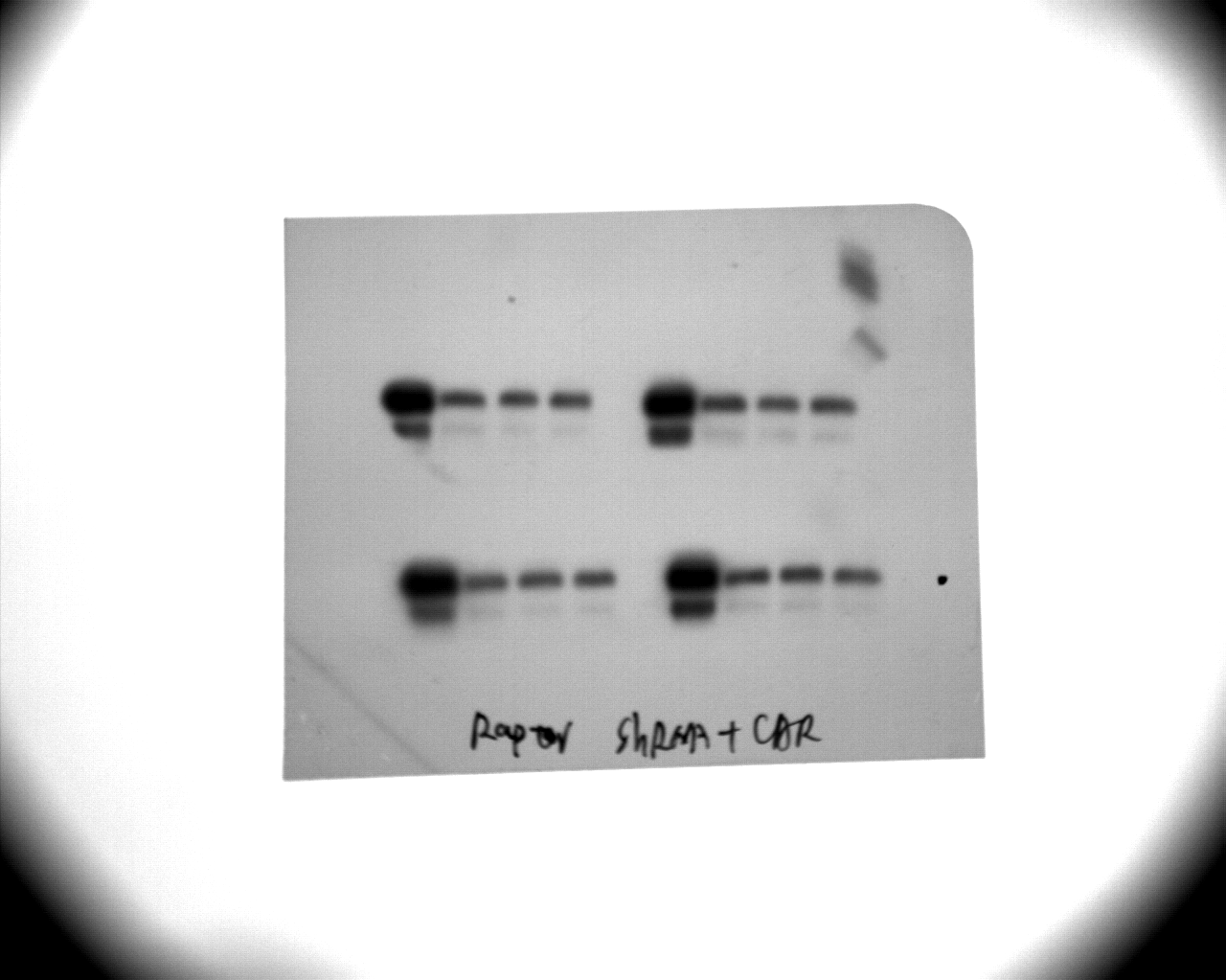

Supplement: S2 File — (ZIP) [file pone.0322733.s006.zip › Original Western Blot Images/Original Western Blot Images/Fig.7A/Raptor.tif]

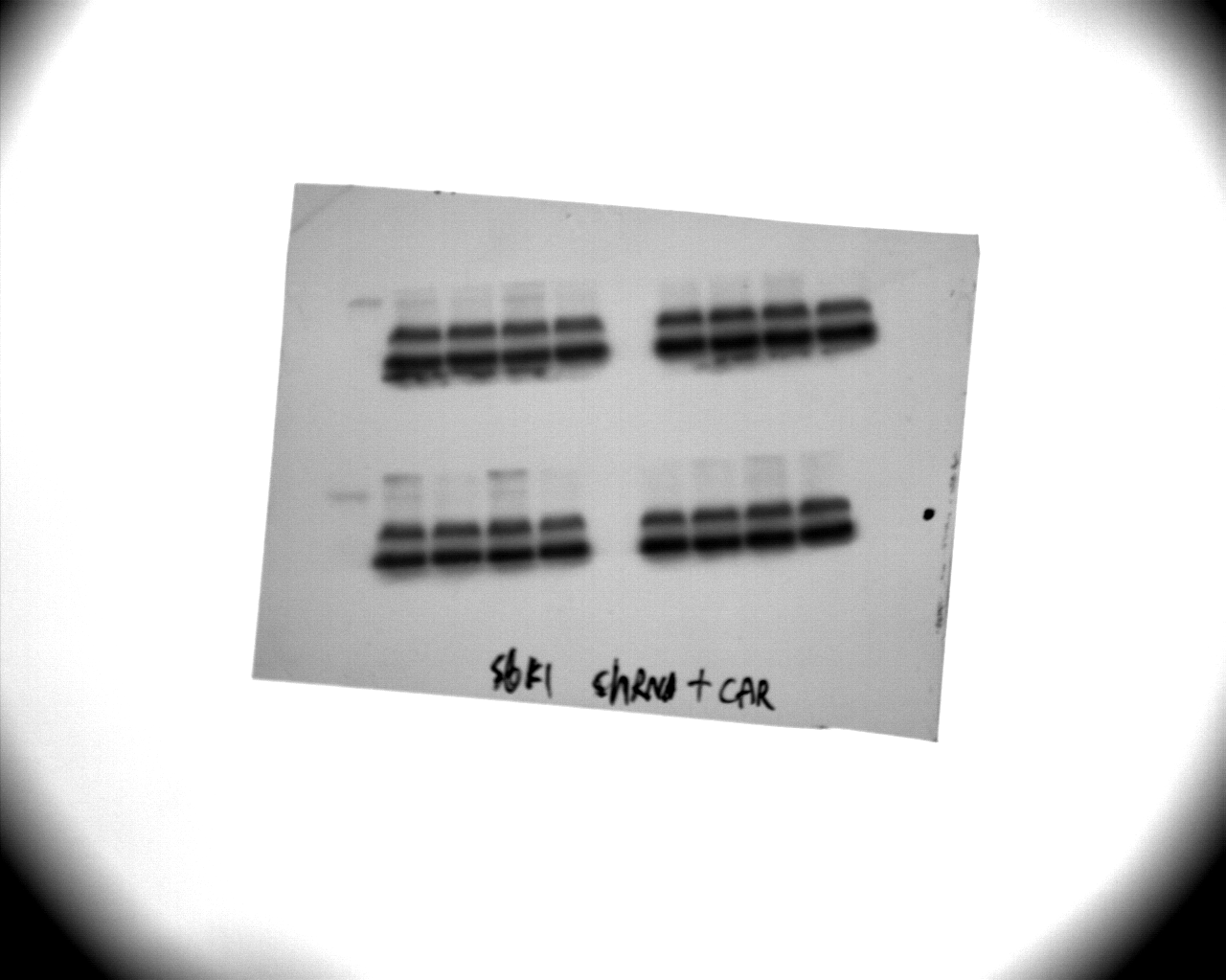

Supplement: S2 File — (ZIP) [file pone.0322733.s006.zip › Original Western Blot Images/Original Western Blot Images/Fig.7A/S6K1.tif]

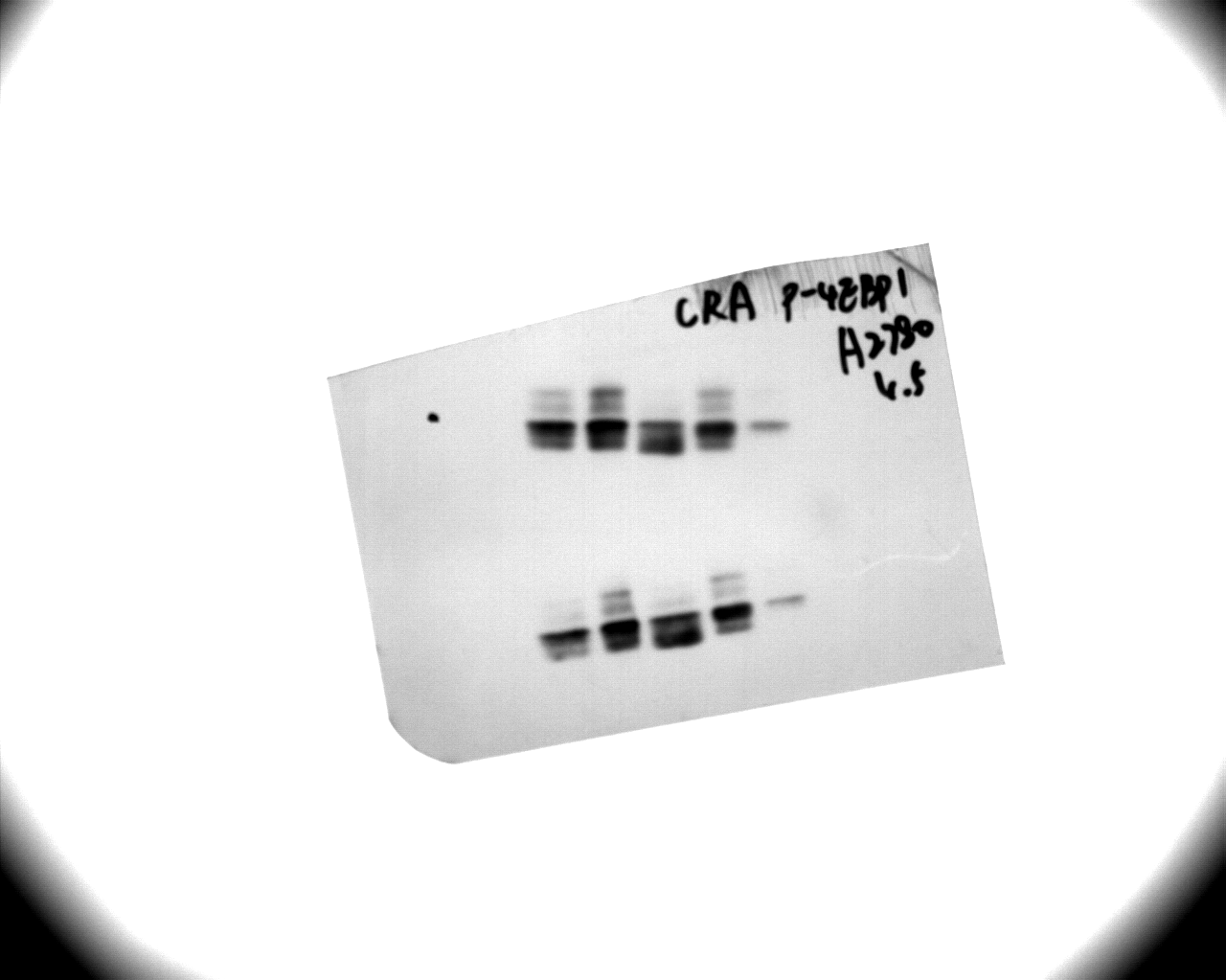

Supplement: S2 File — (ZIP) [file pone.0322733.s006.zip › Original Western Blot Images/Original Western Blot Images/Fig.5B/A2780-p-4E-BP1.tif]

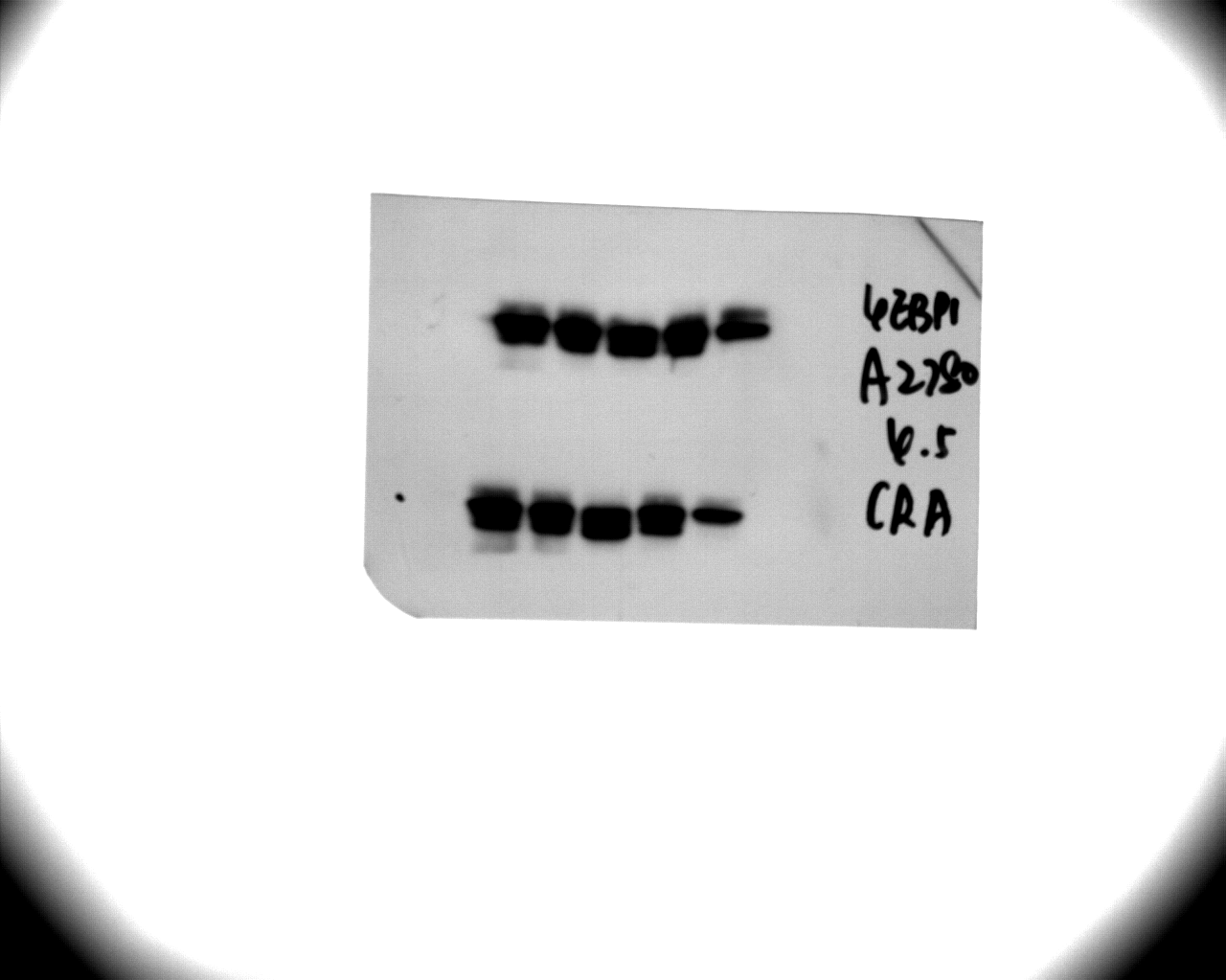

Supplement: S2 File — (ZIP) [file pone.0322733.s006.zip › Original Western Blot Images/Original Western Blot Images/Fig.5B/A2780-4E-BP1.tif]

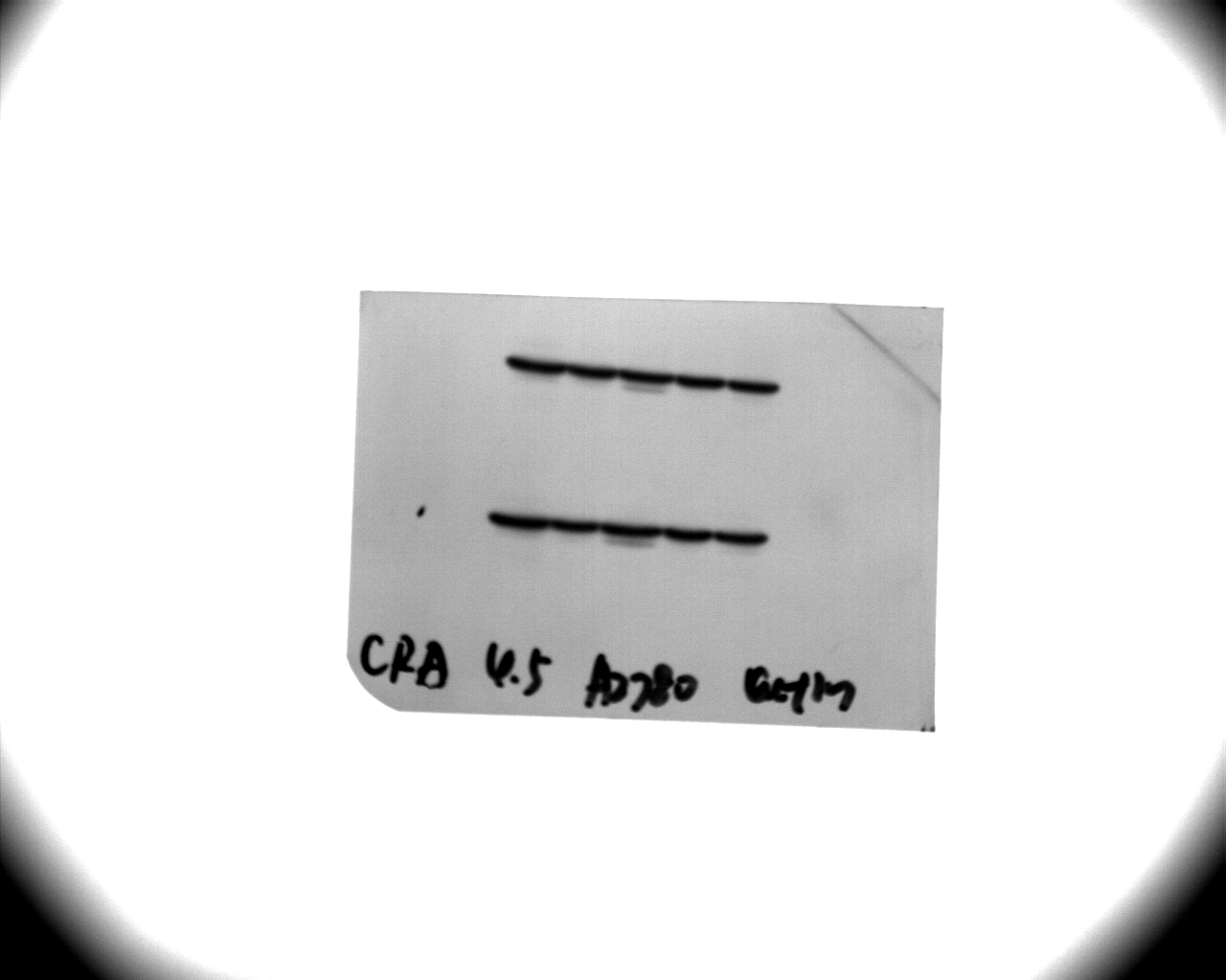

Supplement: S2 File — (ZIP) [file pone.0322733.s006.zip › Original Western Blot Images/Original Western Blot Images/Fig.5B/A2780-actin.tif]

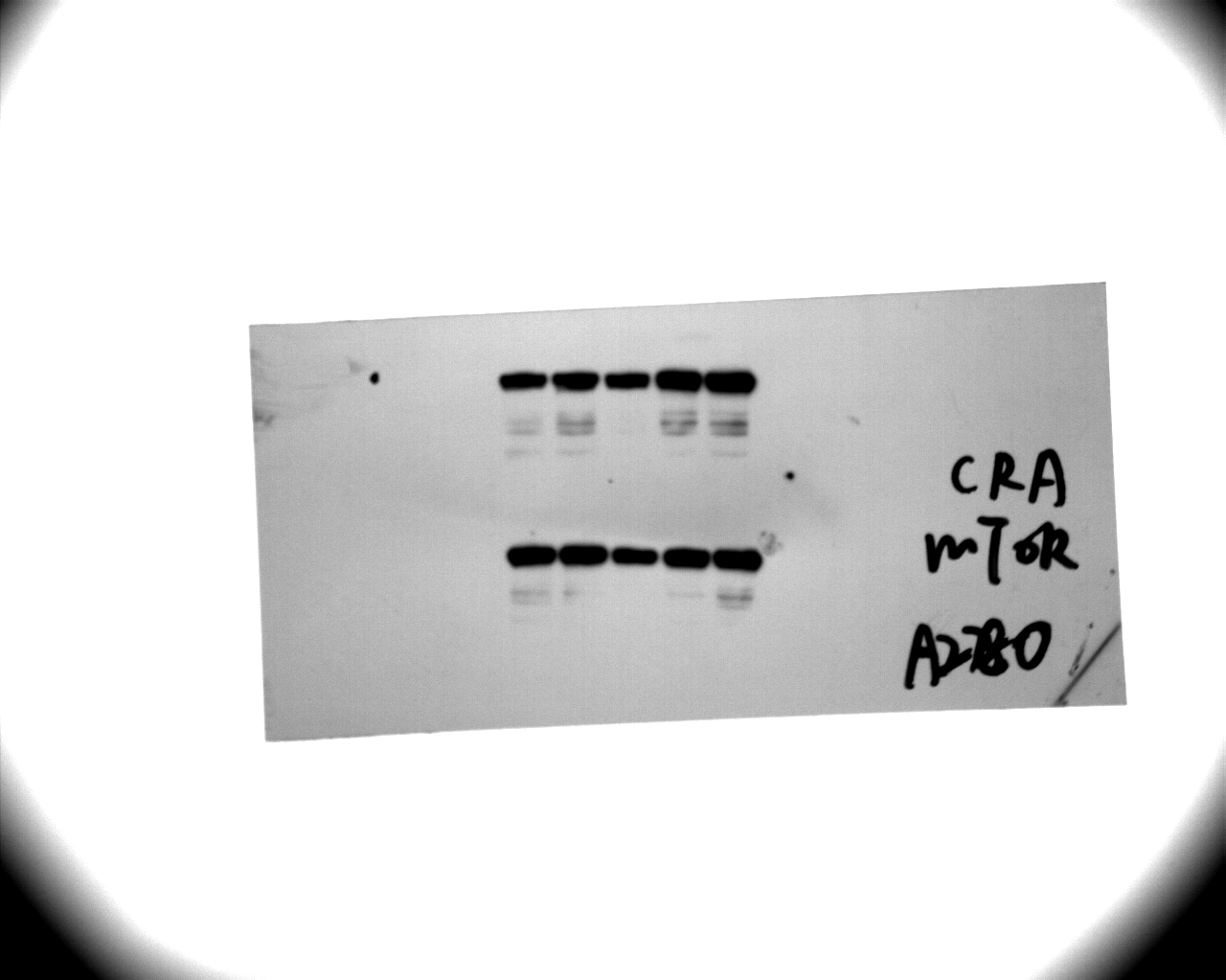

Supplement: S2 File — (ZIP) [file pone.0322733.s006.zip › Original Western Blot Images/Original Western Blot Images/Fig.5B/A2780-mTOR.tif]

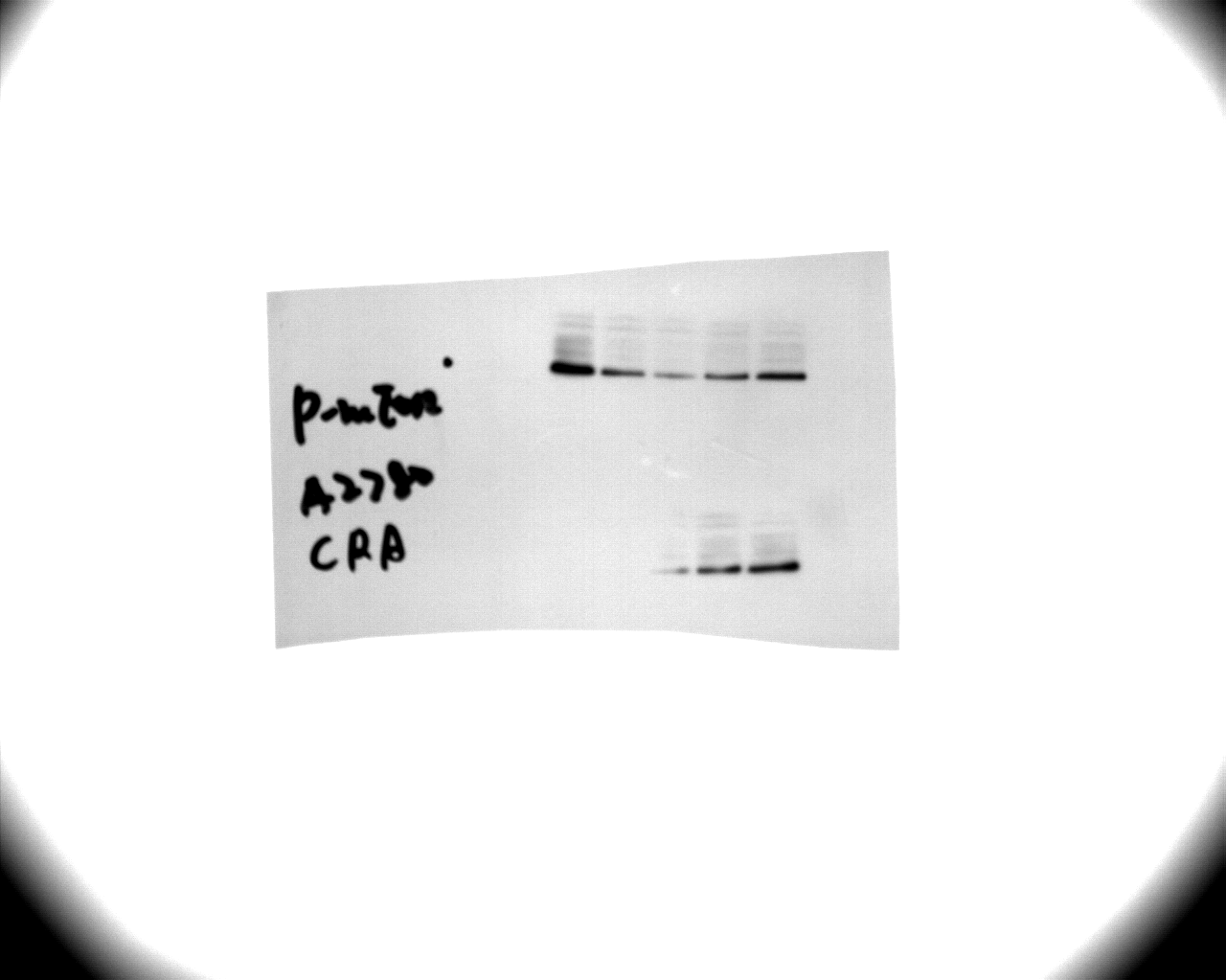

Supplement: S2 File — (ZIP) [file pone.0322733.s006.zip › Original Western Blot Images/Original Western Blot Images/Fig.5B/A2780-p-mTOR.tif]

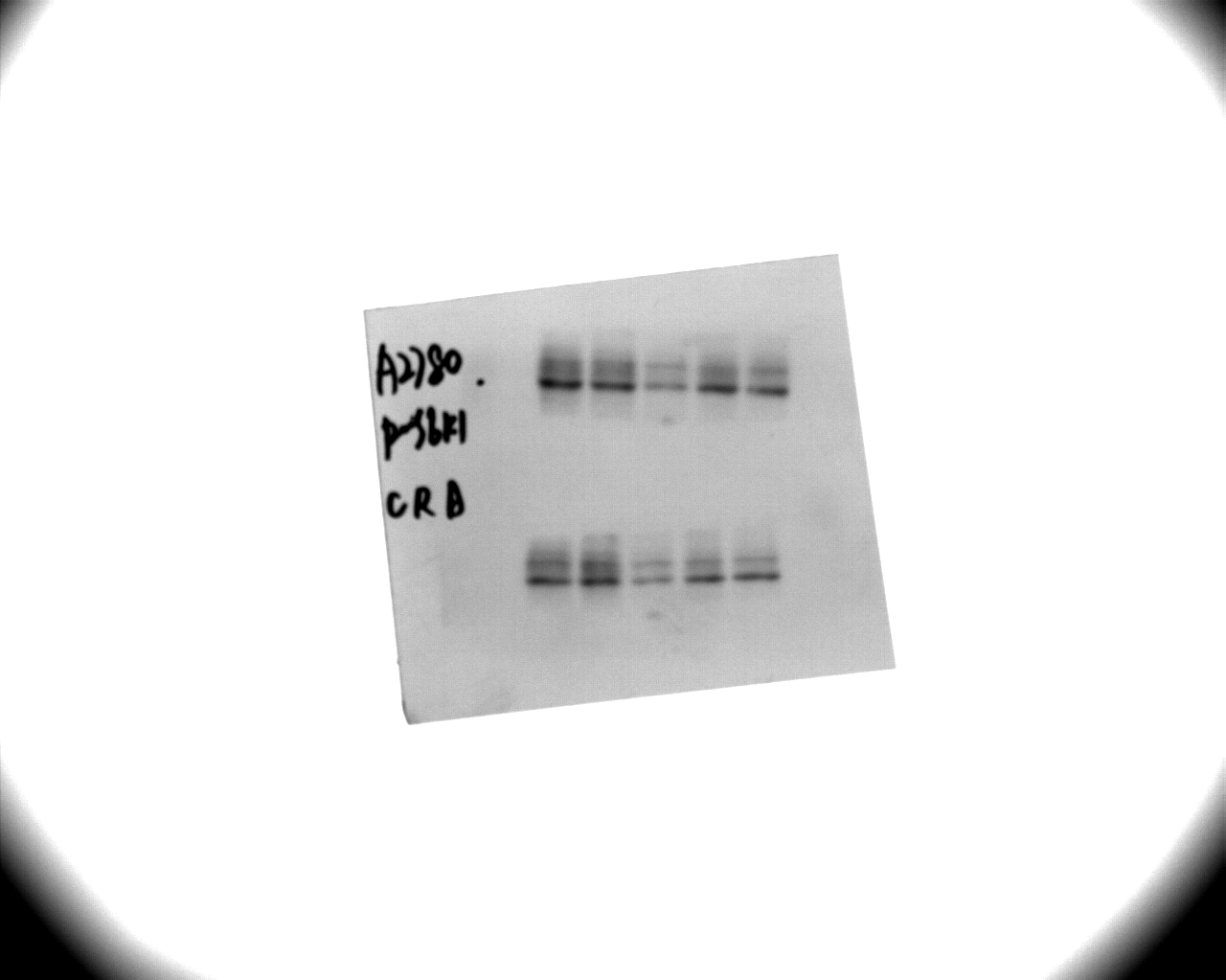

Supplement: S2 File — (ZIP) [file pone.0322733.s006.zip › Original Western Blot Images/Original Western Blot Images/Fig.5B/A2780-p-S6K1.tif]

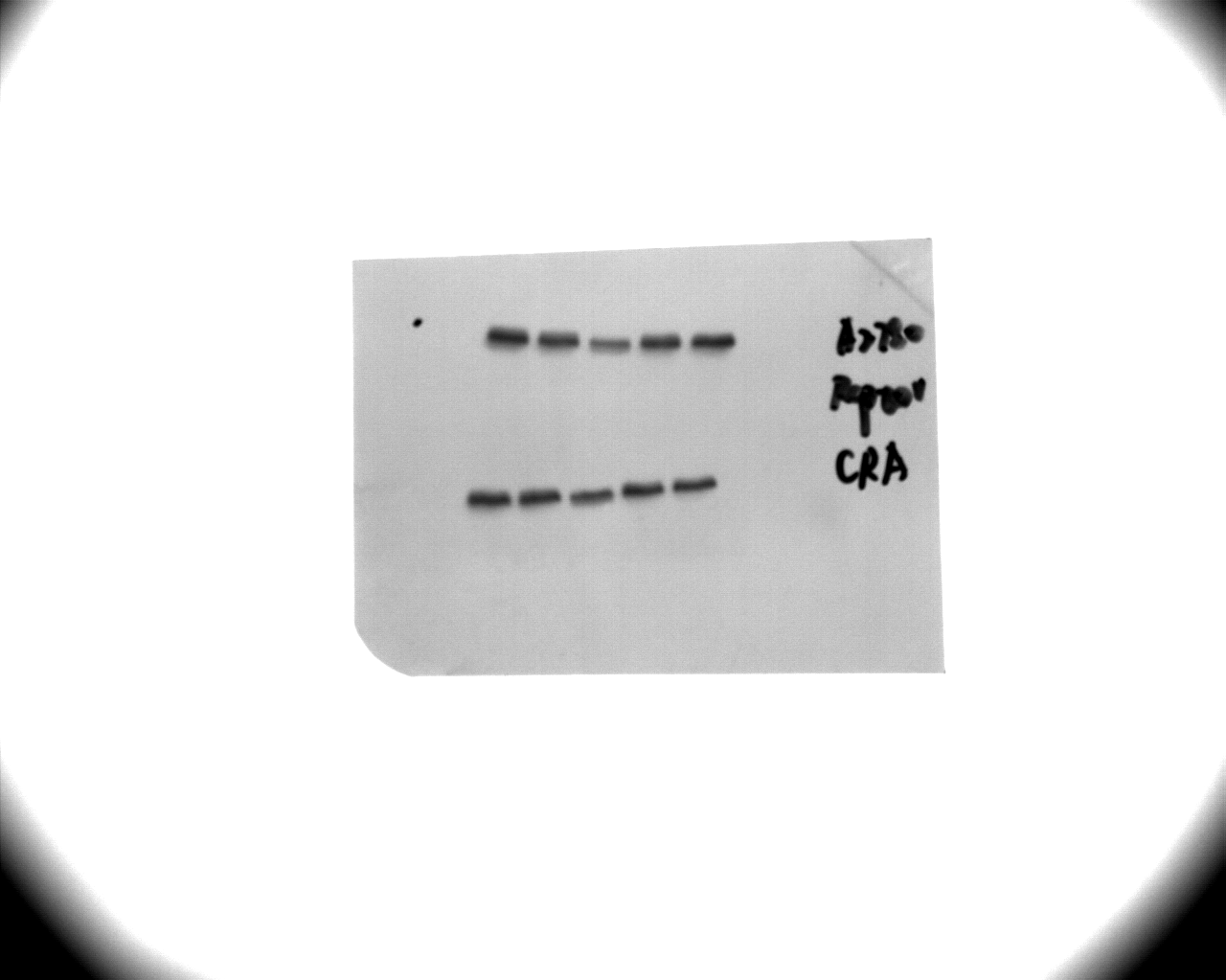

Supplement: S2 File — (ZIP) [file pone.0322733.s006.zip › Original Western Blot Images/Original Western Blot Images/Fig.5B/A2780-Raptor.tif]

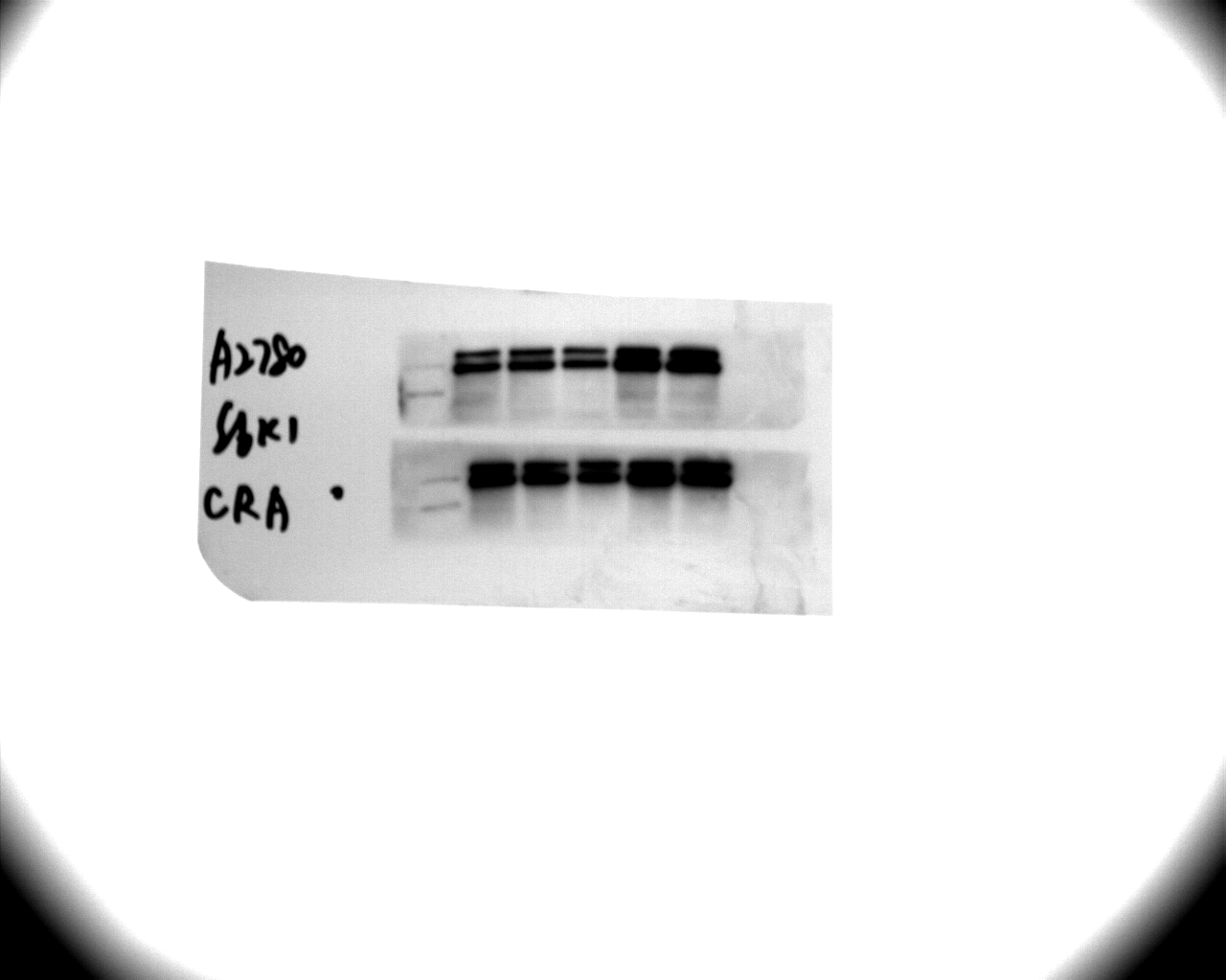

Supplement: S2 File — (ZIP) [file pone.0322733.s006.zip › Original Western Blot Images/Original Western Blot Images/Fig.5B/A2780-S6K1.tif]

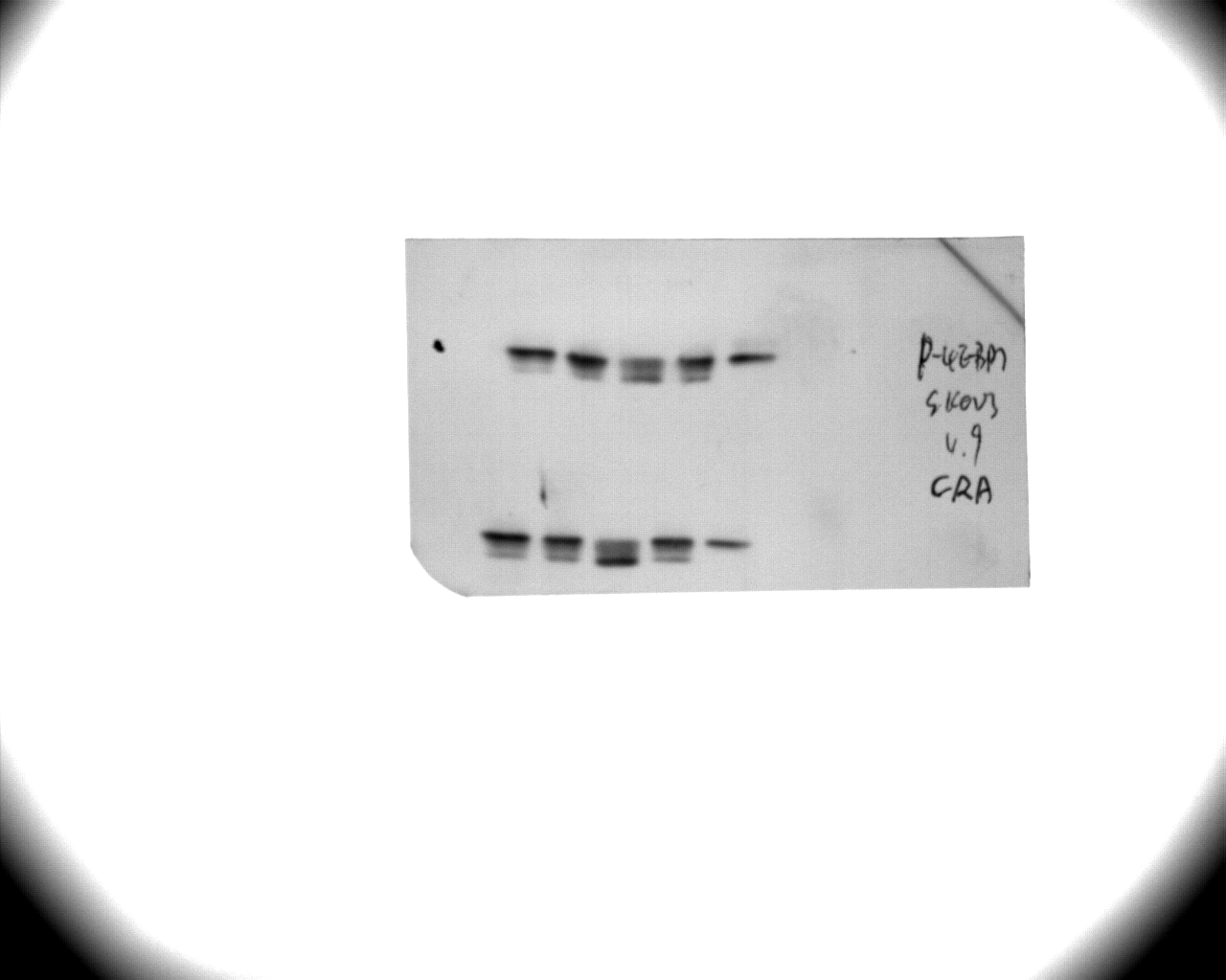

Supplement: S2 File — (ZIP) [file pone.0322733.s006.zip › Original Western Blot Images/Original Western Blot Images/Fig.5A/SKOV3-p-4E-BP1.tif]

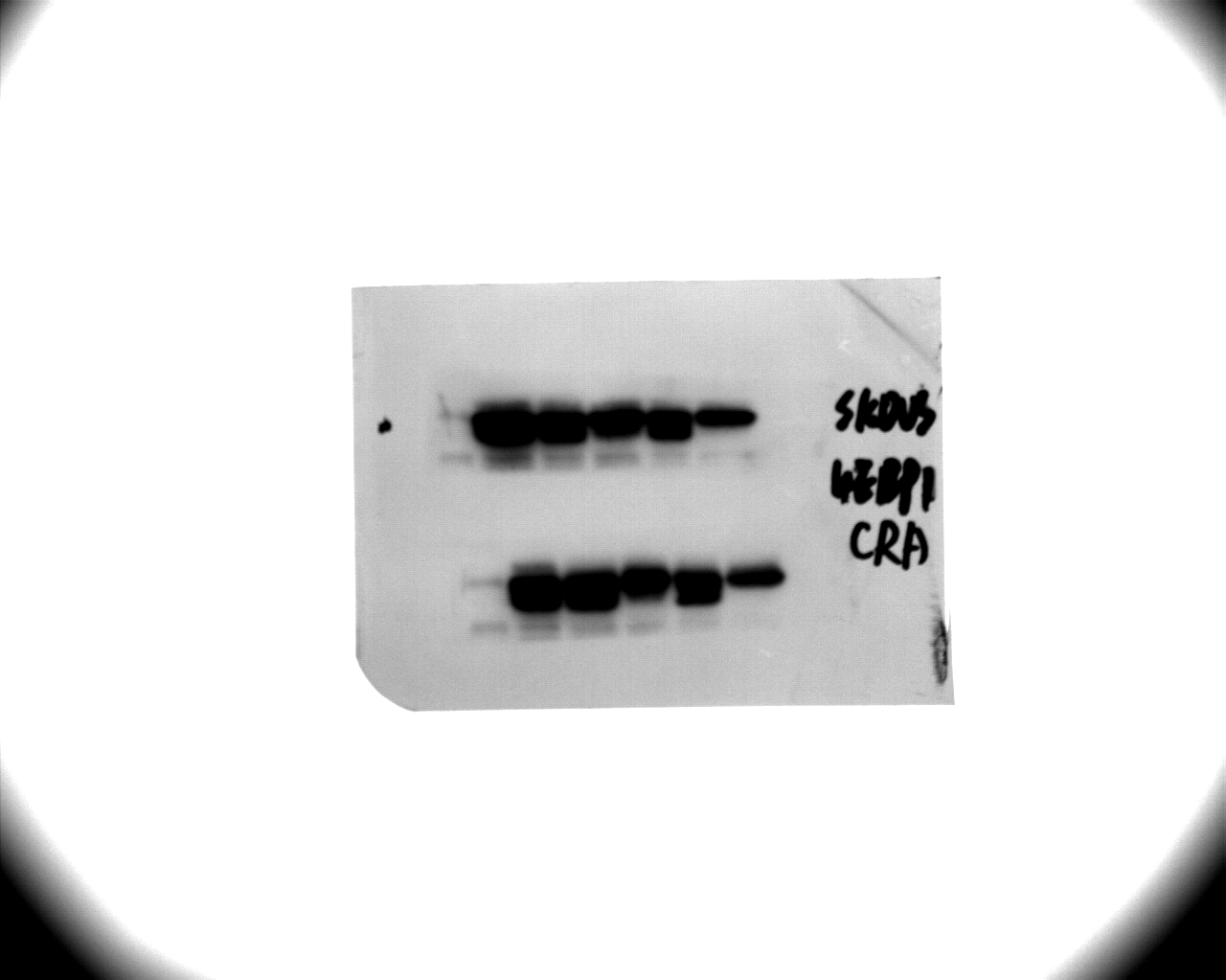

Supplement: S2 File — (ZIP) [file pone.0322733.s006.zip › Original Western Blot Images/Original Western Blot Images/Fig.5A/SKOV3-4E-BP1.tif]

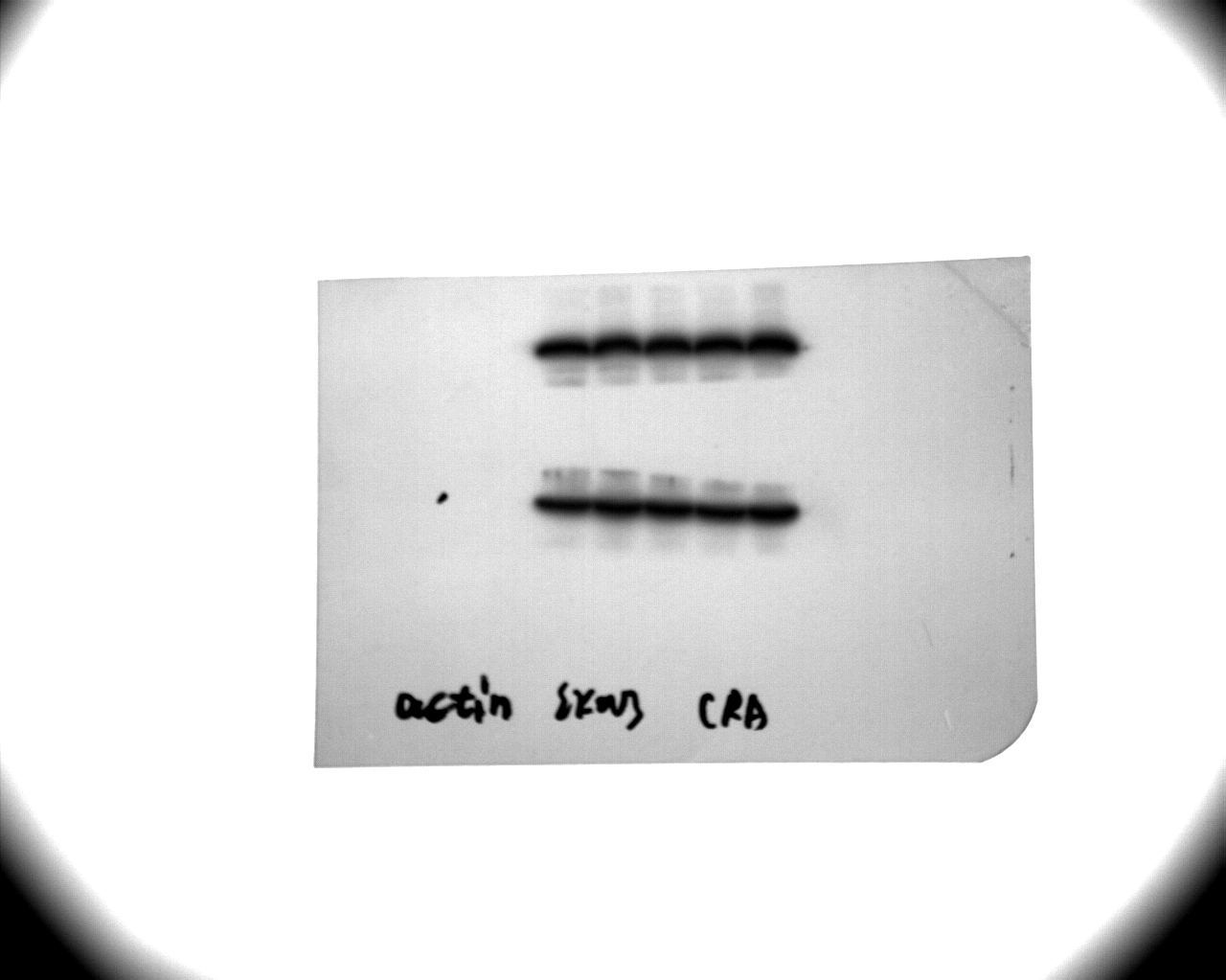

Supplement: S2 File — (ZIP) [file pone.0322733.s006.zip › Original Western Blot Images/Original Western Blot Images/Fig.5A/SKOV3-actin.tif]

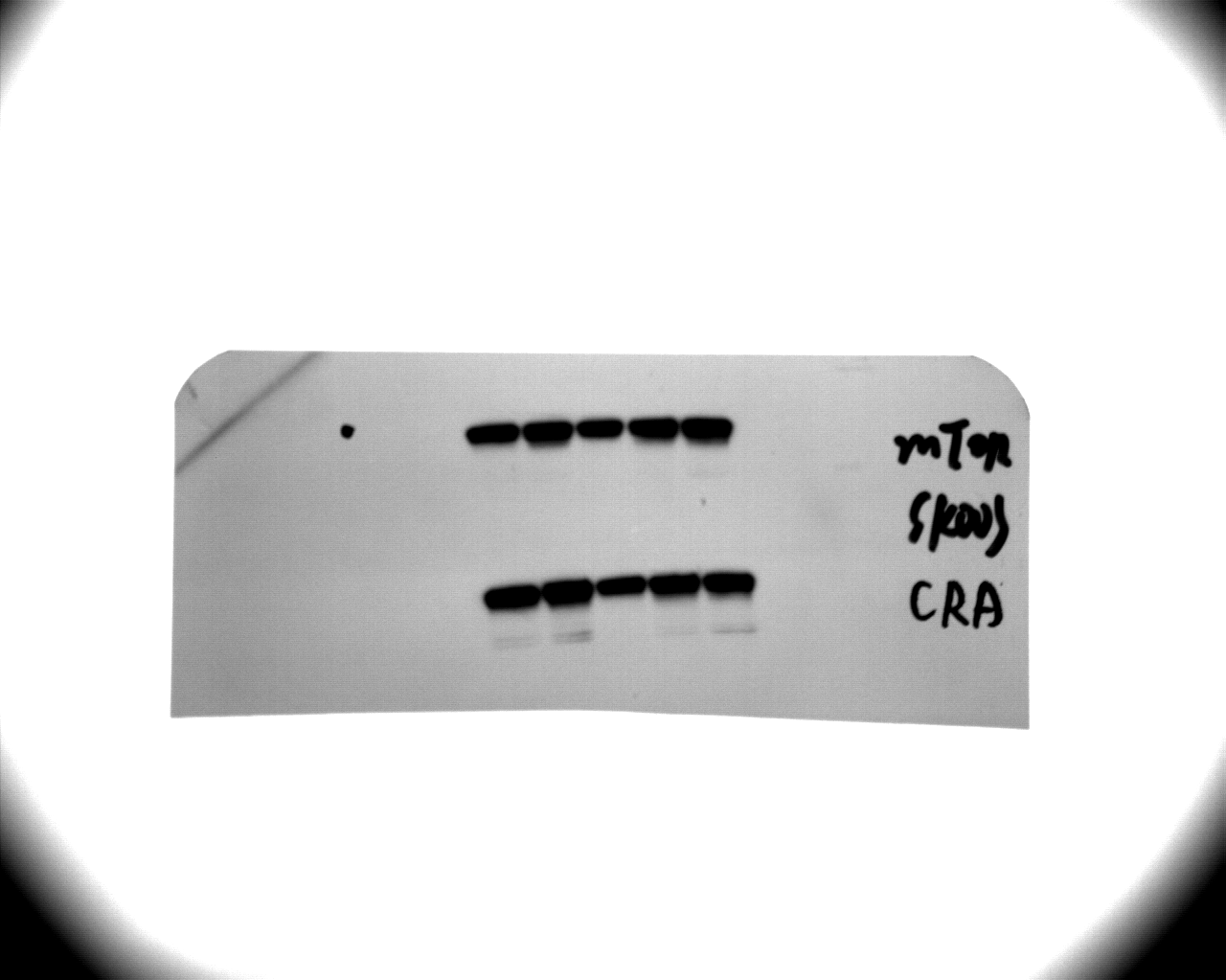

Supplement: S2 File — (ZIP) [file pone.0322733.s006.zip › Original Western Blot Images/Original Western Blot Images/Fig.5A/SKOV3-mTOR.tif]

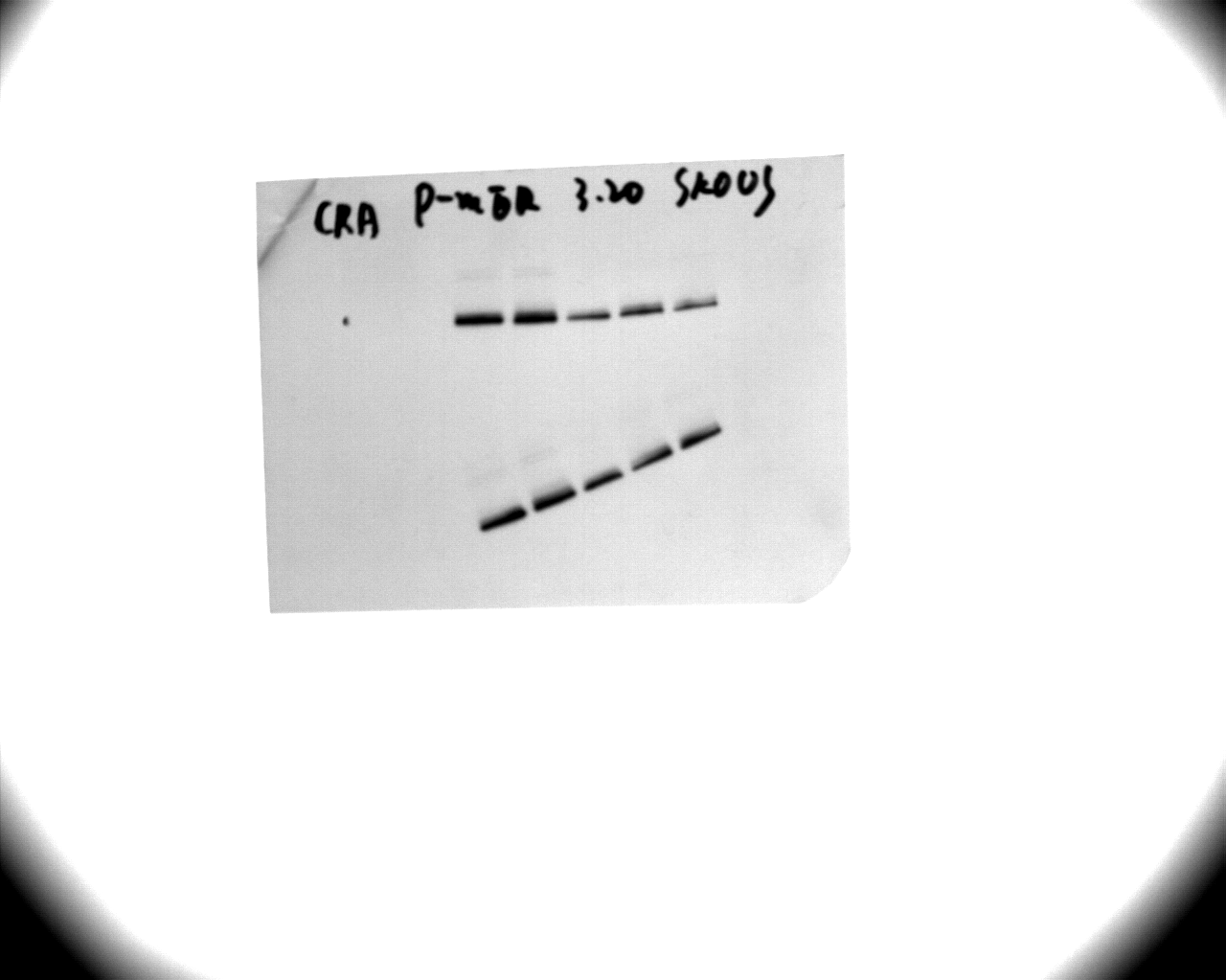

Supplement: S2 File — (ZIP) [file pone.0322733.s006.zip › Original Western Blot Images/Original Western Blot Images/Fig.5A/SKOV3-p-mTOR.tif]

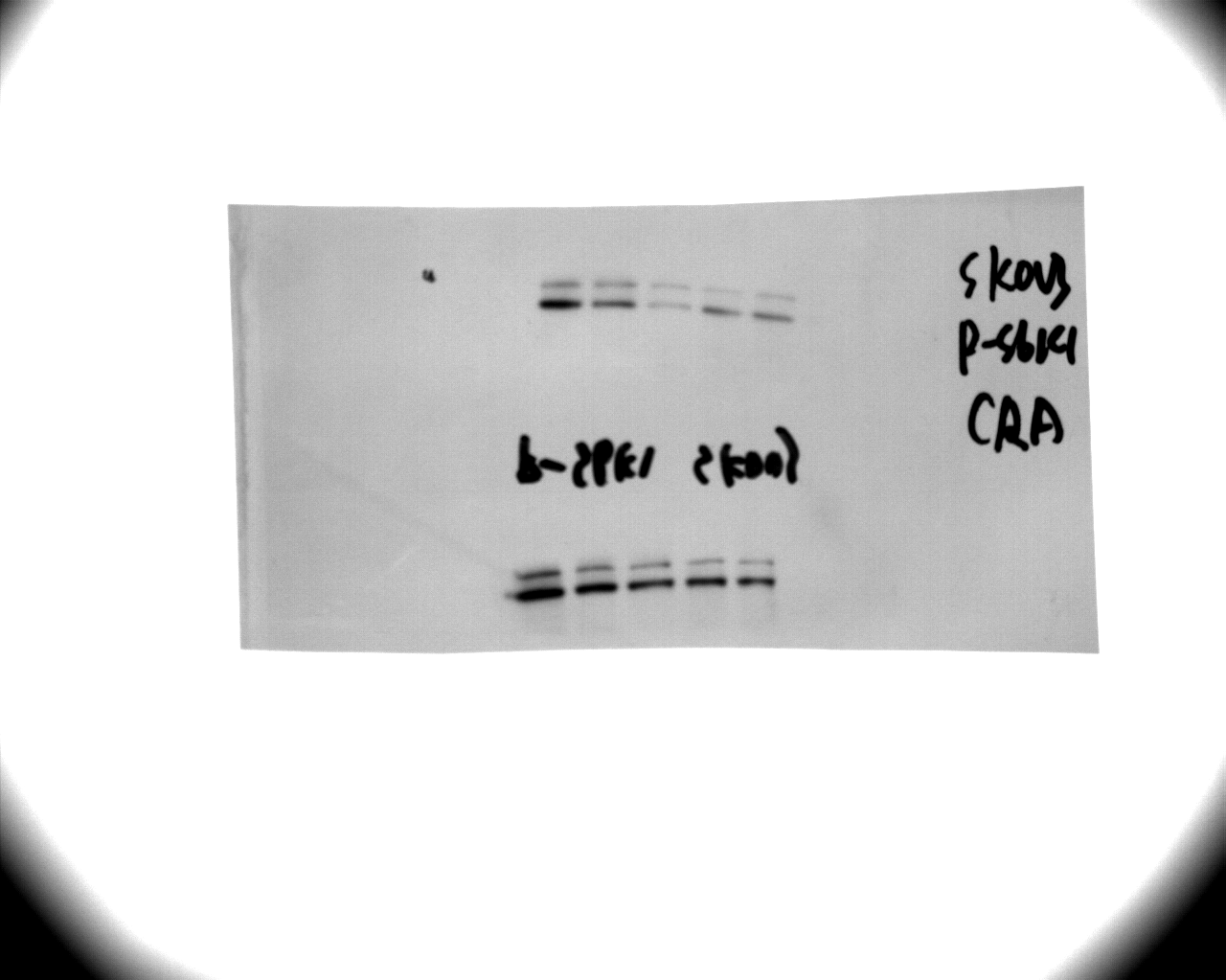

Supplement: S2 File — (ZIP) [file pone.0322733.s006.zip › Original Western Blot Images/Original Western Blot Images/Fig.5A/SKOV3-p-S6K1.tif]

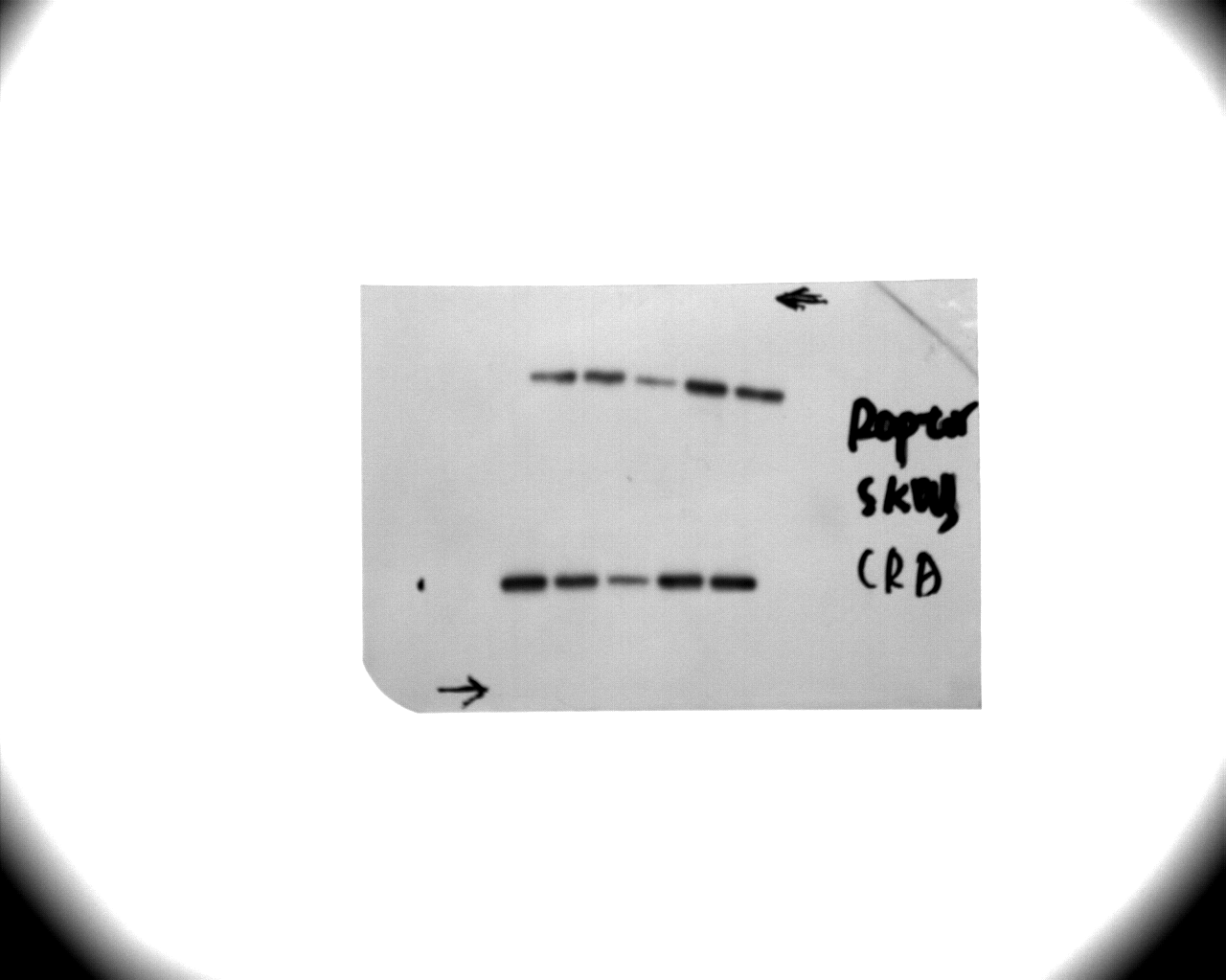

Supplement: S2 File — (ZIP) [file pone.0322733.s006.zip › Original Western Blot Images/Original Western Blot Images/Fig.5A/SKOV3-Raptor.tif]

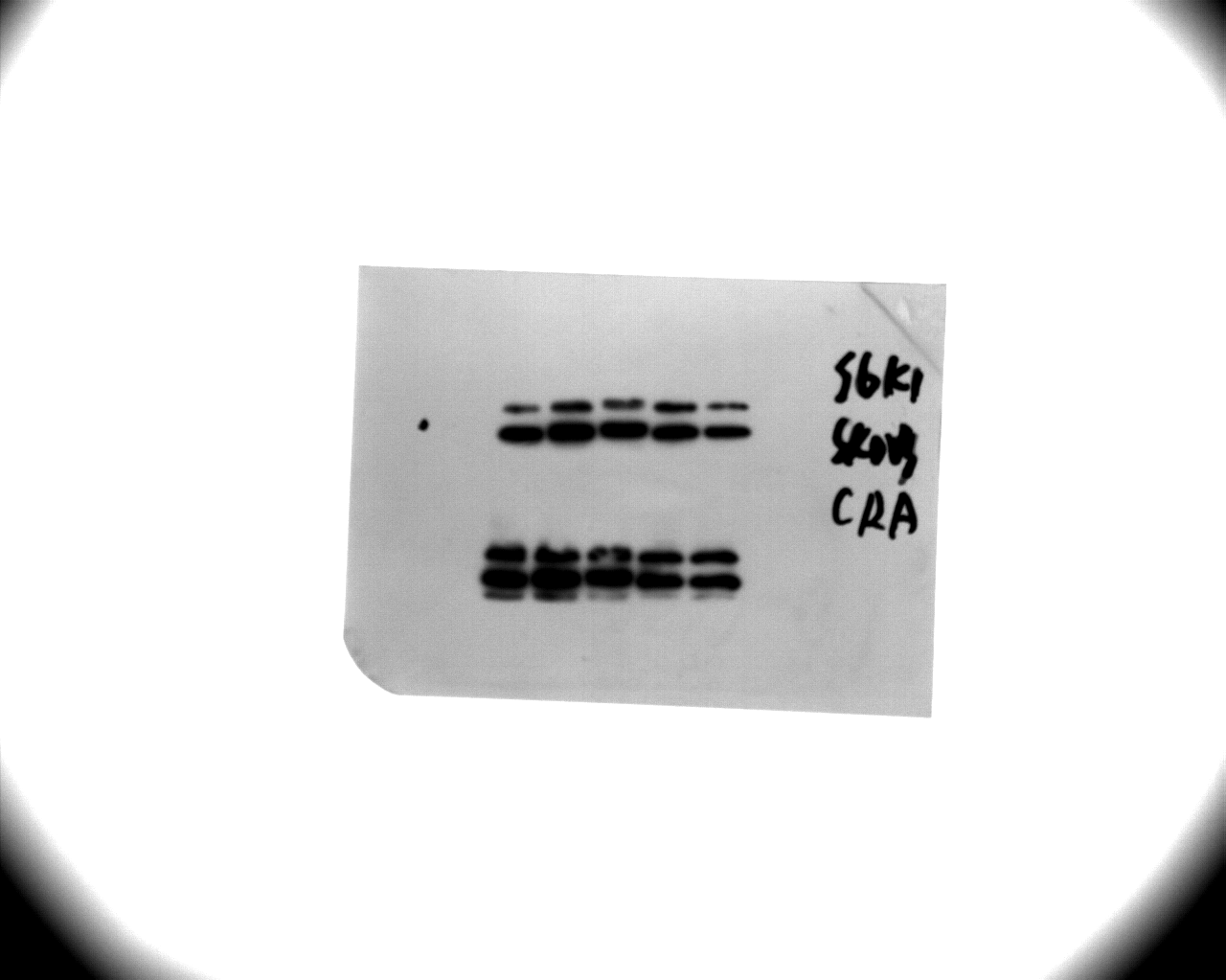

Supplement: S2 File — (ZIP) [file pone.0322733.s006.zip › Original Western Blot Images/Original Western Blot Images/Fig.5A/SKOV3-S6K1.tif]

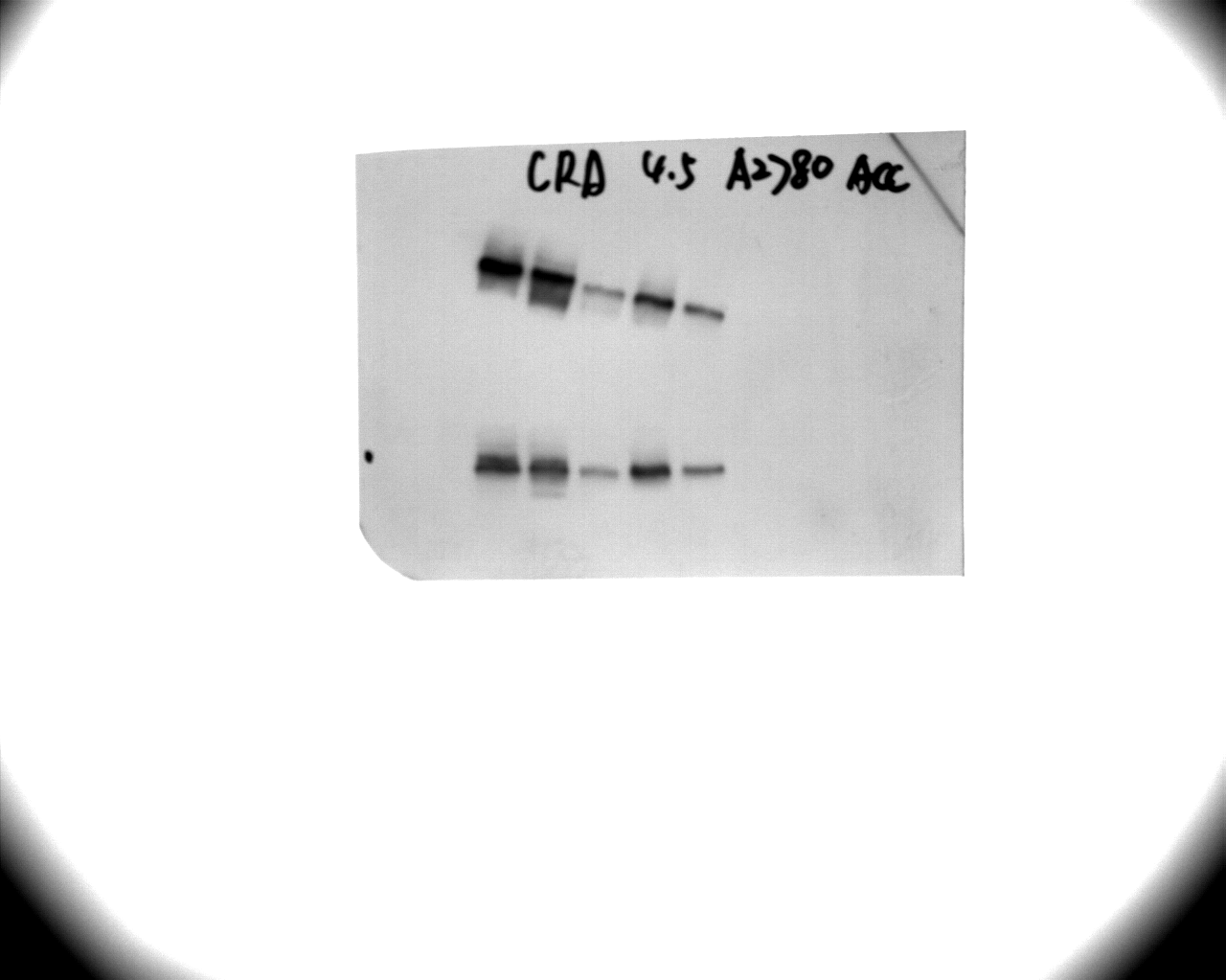

Supplement: S2 File — (ZIP) [file pone.0322733.s006.zip › Original Western Blot Images/Original Western Blot Images/Fig.3D/A2780-ACC.tif]

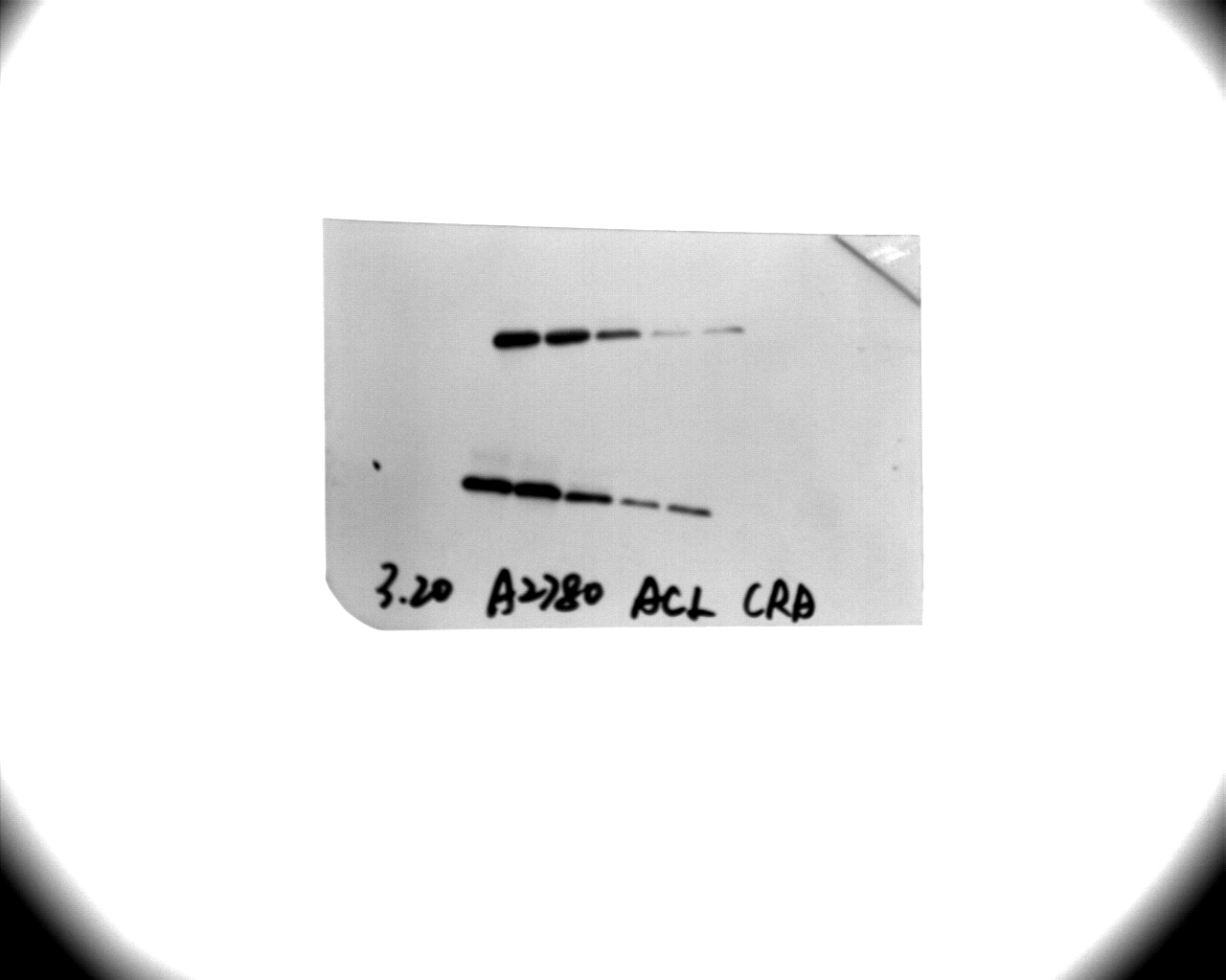

Supplement: S2 File — (ZIP) [file pone.0322733.s006.zip › Original Western Blot Images/Original Western Blot Images/Fig.3D/A2780-ACLY.tif]

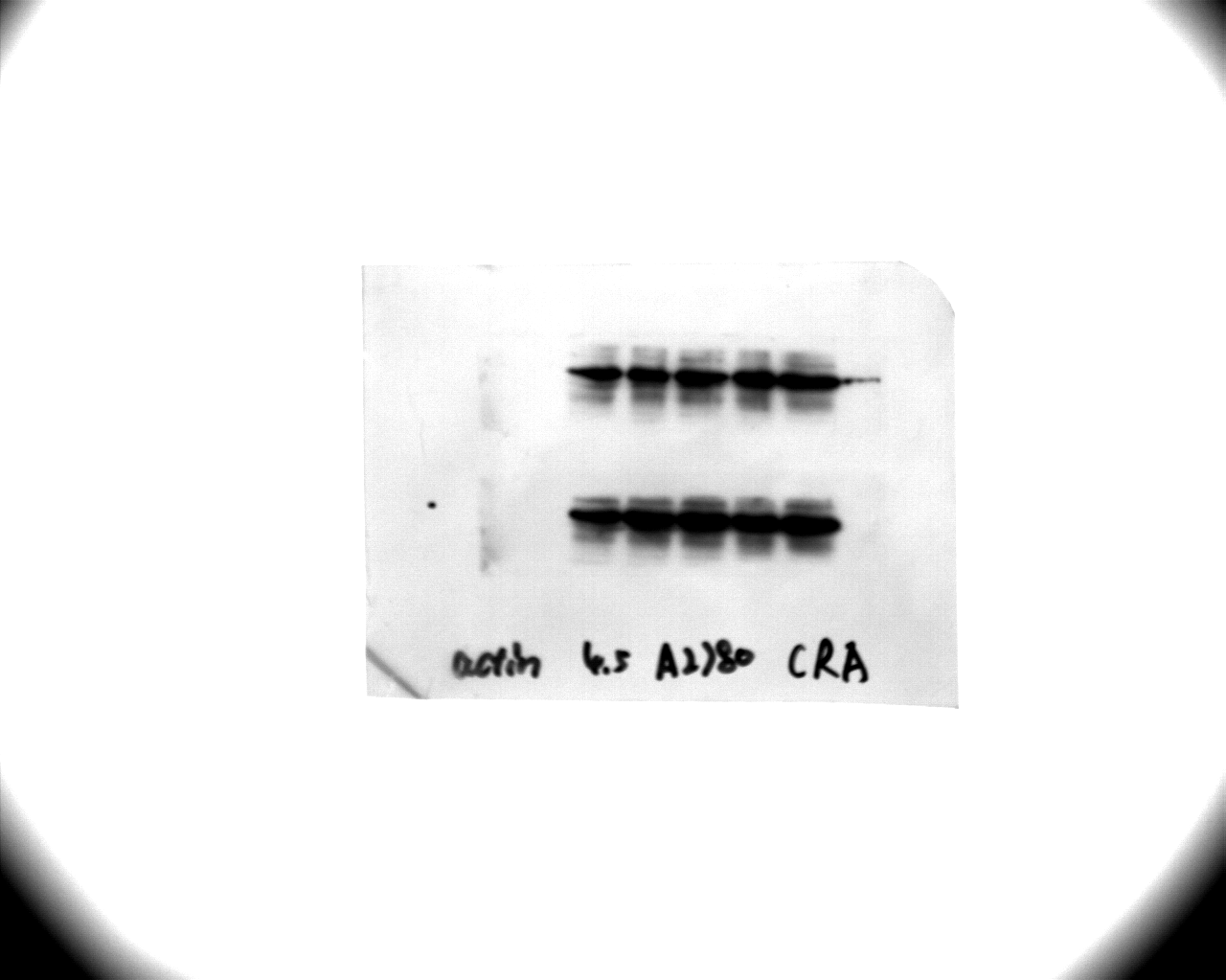

Supplement: S2 File — (ZIP) [file pone.0322733.s006.zip › Original Western Blot Images/Original Western Blot Images/Fig.3D/A2780-actin.tif]

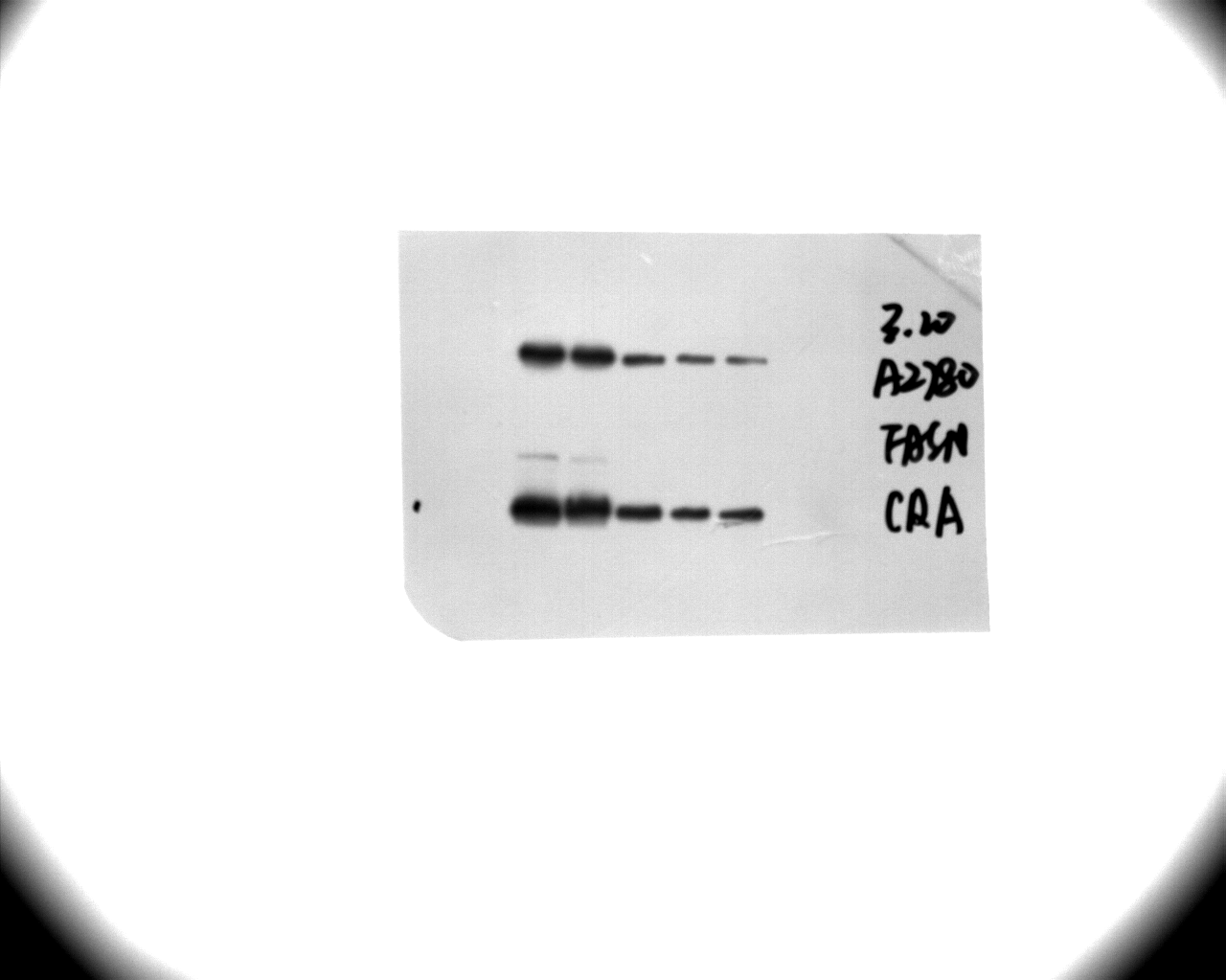

Supplement: S2 File — (ZIP) [file pone.0322733.s006.zip › Original Western Blot Images/Original Western Blot Images/Fig.3D/A2780-FASN.tif]

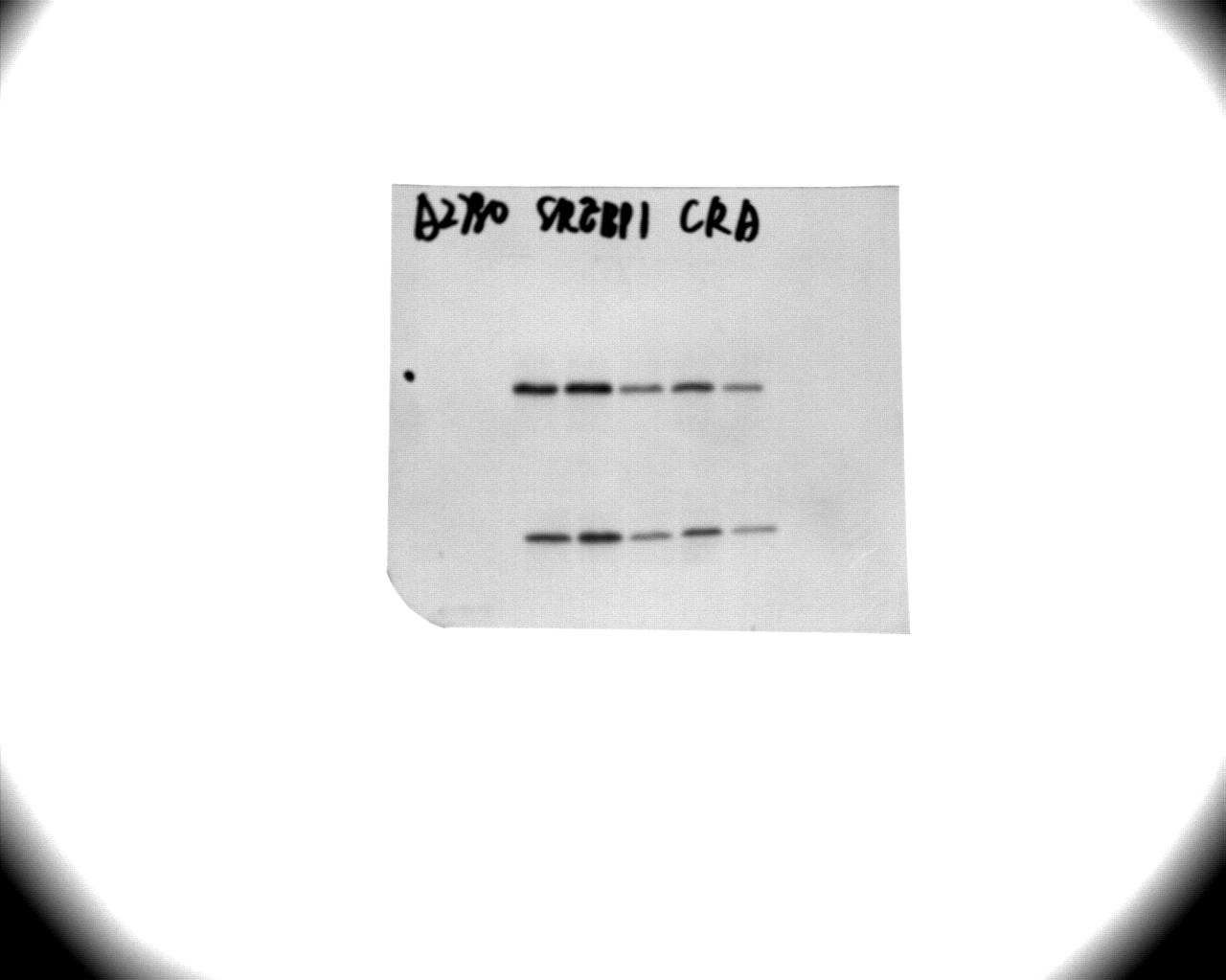

Supplement: S2 File — (ZIP) [file pone.0322733.s006.zip › Original Western Blot Images/Original Western Blot Images/Fig.3D/A2780-SREBP1.tif]

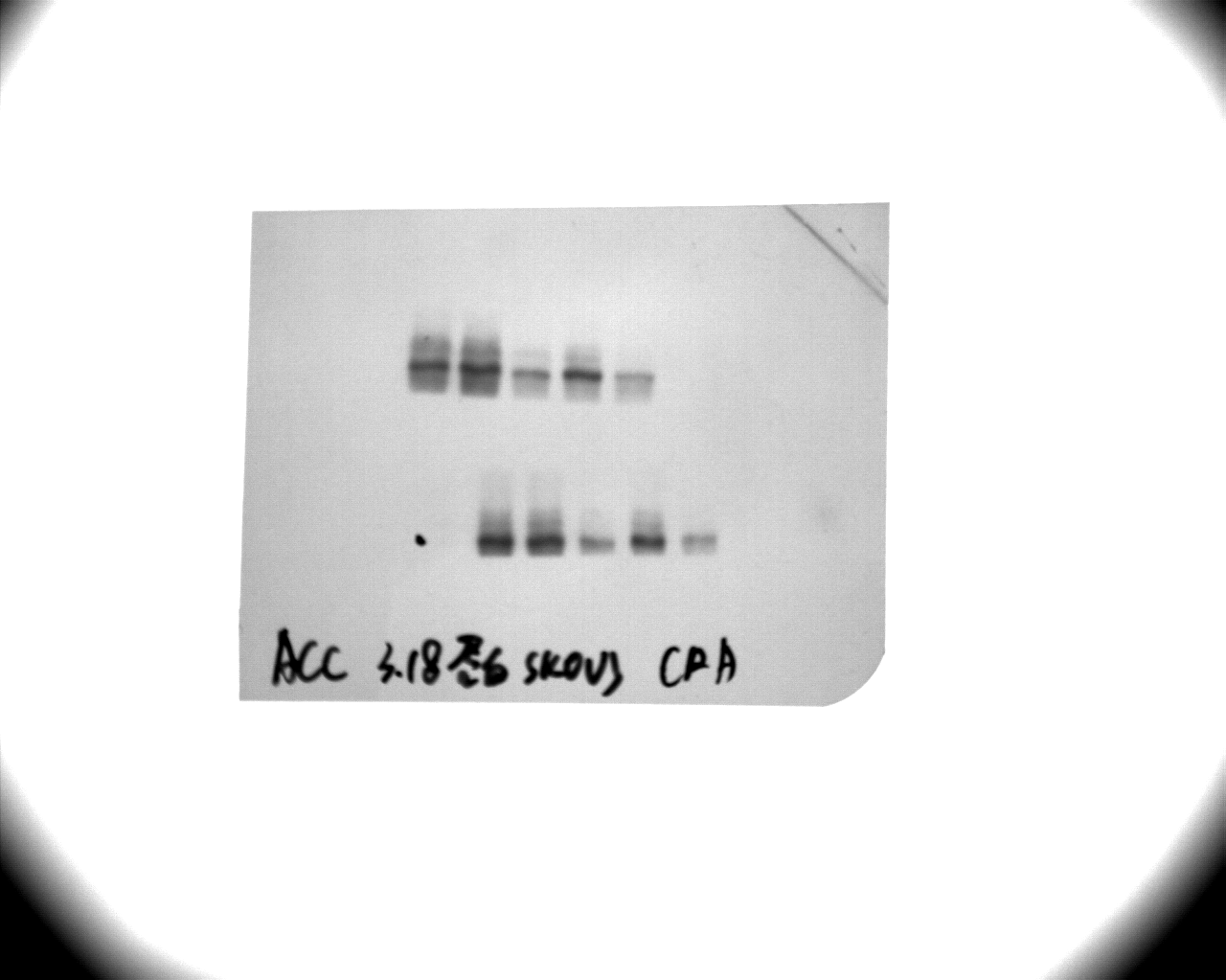

Supplement: S2 File — (ZIP) [file pone.0322733.s006.zip › Original Western Blot Images/Original Western Blot Images/Fig.3C/SKOV3-ACC.tif]

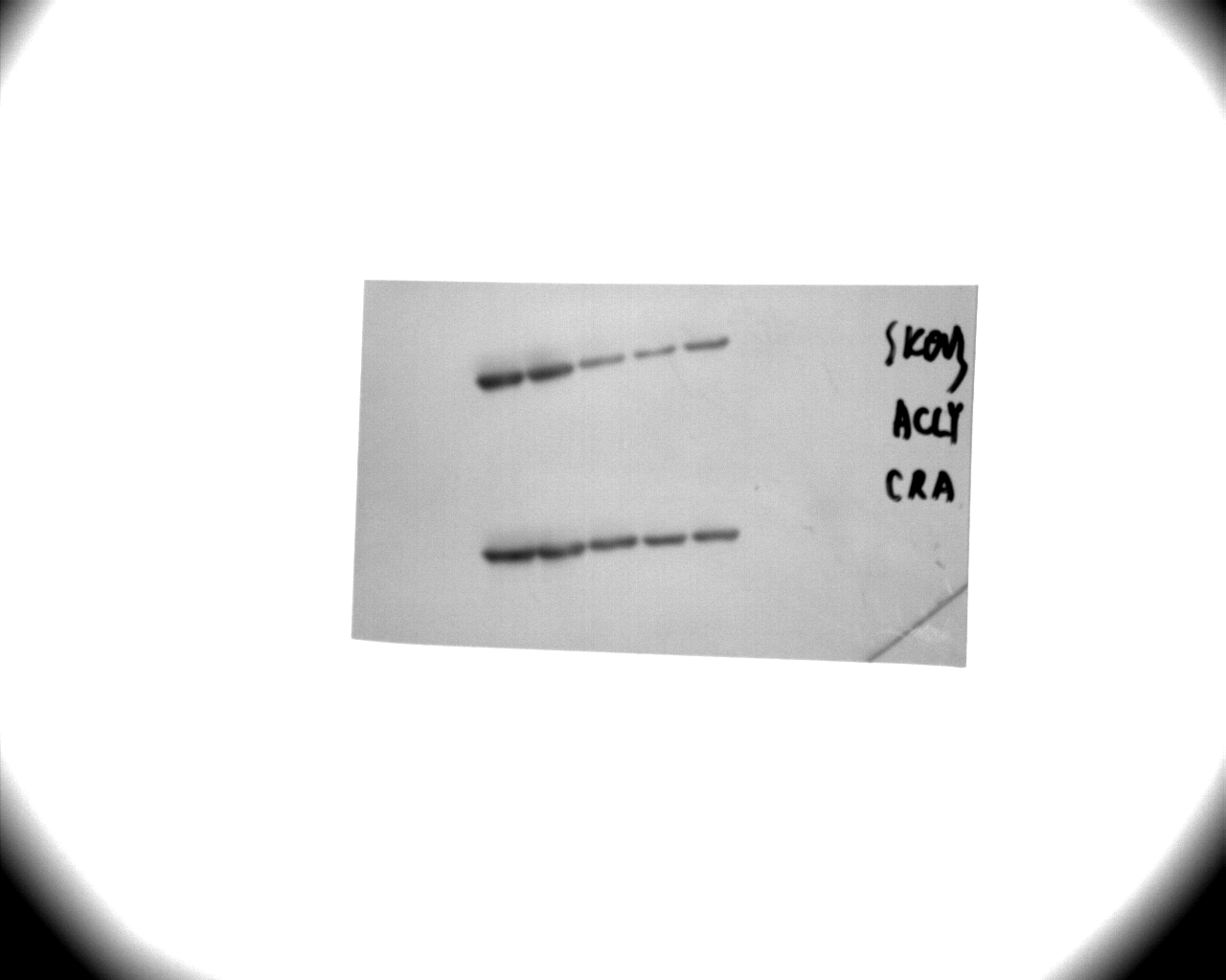

Supplement: S2 File — (ZIP) [file pone.0322733.s006.zip › Original Western Blot Images/Original Western Blot Images/Fig.3C/SKOV3-ACLY.tif]

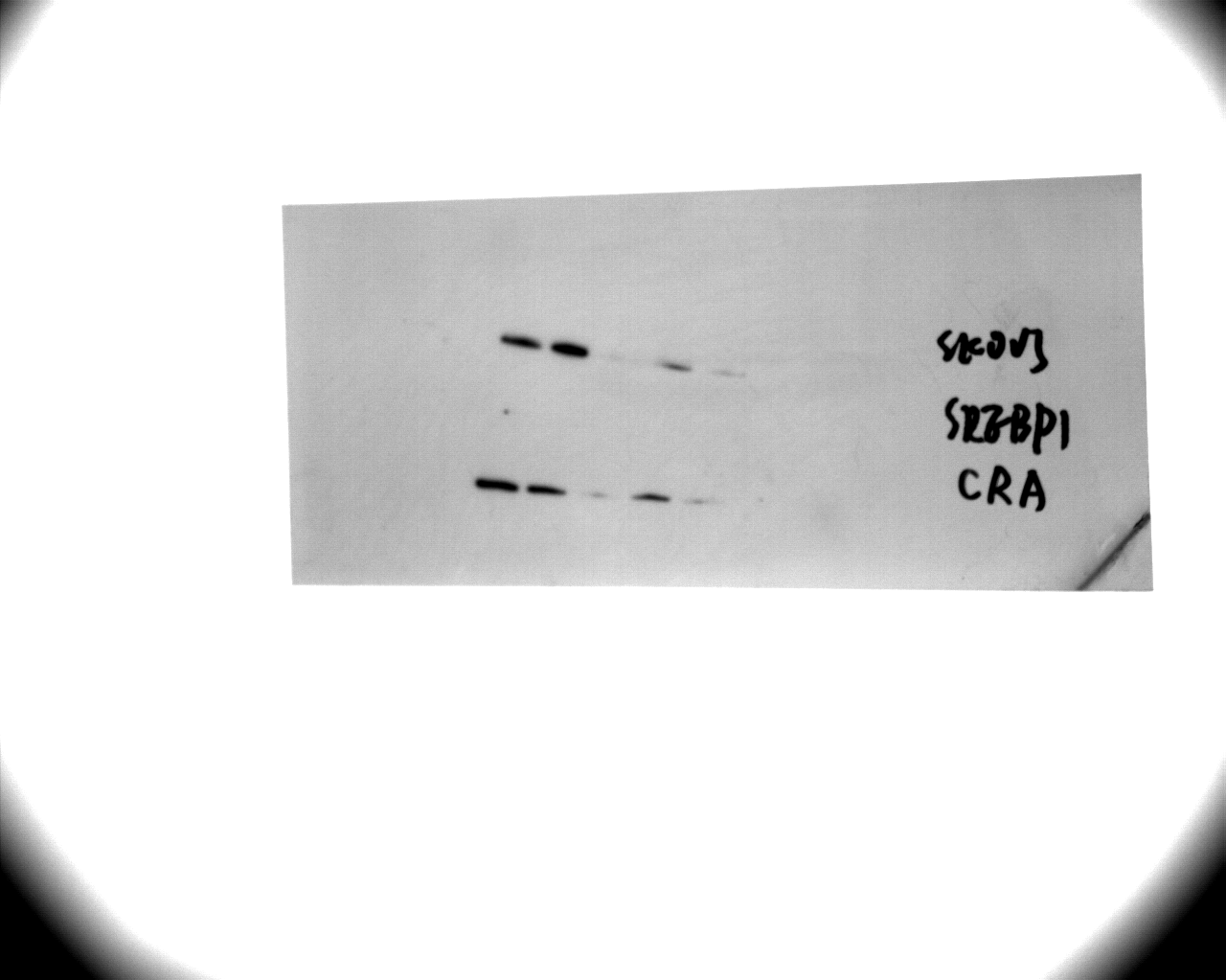

Supplement: S2 File — (ZIP) [file pone.0322733.s006.zip › Original Western Blot Images/Original Western Blot Images/Fig.3C/SKOV3-SREBP1.tif]

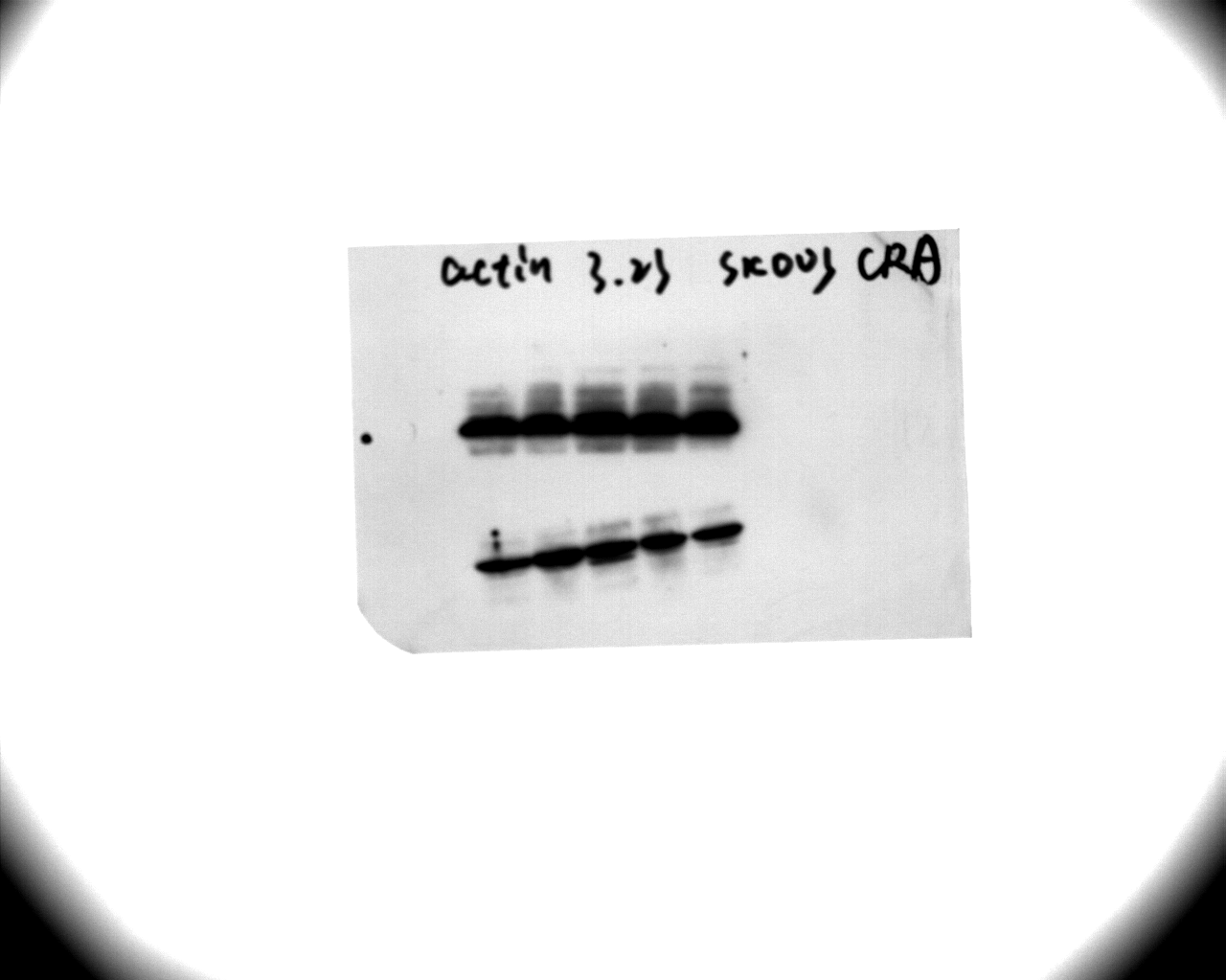

Supplement: S2 File — (ZIP) [file pone.0322733.s006.zip › Original Western Blot Images/Original Western Blot Images/Fig.3C/SKOV3-actin.tif]

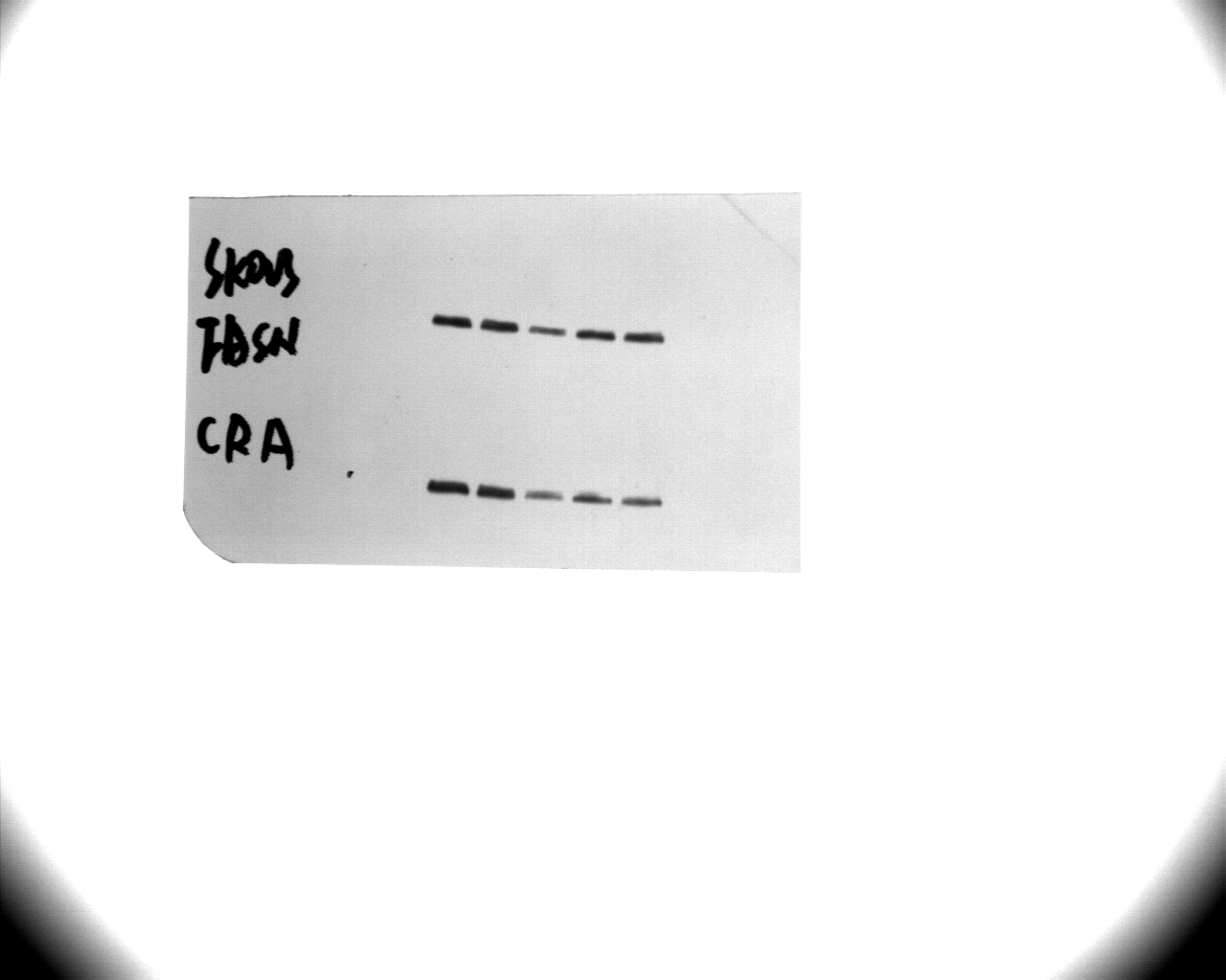

Supplement: S2 File — (ZIP) [file pone.0322733.s006.zip › Original Western Blot Images/Original Western Blot Images/Fig.3C/SKOV3-FASN.tif]

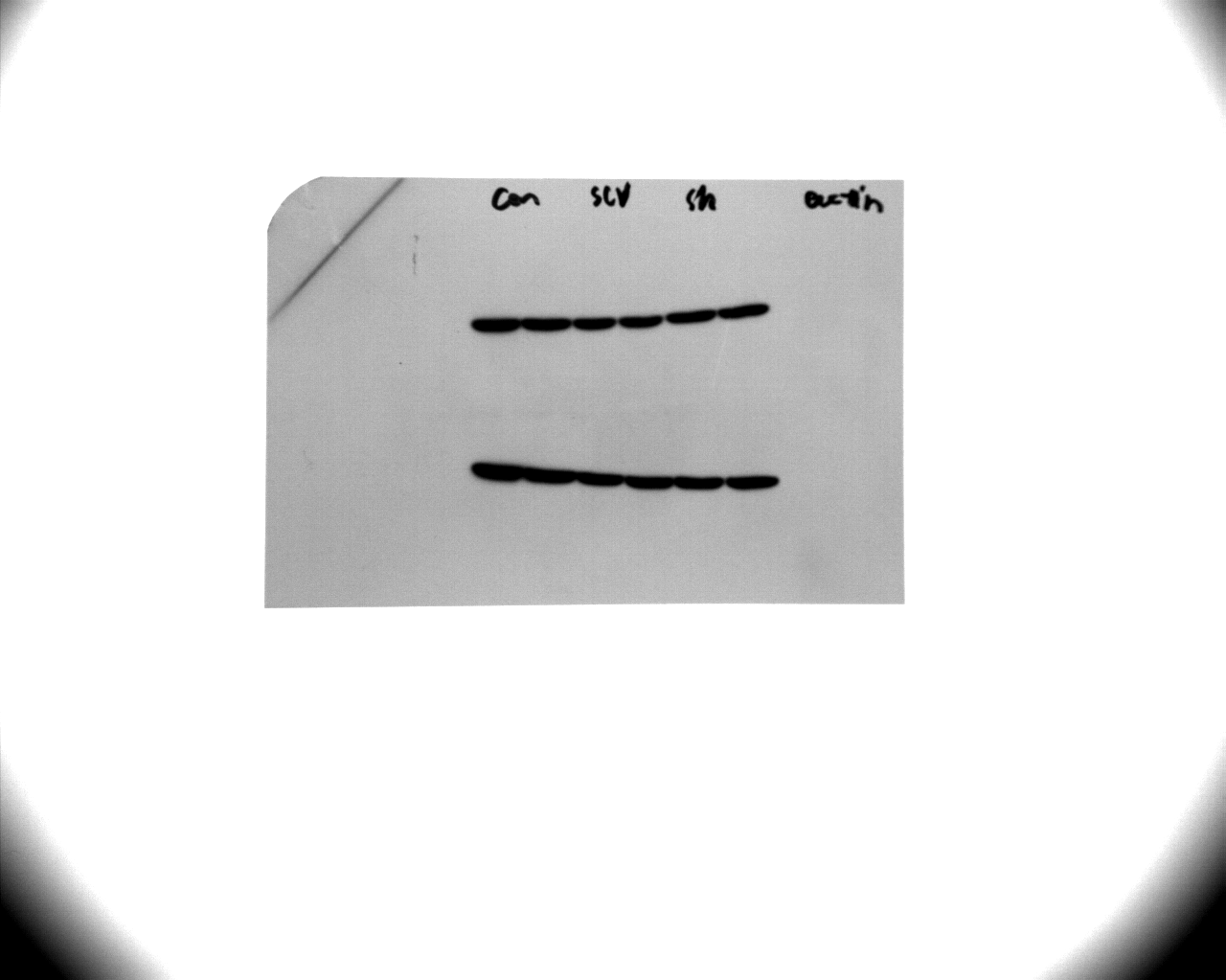

Supplement: S2 File — (ZIP) [file pone.0322733.s006.zip › Original Western Blot Images/Original Western Blot Images/Fig.6A/shRNA-Raptor actin.tif]

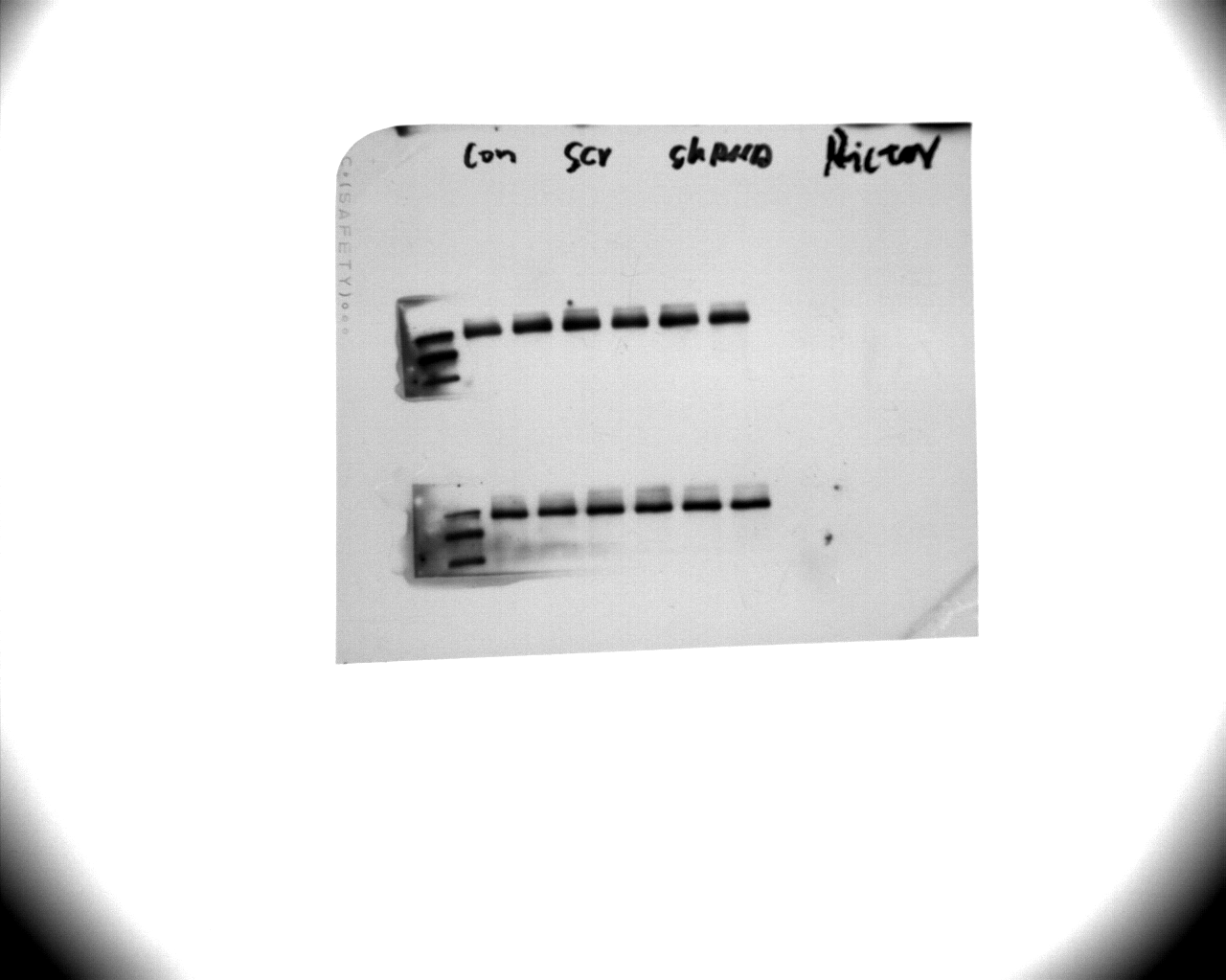

Supplement: S2 File — (ZIP) [file pone.0322733.s006.zip › Original Western Blot Images/Original Western Blot Images/Fig.6A/shRNA-Raptor Rictor.tif]

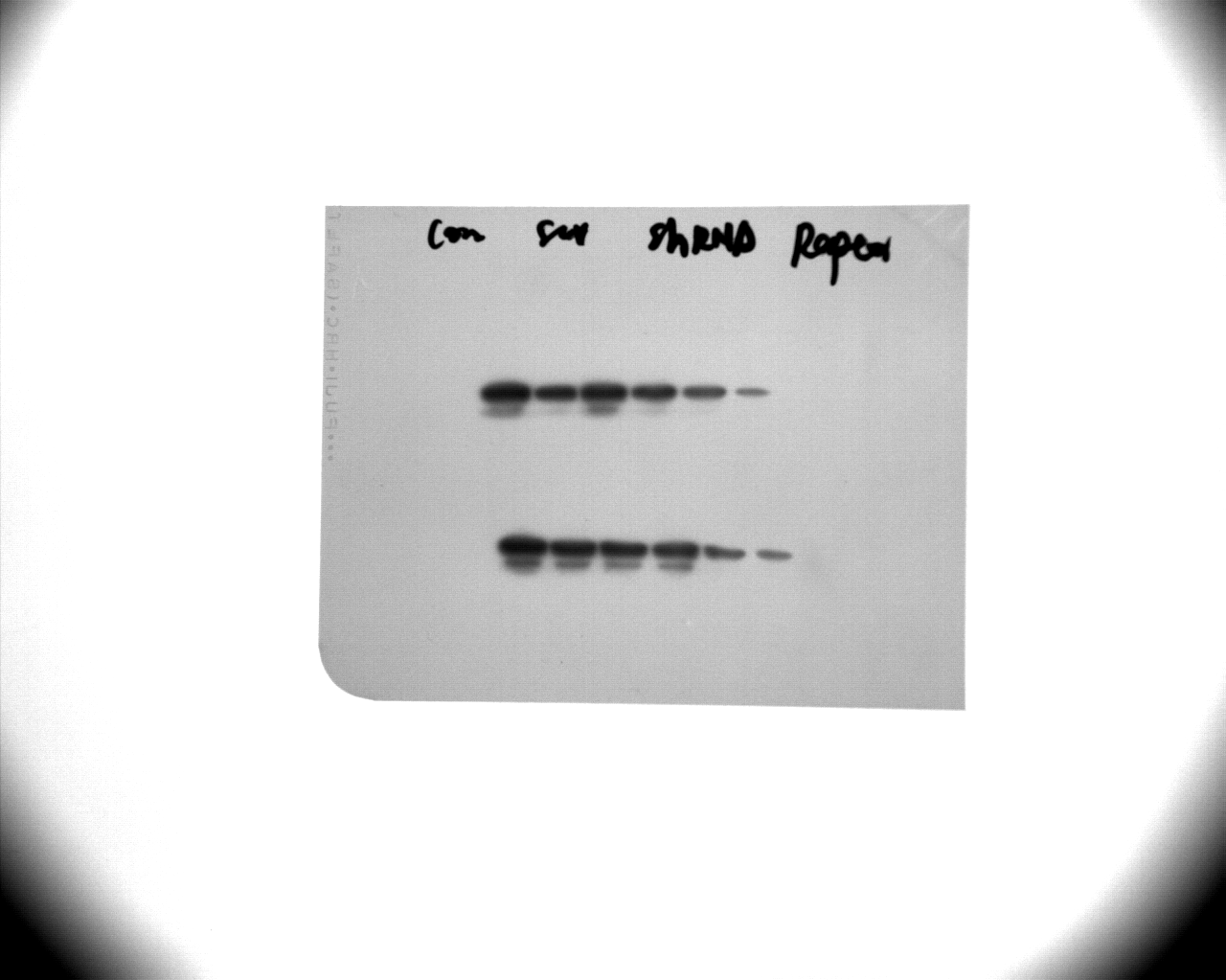

Supplement: S2 File — (ZIP) [file pone.0322733.s006.zip › Original Western Blot Images/Original Western Blot Images/Fig.6A/shRNA-Raptor Raptor.tif]
