# Supplementary material for: Cardamonin suppresses mTORC1/SREBP1 through reducing Raptor and inhibits de novo lipogenesis in ovarian cancer
Source: PLoS One. 2025 May 2;20(5):e0322733. doi: 10.1371/journal.pone.0322733 (PMC12047825; doi:10.1371/journal.pone.0322733)
Supplement: S2 File — (ZIP) [file pone.0322733.s006.zip › Original Western Blot Images/Original Western Blot Images/Fig.7C/Original Western Blot Images (For Fig.7C).docx]

Original western blot images for Fig 7C.

The protein blots are imaged by X-ray film exposure. The blots which marked with red frame are used for figure preparation.

Fig 7C


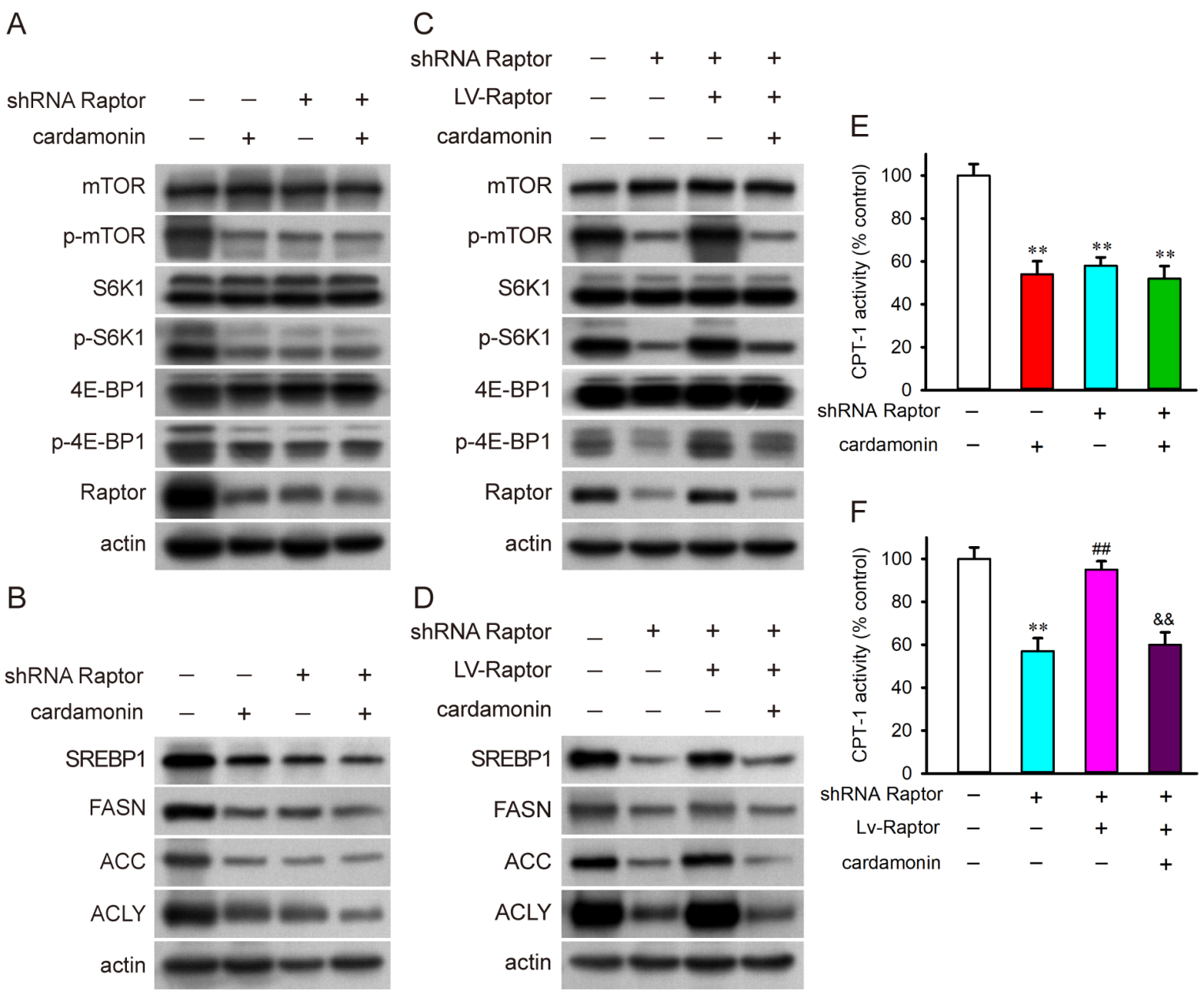





Fig 7C mTOR





Fig 7C p-mTOR





Fig 7C S6K1





Fig 7C p-S6K1





Fig 7C 4E-BP1





Fig 7C p-4E-BP1





Fig 7C Raptor





Fig 7C actin
