# Supplementary material for: Cardamonin suppresses mTORC1/SREBP1 through reducing Raptor and inhibits de novo lipogenesis in ovarian cancer
Source: PLoS One. 2025 May 2;20(5):e0322733. doi: 10.1371/journal.pone.0322733 (PMC12047825; doi:10.1371/journal.pone.0322733)
Supplement: S2 File — (ZIP) [file pone.0322733.s006.zip › Original Western Blot Images/Original Western Blot Images/Fig.7B/Original Western Blot Images (For Fig.7B).docx]

Original western blot images for Fig 7B.

The protein blots are imaged by X-ray film exposure. The blots which marked with red frame are used for figure preparation.

Fig 7B


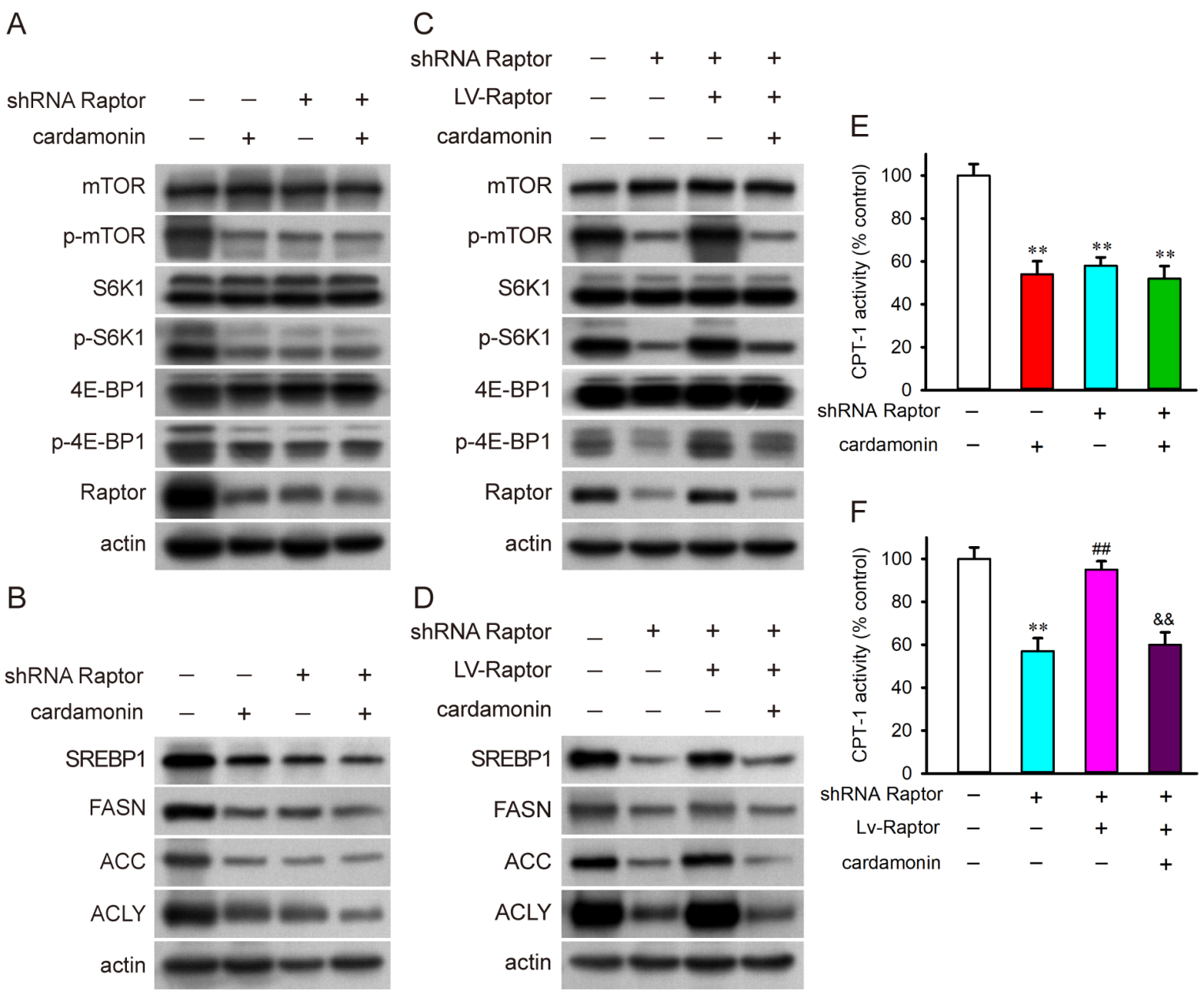





Fig 7B SREBP1





Fig 7B FASN





Fig 7B ACC





Fig 7B ACLY





Fig 7C actin
