# Supplementary material for: Cardamonin suppresses mTORC1/SREBP1 through reducing Raptor and inhibits de novo lipogenesis in ovarian cancer
Source: PLoS One. 2025 May 2;20(5):e0322733. doi: 10.1371/journal.pone.0322733 (PMC12047825; doi:10.1371/journal.pone.0322733)
Supplement: S2 File — (ZIP) [file pone.0322733.s006.zip › Original Western Blot Images/Original Western Blot Images/Fig.7A/Original Western Blot Images (For Fig.7A).docx]

Original western blot images for Fig 7A.

The protein blots are imaged by X-ray film exposure. The blots which marked with red frame are used for figure preparation.

Fig 7A


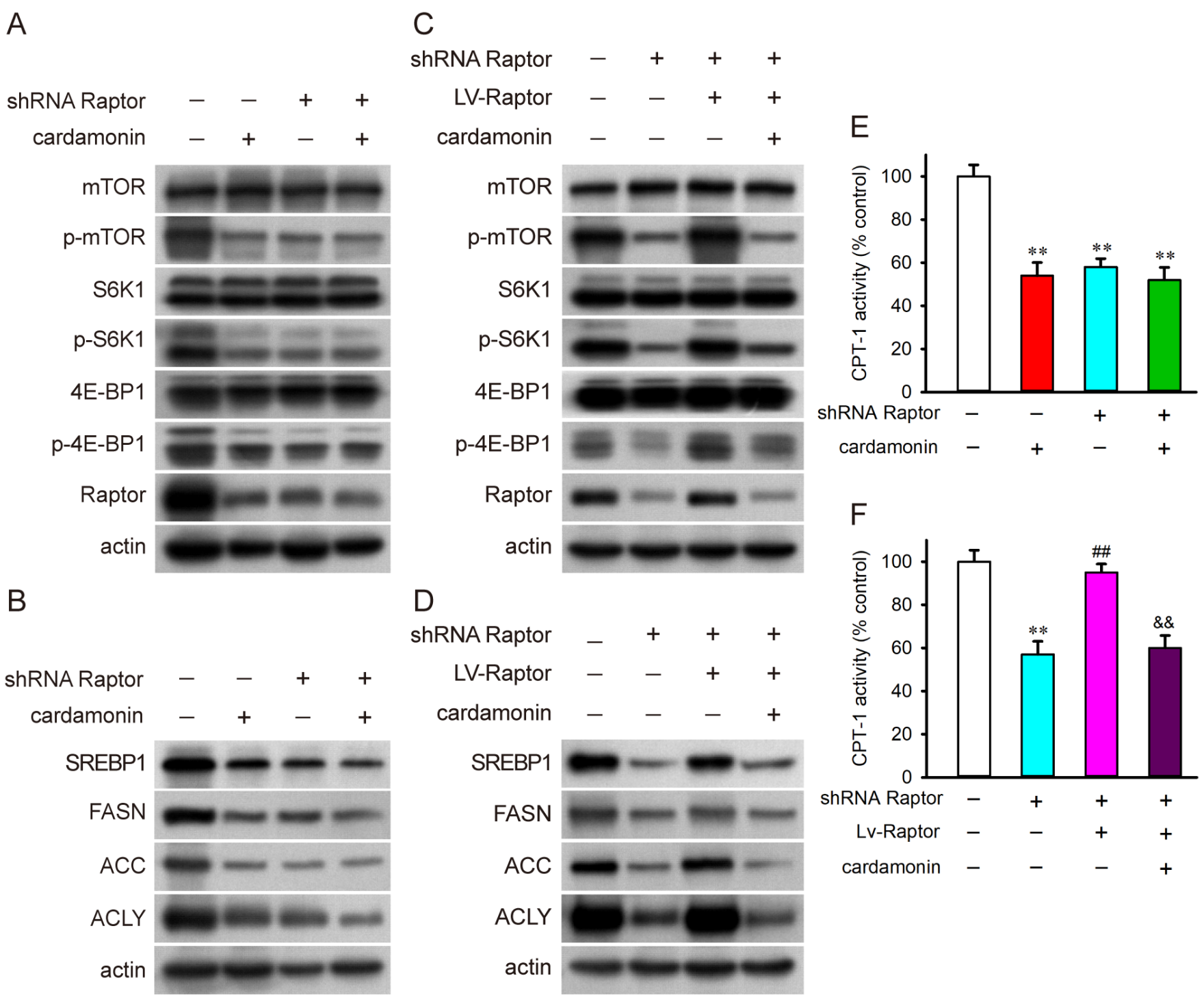





Fig 7A mTOR





Fig 7A p-mTOR





Fig 7A S6K1





Fig 7A p-S6K1





Fig 7A 4E-BP1





Fig 7A p-4E-BP1





Fig 7A Raptor





Fig 7A actin
