# Supplementary material for: Cardamonin suppresses mTORC1/SREBP1 through reducing Raptor and inhibits de novo lipogenesis in ovarian cancer
Source: PLoS One. 2025 May 2;20(5):e0322733. doi: 10.1371/journal.pone.0322733 (PMC12047825; doi:10.1371/journal.pone.0322733)
Supplement: S2 File — (ZIP) [file pone.0322733.s006.zip › Original Western Blot Images/Original Western Blot Images/Fig.5A/Original Western Blot Images (For Fig.5A).docx]

Original western blot images for Fig 5A.

The protein blots are imaged by X-ray film exposure. The blots which marked with red frame are used for figure preparation.

Fig 5A


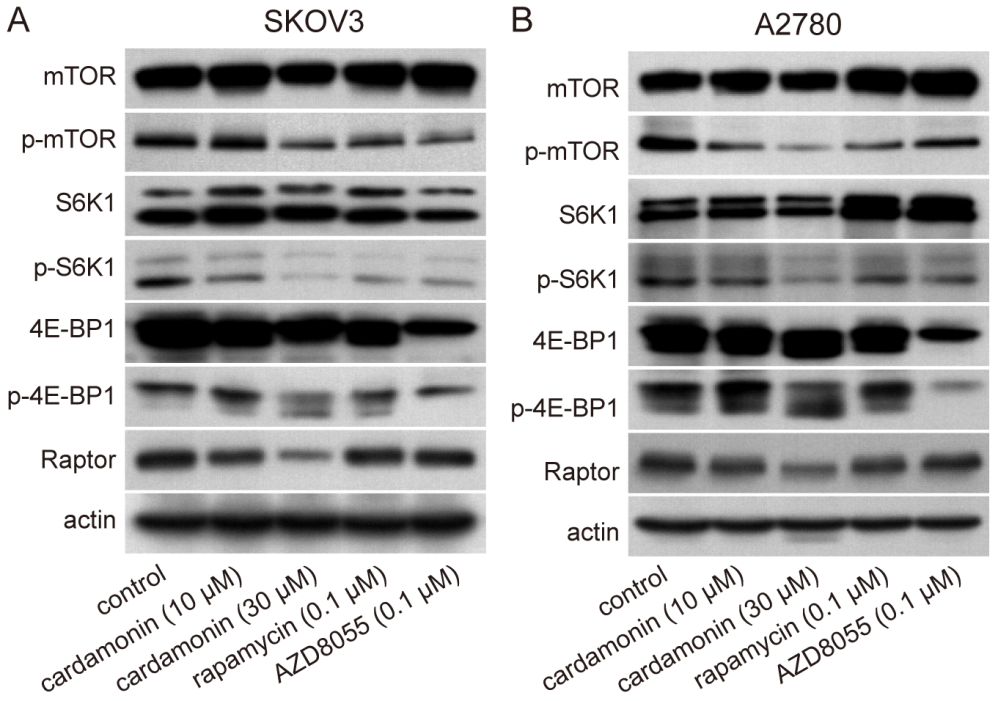





Fig 5A SKOV3 mTOR





Fig 5A SKOV3 p-mTOR





Fig 5A SKOV3 S6K1





Fig 5A SKOV3 p-S6K1





Fig 5A SKOV3 4E-BP1





Fig 5A SKOV3 p-4E-BP1





Fig 5A SKOV3 Raptor





Fig 5A SKOV3 actin
