# Supplementary material for: Cardamonin suppresses mTORC1/SREBP1 through reducing Raptor and inhibits de novo lipogenesis in ovarian cancer
Source: PLoS One. 2025 May 2;20(5):e0322733. doi: 10.1371/journal.pone.0322733 (PMC12047825; doi:10.1371/journal.pone.0322733)
Supplement: S2 File — (ZIP) [file pone.0322733.s006.zip › Original Western Blot Images/Original Western Blot Images/Fig.3D/Original Western Blot Images (For Fig.3D).docx]

Original western blot images for Fig 3D.

The protein blots are imaged by X-ray film exposure. The blots which marked with red frame are used for figure preparation.

Fig 3D


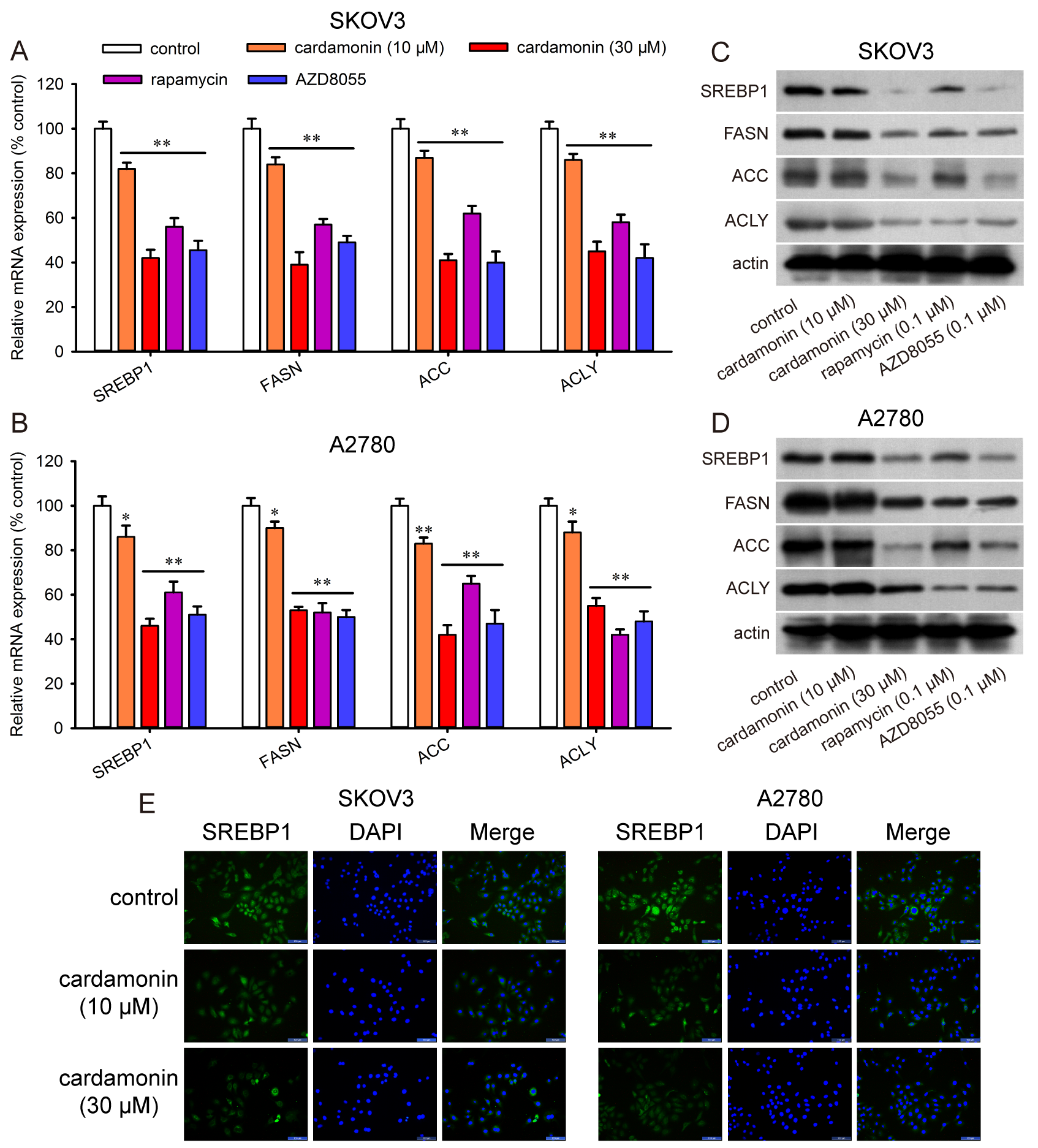





Fig 3D A2780 SREBP1





Fig 3D A2780 FASN





Fig 3D A2780 ACC





Fig 3D A2780 ACLY





Fig 3D A2780 actin
