# Supplementary material for: Cardamonin suppresses mTORC1/SREBP1 through reducing Raptor and inhibits de novo lipogenesis in ovarian cancer
Source: PLoS One. 2025 May 2;20(5):e0322733. doi: 10.1371/journal.pone.0322733 (PMC12047825; doi:10.1371/journal.pone.0322733)
Supplement: S2 File — (ZIP) [file pone.0322733.s006.zip › Original Western Blot Images/Original Western Blot Images/Fig.3C/Original Western Blot Images (For Fig.3C).docx]

Original western blot images for Fig 3C.

The protein blots are imaged by X-ray film exposure. The blots which marked with red frame are used for figure preparation.

Fig 3C


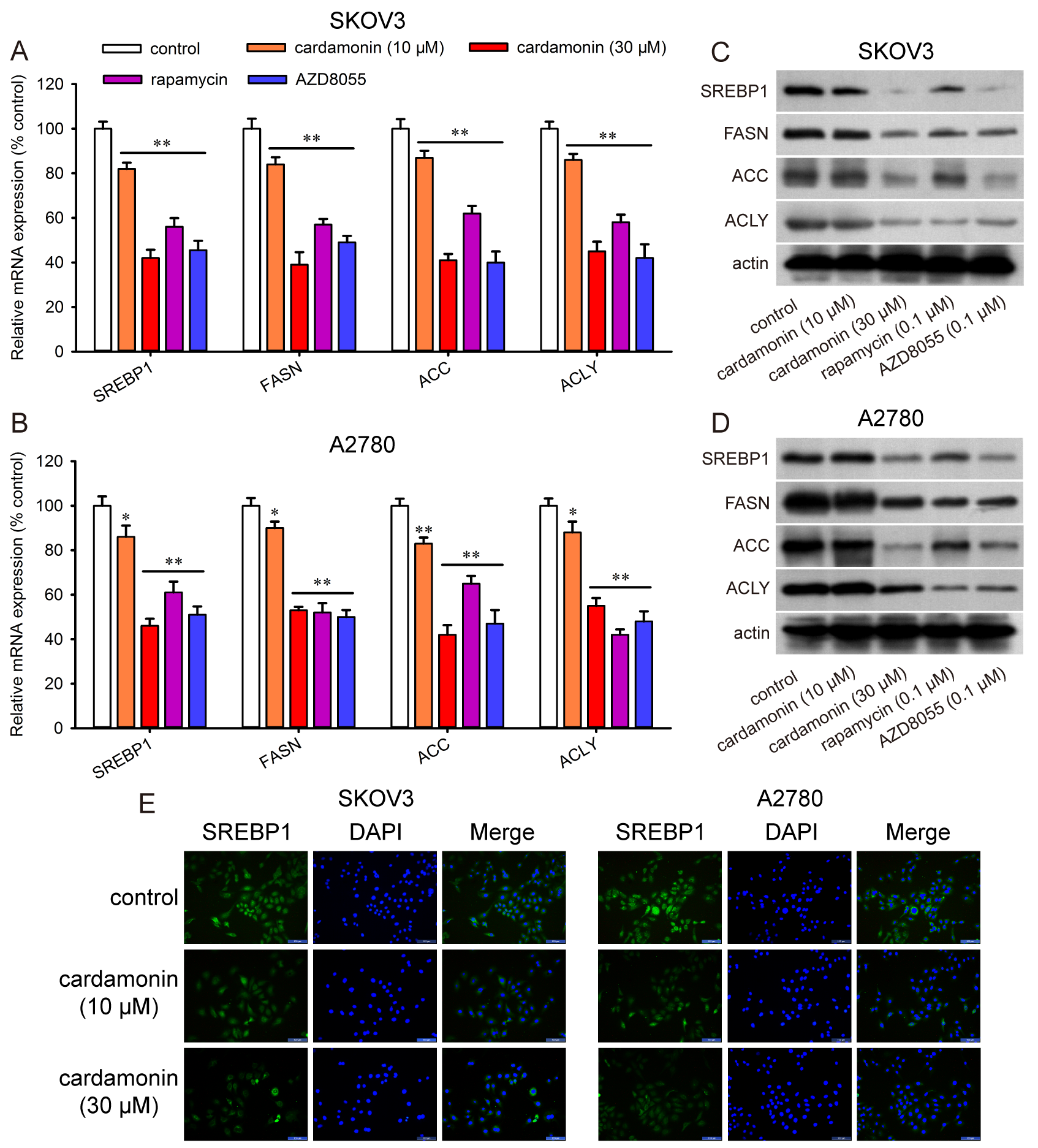





Fig 3C SKOV3 SREBP1





Fig 3C SKOV3 FASN





Fig 3C SKOV3 ACC





Fig 3C SKOV3 ACLY





Fig 3C SKOV3 actin
